# Totality of evidence of the effectiveness of repurposed therapies for COVID‐19: Can we use real‐world studies alongside randomized controlled trials?

**DOI:** 10.1111/cts.13591

**Published:** 2023-08-28

**Authors:** Jaap Mandema, Hugh Montgomery, Louis Dron, Shuai Fu, Estelle Russek‐Cohen, Christina Bromley, Samer Mouksassi, Amy Lalonde, Aaron Springford, Larry Tsai, Phil Ambery, Doug McNair, Nawab Qizilbash, Stuart Pocock, Névine Zariffa

**Affiliations:** ^1^ Certara Princeton New Jersey USA; ^2^ UCL London UK; ^3^ Cytel Vancouver Canada; ^4^ Certara Shanghai China; ^5^ ERCStatLLC Rockville Maryland USA; ^6^ Oyanalytika, Inc. Potomac Maryland USA; ^7^ Certara Cairo Egypt; ^8^ Lilly Indianapolis Indiana USA; ^9^ Cytel Toronto Canada; ^10^ Genentech South San Francisco California USA; ^11^ AstraZeneca Gothenburg Sweden; ^12^ Bill and Melinda Gates Foundation Seattle Washington USA; ^13^ OXON Epidemiology Madrid Spain; ^14^ London School of Hygiene and Tropical Medicine London UK; ^15^ NMD Group Inc. Bala Cynwyd Pennsylvania USA

## Abstract

Rapid and robust strategies to evaluate the efficacy and effectiveness of novel and existing pharmacotherapeutic interventions (repurposed treatments) in future pandemics are required. Observational “real‐world studies” (RWS) can report more quickly than randomized controlled trials (RCTs) and would have value were they to yield reliable results. Both RCTs and RWS were deployed during the coronavirus disease 2019 (COVID‐19) pandemic. Comparing results between them offers a unique opportunity to determine the potential value and contribution of each. A learning review of these parallel evidence channels in COVID‐19, based on quantitative modeling, can help improve speed and reliability in the evaluation of repurposed therapeutics in a future pandemic. Analysis of all‐cause mortality data from 249 observational RWS and RCTs across eight treatment regimens for COVID‐19 showed that RWS yield more heterogeneous results, and generally overestimate the effect size subsequently seen in RCTs. This is explained in part by a few study factors: the presence of RWS that are imbalanced for age, gender, and disease severity, and those reporting mortality at 2 weeks or less. Smaller studies of either type contributed negligibly. Analysis of evidence generated sequentially during the pandemic indicated that larger RCTs drive our ability to make conclusive decisions regarding clinical benefit of each treatment, with limited inference drawn from RWS. These results suggest that when evaluating therapies in future pandemics, (1) large RCTs, especially platform studies, be deployed early; (2) any RWS should be large and should have adequate matching of known confounders and long follow‐up; (3) reporting standards and data standards for primary endpoints, explanatory factors, and key subgroups should be improved; in addition, (4) appropriate incentives should be in place to enable access to patient‐level data; and (5) an overall aggregate view of all available results should be available at any given time.

**Study Highlights**

**WHAT IS THE CURRENT KNOWLEDGE ON THE TOPIC?**

There is controversy regarding the role of real‐world data studies (RWS) in the generation of evidence relative to randomized clinical trials (RCTs). During coronavirus disease 2019 (COVID‐19), both were deployed in large numbers which allowed us to conduct a systematic learning review.

**WHAT QUESTION DID THIS STUDY ADDRESS?**

We first created a re‐useable data asset based on publicly available data sources. We then addressed the following: (i) Do RWS and RCTs of repurposed therapies for pandemics generate results which are consistent in scale and direction? (ii) What features of the study population and of study design/analysis might explain any differences in such results? (iii) What is the timecourse of results as they are generated?

**WHAT DOES THIS STUDY ADD TO OUR KNOWLEDGE?**

Across 249 RWS and RCTs of eight repurposed drugs for COVID‐19 treatment (i) RWS had greater heterogeneity than RCTs for the mortality endpoint and (ii) effect size was exaggerated in RWS when compared to RCTs – an observation only partly explainable by a few study factors. In terms of time‐course analysis, large RCTs, preferably platform trials, provide the most rapid and reliable evidence‐base.

**HOW MIGHT THIS CHANGE CLINICAL PHARMACOLOGY OR TRANSLATIONAL SCIENCE?**

Our analysis suggests that reliable detection of benefit of a repurposed drug for treatment of a novel pandemic infection is best performed by the systematic deployment of large RCTs, especially platform trials. In addition, our analysis serves as an example of how well‐organized, curated aggregated summary‐level information across hundreds of trials can be used to evaluate important questions. The limitations encountered further emphasize the need for sharing of patient‐level data to improve speed and clarity of inference to stem the impact of future pandemics.

## INTRODUCTION

The world remains vulnerable to novel pandemics. Strategies for rapid and robust evaluation of the efficacy and effectiveness of existing pharmacotherapeutic interventions (repurposed treatments) will thus be required. Comparative observational “real‐world” studies (RWS) played such a role in the coronavirus disease 2019 (COVID‐19) pandemic, especially early on.^1^ Although initially small and performed rapidly under challenging circumstances,^2^ they were nonetheless instrumental in clarifying the clinical manifestation following infection and highlighting the disproportionate impact of COVID‐19 on minority populations.^3^ Ultimately, over 1600 peer‐reviewed RWS publications appeared.^4^

Formal randomized interventional controlled clinical trials (RCTs; considered the gold‐standard for evidence generation^5^), some as part of large adaptive platform studies^6^, appeared later. Worldwide, over 3000 were registered,^7^ yielding 480 publications.^8^ The majority of these studies failed to reach a definitive conclusion due to low sample size, or delays in recruitment and publication of results.^9^ However, a few large platform trials did provide useful evidence.^10^

The application of both RWS and RCT approaches to COVID‐19 treatment offers a unique opportunity to objectively evaluate their relative contributions. In turn, this informs improved strategies of evidence generation for future pandemics. To date, the question of whether and how to use the information from RWS to improve patient outcomes has been based largely on qualitative assessment with no unifying viewpoint.^2^, ^5^ A quantitative analysis of three treatments described as a meta‐epidemiological study suggested reasonable concordance, particularly for dichotomous outcomes.^11^

Here, we expand on that work to provide quantitative analysis of the degree of alignment in the impact of eight treatments on COVID‐19 mortality, between RCT and RWS. We first establish the all‐cause mortality differences between these study types within and across treatments, before identifying variables which may account for these differences. We then assess how evidence accumulated over time since the start of the pandemic, using this analysis to draw conclusions regarding the relative contribution of RWS and RCTs to identify effective therapies. Finally, we use these quantitative assessments to inform recommendations to improve both speed and quality of decision making for future pandemics.

## METHODS

Full details on statistical methods and data selection processes are covered in Appendix S1: Supplements A–E. We briefly summarize the study and variable selection methods below along with the statistical analysis.

### Study selection

We included eight treatments with results from studies comparing treatment + unrestricted standard of care (SOC) versus SOC, with a total of greater than 500 patients across greater than or equal to five independent unique studies reporting all‐cause mortality for each of RWS and RCTs. Studies were included independent of treatment type, disease severity, time of publication, or quality metrics.

Our data source for the analysis endpoint as well as the explanatory factors used in the modeling was the CODEx COVID‐19 database (https://codex.certara.com/codex/covid‐19/). It contains a live stream of results from publicly available peer‐reviewed journals (via PUBMED and LitCovid), pre‐print archives (medRxiv, bioRxiv, and ResearchSquare), and trial registries (United States, European Union, Japan, and China). For each selected study, a curation and harmonization process involving natural language processing and human review is deployed resulting in machine readable data that describe in detail the study design elements, analysis methods, results, endpoints, and covariates, that enable cross‐study aggregate analyses (see Appendix S1: Supplement A for additional details). We based our analysis on the April 5, 2022, version, which contains curated and quality‐controlled summary‐level data for studies reported before February 1, 2022. In brief, in that version, 22,056 sources (out of a total of 251,260 since the start of the pandemic) were manually reviewed for potential relevant clinical information on risk factors, treatments, and vaccines. A total of 1941 featured the evaluation of treatments (rather than vaccines or risk factors) of which 1121 (492 RCTs and 629 RWS) were flagged for inclusion into the database. The detailed reasons for exclusion are shown in Appendix S1: Supplement A.

### Modeling variables: Analysis endpoint and explanatory factors

Individual studies reported the impact of repurposed treatments on mortality and disease severity (e.g., clinical scales, intensive care unit admission, mechanical ventilation, and/or duration of hospital stays). As definitions of disease severity evolved during the course of the pandemic, and not all studies reported duration of hospital stay, all‐cause mortality was chosen as the most complete and unambiguously reported endpoint for analysis.

Explanatory factors (summarized in Table 1, and fully listed and defined in Appendix S1: Supplement C) were grouped as (1) study type (RWS or RCT), (2) study‐level factors (e.g., quality metrics, sample size, blinding, analysis methods, and time of publication), and (3) population‐level factors (e.g., disease severity, age, sex, and comorbidities).

**TABLE 1:** Explanatory variables available for assessment and tested for inclusion in the multivariate regression models.

Study‐level factors	
RWS	RCTs
Quality metrics: Confounding and immortal time bias Analysis methods to account for confounding bias Availability of results (prior to, or after the earlier of RECOVERY or SOLIDARITY)	Quality metrics: Cochrane assessment and blinding
**RWS and RCTs**	
Quality metrics: preprint or peer‐review journal, study size, and region Time of mortality assessment Dose (low, medium, and high^a^) Timing of study conduct and results: Study start, mid‐point of study (defined as the central date between reported study start and end of follow‐up, timing of publication	

Population‐level factors: RWS and RCTs
Disease severity at entry (no oxygen, oxygen by simple face mask and oxygen by mechanical means) Differential in key variables between treatment arms: Age (mean difference expressed in years) Sex (% difference in male participants) Diabetes (% difference in patients with diabetes) Hypertension (% difference in patients with hypertension)

Abbreviations: RCT, randomized‐controlled trial; RWS, real‐world studies.

^a^ Full details on doses are available in Appendix S1: Supplement C, Table S3.

### Quality factors: Bias assessment

Specific explanatory factors were developed to account for variability in study quality. RWS were evaluated for the risk of imbalance in known confounders and for immortal time bias (which results if patients cannot experience an outcome within a follow‐up period, making them “immortal” during that time).^12^ These two metrics were selected based on the potential to impact inference. For each quality metric, a common quantitative approach was developed to rank the studies as being of higher, intermediate, or lower quality (tiers 1, 2, and 3, respectively). See Appendix S1: Supplement B for algorithm details.

For RCTs, risk of bias assessment was taken from an existing living review of COVID‐19 RCTs conducted by the Cochrane group, utilizing the Cochrane RCT Risk of Bias assessment tool.^8^ The Cochrane category names were mapped to tier 1, 2, and 3 for ease of interpretation. In addition, blinding, region, and sample size were included as explanatory factors.

### Statistical analysis

Odds ratio for all‐cause mortality (OR‐M) was the primary endpoint and all studies were pooled for analysis. A multivariable mixed model meta‐regression was used^13^, ^14^ for the evaluation of (1) the differential between RWS and RCTs, (2) the impact of explanatory variables on this differential, and (3) the impact of explanatory variables on between‐study heterogeneity. A maximum likelihood testing strategy was used, with a *p* value of less than 0.05 indicating statistical significance. The final multivariable regression analysis retained only explanatory factors which were significant (*p* < 0.05). To account for collinearity among explanatory variables, these were ranked prior to analysis by clinical relevance starting with dose and disease severity, followed by quality metrics which were of great interest, then other study‐level factors, and last other population‐level factors. The influence of individual studies on the final model was evaluated by assessing the impact on the results when removing studies one at a time. The impact of collinearity among the set of explanatory variables was assessed by re‐testing individual factors in the final analysis.

A mixed effects meta‐analysis approach was used to characterize the timecourse of the aggregated OR‐M using a similar variance structure as the final model above, and fixed effects for combination of treatment and disease severity. Treatment effects were re‐estimated as each new study entered the aggregated analysis. The timecourse was created based on the day of reporting for each result for (1) RCTs alone, (2) RWS alone, and (3) combined RWS and RCTs, each weighted by its between‐study variability. We used December 1 2019, as the start date for the pandemic. From these results, the time at which a clear decision for “benefit” or “no clinical benefit” was determined, and the number of patients before/after these timepoints tabulated. “Benefit” was defined as a greater than or equal to 97.5% probability of the odds ratio (OR) between treated and SOC being less than 1 for at least 2 calendar weeks. “No clinical benefit” was defined as a greater than or equal to 97.5% probability of the OR between treated and SOC being greater than 0.9 for at least 2 calendar weeks. Any other result was deemed indeterminate, and labeled as “no decision.”

The generalized least squares regression function (gnls) and the nonlinear mixed effect regression function (nlme) provided in R (version 3.6.1 or later) were used for the multivariable analyses. The meta (version 5.0‐1) and metafor (version 3.0‐2) packages were used for univariate meta‐analyses.^15^

All co‐authors had access to the datasets and analysis code. Dataset creation was programmed by one analyst and code reviewed with other co‐authors. Statistical analyses were conducted by one analyst and reviewed independently by another. RWS quality metrics were programmed based on quantitative data elements from the original reports and the algorithms defined in Appendix S1: Supplement B; these were reviewed by a subset of co‐authors. All underlying data are publicly available upon request.

## RESULTS

### Study selection

**FIGURE 1:**
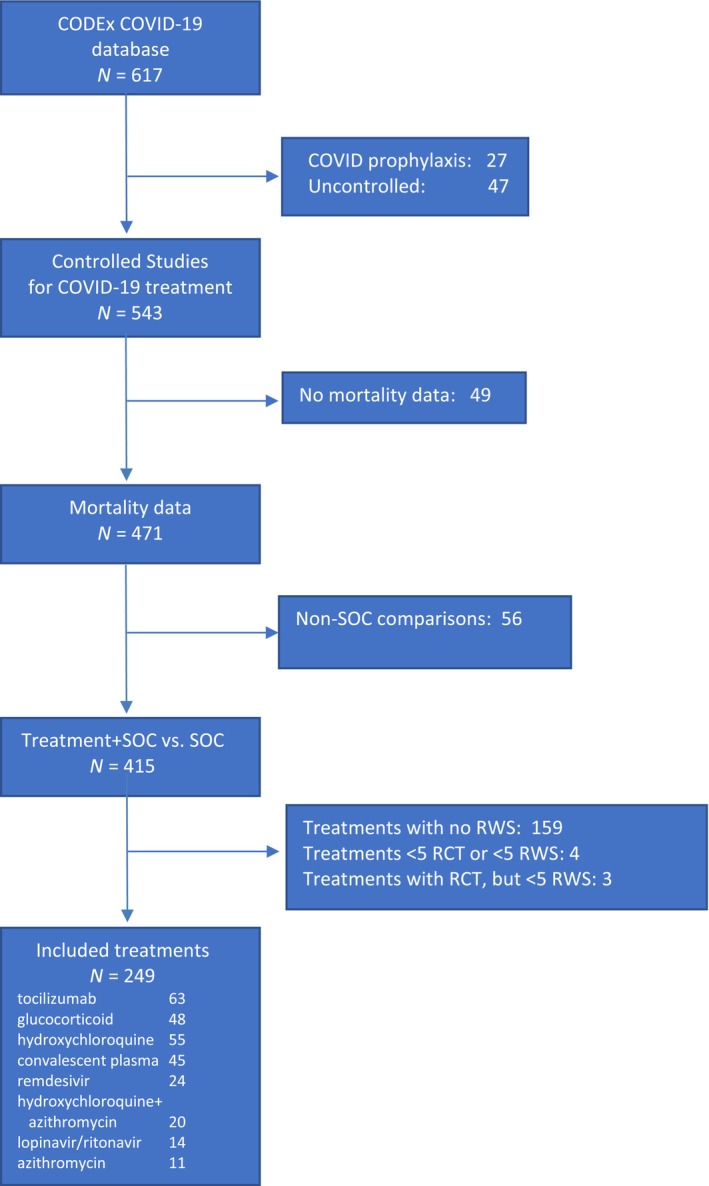
Study selection. COVID‐19, coronavirus disease 2019; RCTs, randomized controlled trials; RWS, real‐world studies; SOC, standard of care.

An overview of the study selection process is presented in Figure 1. A full list of included studies, their sample size, disease severity, time of mortality analysis, publication date, mean age, treatment dose, and bias assessment is provided in Appendix S1: Supplement D.

In brief, a total of 543 studies assessing 155 different drugs or drug combinations was identified in the CODEx COVID‐19 database, of which 471 unique studies reported mortality. Of these, 415 compared treatment + SOC to SOC: 226 RCTs and 189 RWS. Eight treatments met the threshold for inclusion: tocilizumab (number of studies, *N* = 63), hydroxychloroquine (*N* = 55), glucocorticoids (*N* = 48), convalescent plasma (*N* = 45), remdesivir (*N* = 24), hydroxychloroquine + azithromycin (*N* = 20), lopinavir/ritonavir (*N* = 14), and azithromycin (*N* = 11). Across these eight treatments, there were data from 249 studies (154 RWS and 95 RCTs) representing 312,865 patients (236,781 RWS and 76,084 RCT). Five of the therapies had greater than 50% of their total evaluable sample size from RWS, with the exception of azithromycin (43%), convalescent plasma (37%), and lopinavir/ritonavir (11%). The greatest number of studies involved tocilizumab (*N* = 63), whereas the largest total number of patients was available for remdesivir (*N* = 119,160). All treatments, with the exception of azithromycin, had more patients in RWS available than in RCTs.

Of the available 154 RWS included within our analysis, 60 (39%) were considered to have used the best approaches to address known confounders (i.e., tier 1), 42 (27%) to be of intermediate quality (tier 2), and 52 (34%) to be of poor quality (tier 3). With respect to immortal time bias, nearly half (*N* = 76, 49%) were considered tier 3, with 32 studies (21%) in tier 2, and 46 (30%) in tier 1. Most RCTs (*N* = 66 of 95, 69%) were assessed as intermediate quality (tier 2), with similar numbers in tiers 1 and 3 (*N* = 13, 14% vs. *N* = 10, 11%, respectively). Six RCTs were nonevaluable for bias assessment. Full details are provided in Appendix S1: Supplement B.

Mortality was most frequently reported at week 4 (day 28) post‐study initiation: 72 RWS (47%) and 52 (55%) of RCTs. Overall, a higher proportion of RCTs than RWS reported mortality at less than or equal to 3 weeks: *N* = 33 (35%) versus *N* = 36 (23%). Conversely, a higher proportion of RWS than RCTs reported mortality greater than or equal to week 5: *N* = 46 (30%) versus *N* = 10 (11%).

### Overall (unadjusted) results – RWS versus RCT

**FIGURE 2:**
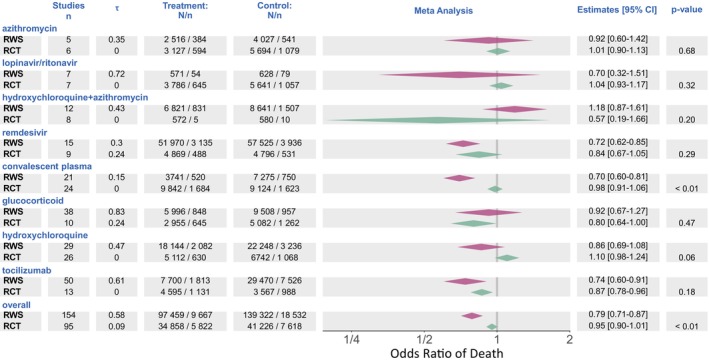
Mortality by treatment and study type (unadjusted analysis). The estimate for RCTs are shown in green and nonrandomized studies (RWS) in red. The *n* indicates the number of studies and *τ* the between study variability. The *p* values represent the interaction test between RWS and RCT for a given therapy or treatment class. Overall denotes the estimate for RCTs relative to RWS for all eight treatments. CI, confidence interval; RCTs, randomized controlled trials; RWS, real‐world studies.

Figure 2 provides the estimates of treatment effects for RWS and for RCTs based on a univariate random effects meta‐analysis prior to any adjustments for explanatory variables. In this pooled analysis, survival improvements for all therapies combined were statistically significantly greater in nonrandomized than randomized trials (OR_RWS_: 0.79, 95% CI = 0.71–0.87 vs. OR_RCT_: 0.95, 95% CI = 0.90–1.01, *p* = < 0.01). Considering individual treatments, survival benefits appeared greater in RWS than in RCTs for all interventions except glucocorticoids, and hydroxychloroquine in combination with azithromycin. Variability between‐studies, represented by τ, was lower in RCTs (0.09) than RWS (0.58) for overall treatments, and this was consistent for each individual treatment assessed.

### Evaluation of explanatory factors

Given the observed differences between RWS and RCT in the included studies, a set of explanatory factors (full list in Appendix S1: Supplement C) were evaluated to see whether they influenced the gap between RCT and RWS evidence with respect to mortality benefit. A pooled multivariable analysis of both RWS and RCTs was conducted to identify the factors that might explain the differential between RWS and RCT evidence. The multivariable analysis retaining significant explanatory variables provides the basis for estimation of the adjusted treatment effects. The results are provided in Appendix S2: Supplement F. We first discuss the results related to the explanatory factors, then the overall results describing the estimates of treatment effects.

Concordant with results in Figure 2, study type (i.e., RWS vs. RCT) has a global impact across all treatments (*p* < 0.01), with no overall treatment by study type interaction (*p* = 0.22). We first assessed dose regimens and disease severity. There was no significant global impact of dose on treatment effect (*p* = 0.08) and no treatment by dose interaction (*p* = 0.21). A significant treatment by disease severity interaction was observed in glucocorticoids (*p* < 0.01) and remdesivir (*p* = 0.03). For glucocorticoids, there was a significant difference across three levels of disease severity, with the largest treatment effect being observed in the most severely ill patients. Conversely, for remdesivir, the treatment effect was largest in mild/moderate and moderate patients. No other treatments included within our analysis demonstrated a treatment by disease severity interaction.

Appendix S2: Supplement F also includes the results of the evaluation of the explanatory factors that were retained in the final analysis. These suggest that the difference in treatment effect between RWS and RCTs was significantly impacted by the time of mortality follow‐up (*p* < 0.01) in RWS, where shorter time periods of weeks 1 and 2 combined further exaggerated the treatment effect in RWS relative to RCTs. Neither the risk of bias assessment for RCTs nor immortal time bias for RWS had any impact on results. However, RWS with high confounding bias (tier 3) showed a smaller treatment effect than did RWS studies with better control measures for confounding bias in tiers 1 and 2 (*p* < 0.01). (This counter‐intuitive result is likely due to more severe patients being allocated to treatment + SOC rather than to SOC in tier 3 RWS.)

Considering RWS on their own, a few explanatory variables were associated with an impact on the treatment estimates. As a group, the factors that follow explain ~40% of the differential in inference between the two study types. Smaller RWS studies (*N* < 200) exaggerated the treatment effect relative to larger (*N* ≥ 200) RWS studies (*p* = 0.03). An imbalance in age between active and control groups impacted upon the treatment effect in RWS without any bias mitigation (i.e., reporting crude means), with RWS that had older participants in the control than treated groups showing a smaller treatment effect. Similar results were found for imbalance in the proportion of male or of critically ill patients (*p* < 0.01 for both). These same variables had no impact on the RCT based treatment estimates – in fact, other than disease severity, no other explanatory factor was observed to have a significant impact on inference in RCTs.

No other explanatory factor had an impact on the differential in treatment effect observed between the RWS and the RCTs. Additional sensitivity analyses to evaluate the potential impact of both quality measures together (i.e., RWS with higher quality for both confounding and immortal‐time bias) for RWS did not reveal a statistically significant separation from the lower quality RWS.

**FIGURE 3:**
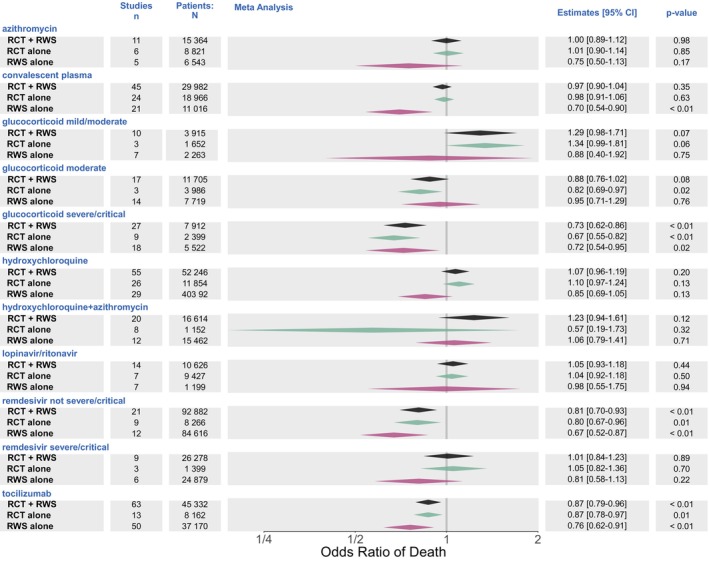
Mortality by treatment and study type (multivariable analysis). CI, confidence interval; RCT, randomized‐controlled trial; RWS, real‐world studies.

Estimates for the treatment effects were also evaluated in our multivariable analysis, retaining disease severity for glucocorticoids and remdesivir as well as the statistically significant factors described above (Figure 3). The prior directional differential between the results of the RCTs and RWS from the unadjusted univariate analysis remains, and the RWS as a group remain more heterogenous than RCTs as a group. Estimates of treatment effects were based on RCTs alone, RWS alone, and a weighted average of the two (with RWS down weighted due to their high between‐study variability).

Based on the totality of evidence from both RCTs and RWS, the only statistically significant therapies for mortality reduction were tocilizumab (OR: 0.87, 95% CI = 0.79–0.96), remdesivir in mild and mild/moderate patients (OR: 0.81, 95% CI = 0.70–0.94), and glucocorticoids in severe/critical patients (OR: 0.70, 95% CI = 0.59–0.83). For all three treatments, the inference on treatment effect is not changed by the combination of RWS + RCT evidence when compared to RCT evidence alone.

The results are more mixed for other treatment estimates. For example, glucocorticoid administration in moderate severity patient populations was associated with a statistically significant reduction in mortality when considering RCT evidence alone (OR: 0.82, 95% CI = 0.69–0.97) but was the effect attenuated when integrated with RWS evidence (OR: 0.88, 95% CI = 0.76–1.01). In contrast, for the combination of hydroxychloroquine and azithromycin, the uncertainty in treatment effect estimates for RCT evidence alone (OR: 0.57, 95% CI = 0.19–1.73) was reduced with the combined RWS and RCT evidence but did not reach statistical significance (OR: 1.23, 95% CI = 0.94–1.61).

### Analysis of accumulating evidence

We further evaluated the pace of information generated during the COVID‐19 pandemic by constructing a longitudinal cumulative meta‐analysis for RCTs alone and for RWS and RCTs in combination. This allows an evaluation of “real‐time” aggregate information for each treatment, with RWS and RCTs weighted by the between‐study variability in each case. We begin on day 1, defined as December 1, 2019, and observed how estimated treatment effects on mortality evolve during the course of the pandemic. We categorize instances where early RWS lead to earlier and concordant decisions and where they do not. Appendix S2: Supplement G provides full results for each treatment. We briefly describe two of the more interesting examples below.

**FIGURE 4:**
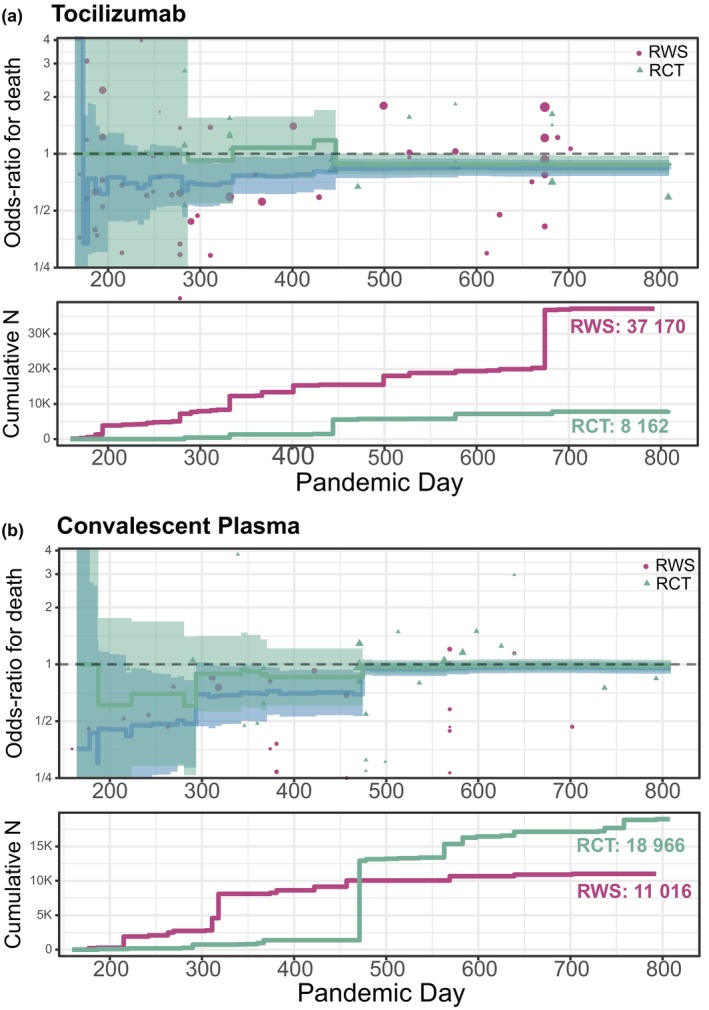
(a) Cumulative evidence of tocilizumab treatment effect on mortality since the start of the pandemic. The solid blue line in the top panel shows the cumulative estimate of the treatment effect of tocilizumab from both RCT and RWS with its 95% CI. The solid green line represents the accumulative evidence for tocilizumab from RCTs alone (i.e., no RWS) with its associated CI. The result of every included RWS (magenta circles) and RCT (green triangles) is also shown. Symbol size is inversely related to the precision of the estimate from each study. The bigger the symbol, the more precise the estimate. The bottom panel shows the cumulative sample size for RCTs (green) and RWS (magenta). (b) Cumulative evidence of convalescent plasma effect on mortality since the start of the pandemic. The solid blue line in the top panel shows the cumulative estimate of the treatment effect of convalescent plasma from both RCT and RWS with its 95% CI. The solid green line represents the accumulative evidence for convalescent plasma from RCTs alone (i.e., no RWS) with its associated CI. The result of every included RWS (magenta circles) and RCT (green triangles) is also shown. Symbol size is inversely related to the precision of the estimate from each study. The bigger the symbol, the more precise the estimate. The bottom panel shows the cumulative sample size for RCTs (green) and RWS (magenta). RCT, randomized‐controlled trial; RWS, real‐world studies.

In the case of tocilizumab (Figure 4a), early RWS suggested a therapeutic benefit, but with wide CIs due to the lack of consistency between the RWS studies (*τ*) which persisted throughout. The treatment effect was reduced and became more precise when the initial RCTs were reported. The wide CI indicates substantial between‐study heterogeneity. However, waiting for a clear decision based on RCTs alone created a lag of 440 days. A contrary example involves convalescent plasma (Figure 4b), where RWS were the only source of information for a considerable duration (day 1–day 280). The RWS suggested an overwhelmingly positive effect, which disappeared when the RCTs reported. Additional details for the other therapies are available in Appendix S2: Supplement G.

The time at which a first decision on clinical benefit is achieved and the type of decision are summarized in Table 2 for all therapies investigated. Overall, clear shifts in evidence are reached when larger RCTs read out, typically either RECOVERY on its own, or a combination of both RECOVERY and SOLIDARITY. No other clear pattern is discernible. A faster decision is achieved for tocilizumab and remdesivir in mild, mild/moderate, or moderate patients (not severe/critical) when RCT and RWS evidence is combined compared to RCTs alone – gain of 194 days and 54 days, respectively. A faster conclusion of no benefit is obtained for the combination of azithromycin and hydroxychloroquine at 239 days using RWS and RCT results compared to achieving no decision up to over 800 days based on RCTs alone (Appendix S2: Supplement G). However, apparently ineffective treatments show either no impact of RWS on the speed of decision (e.g., azithromycin and hydroxychloroquine), or faster decisions based on RCTs alone (e.g., lopinavir/ritonavir).

**TABLE 2:** Timing of clear decisions from RCTs alone and in combination with RWS.

Treatment	RCT evidence alone		RCT + RWS evidence	
	Decision	Time (days since December 1, 2019)	Decision	Time (days since December 1, 2019)
Azithromycin	No clinical benefit	384	No clinical benefit	384
Convalescent plasma	No clinical benefit	566	Benefit/ No benefit	218/642
Glucocorticoid mild/moderate	No clinical benefit	218	No clinical benefit	218
Glucocorticoid moderate	Benefit	218	Benefit	218
Glucocorticoid severe/critical	Benefit	218	Benefit	218
Hydroxychloroquine	No clinical benefit	239	No clinical benefit	239
Hydroxychloroquine+ azithromycin	No decision	–	No clinical benefit	239
Lopinavir/ritonavir	No clinical benefit	328	No clinical benefit	549
Remdesivir not severe/critical	Benefit	328	Benefit	274
Remdesivir severe/critical	No Decision	–	No decision	–
Tocilizumab	Benefit	474	Benefit	280

*Note*: For the decision on each treatment, “Benefit” is defined as the probability of a mortality odds‐ratio <1 being >0.975 for at least 2 calendar weeks. "No clinical benefit" is defined the probability of a mortality odds‐ratio >0.9 being >0.975 for at least 2 calendar weeks. "No decision" refers to any treatment which does not meet the criteria for “benefit” or “no benefit”. For the combination of RCT + RWS, evidence from RWS is down‐weighted relative to RCT, owing to the proportionately higher τ, although is not otherwise adjusted on the basis of study effects.

Abbreviations: RCT, randomized‐controlled trial; RWS, real‐world studies.

## DISCUSSION

### RWS versus RCTs for evaluation of COVID‐19 treatment response

For the eight COVID‐19 treatments with sufficient information to compare the two evidence generation approaches, we find that RWS generally overestimate the treatment effect seen in RCTs. In some instances, such as tocilizumab, the RWS effect is directionally aligned to that later seen in RCTs, whereas in other therapies, such as convalescent plasma, it is nonconcordant with the totality of RCT evidence as of May 2022.

Two exceptions to the observation that RWS overestimate treatment effects were glucocorticoids and hydroxychloroquine + azithromycin. For glucocorticoids, it is possible that the results represent discrepancies in the distribution of patients' baseline disease severity between the RWS and RCTs. Among RCTs of glucocorticoids, 25.5% of participants were of critical status, and 20.5% were of mild/moderate status. In contrast, only 4% of patients were of critical status in RWS, and 49.8% were of moderate status. As glucocorticoids appear more efficacious in patients with greater disease severity, the differences observed in our univariate analysis may be in part attributable to the higher proportion of patients with severe disease among RCTs. This demonstrates the importance of having data reported by disease severity in future pandemics.

Some of the difference between RWS and RCT findings was explained by factors, such as the timepoint at which mortality was assessed in the RWS, the sample size of the study, the quality with regard to risk of bias due to confounding in RWS, and imbalances in age, sex, and disease. This was most notable among those RWS which did not provide methodological solutions to minimize differences between treated and control group population covariates. We could not identify a set of explanatory factors that fully explain the magnitude of the differential between RWS and RCTs, nor the large degree of heterogeneity seen in RWS. Whether this is due to challenges in reporting and data capture of RWS^16^ leading to unreported variables of key prognostic relevance is unclear. Other groups have similarly identified gaps between RWS and RCT in other therapeutic areas, which are not explained by the available reported variables.^17^

With respect to the accumulation of evidence for treatment of COVID‐19, RWS reported findings earlier than RCTs, but also had greater between‐study heterogeneity. This differential might be reduced in the future if we prepared now for rapid RCT/platform trial deployment. For most therapies, enhanced stability of treatment effect estimates was only observed when large RCTs reported their results. This finding, in association with the overall observation that, for COVID‐19, RWS tended to overestimate treatment effects raises the question of how best to evaluate emerging evidence in a novel health crisis.

The time to achieve a decision based on the totality of data relating to glucocorticoids (in all patient severity groups), azithromycin, and hydroxychloroquine was similar to an analysis based upon RCTs alone. This indicates that the RWS for these therapies were essentially inconsequential for meaningful treatment effect inference. Reasons for this were largely due to the impact of results from large RCTs reporting early in the pandemic (predominantly from RECOVERY). For corticosteroids, the results trended toward no benefit in all disease severity groups based on the totality of the data before the RECOVERY data was published. Conversely, prior to the large RCTs reporting, RWS suggested a strong benefit of convalescent plasma. Based on the totality of the data, a clear clinical benefit decision would have been achieved for convalescent plasma at day 218 solely based on RWS. It would take until day 642 to have this decision reversed to no clinical benefit after large RCTs had been published and much later than based on RCTs alone. These examples suggest that large RCTs are essential as early as possible during a public health emergency.

It should be noted that these results differ from another quantitative analysis performed by Moneer et al.^11^ The authors conclude good concordance between results from RCTs and from RWS for three treatments (hydroxychloroquine, ritonavir/lopinavir, and dexamethasone) based on a total of 46 RWS and 37 RCTs. In that meta‐epidemiological study, the authors paired RWS and RCTs studying the same treatments, in the same disease severity groups. Some cells were very small (total of 10 cells with only one RCT and one RWS), and many treatments we included were not evaluable within this analysis. For treatments where there is sufficient data to compare our results to theirs, for example, hydroxychloroquine, there is good concordance of results. One possible explanation for the difference in conclusions is the size of the respective databases and the author's early decision to limit their analyses to the three selected treatments.

### Strengths and limitations

A key strength of this work is that it makes use of publicly available data, which will always be accessible as researchers perform studies and report through established channels. In particular, efforts were noted with respect to improving open‐access for literature during the COVID‐19 pandemic.^18^ As such, it is possible to perform this type of analysis without requiring patient‐level data sharing, which has been very limited during the COVID‐19 pandemic for RCTs,^7^ and even more so for RWS.^19^ The size of the dataset (312,865 participants in 249 studies) is substantial, with minimal missing data owing to the outcome assessed, and yields results which are robust as assessed by several sensitivity analyses. Last, the CODEx COVID‐19 database set itself includes many relevant explanatory factors, additional endpoints (such as hospital stay duration, viral clearance, and ordinal performance score change), and is continuously updated and freely available to all researchers. Finally, where other meta‐analysis results are available, our results are concordant. Appendix S1: Supplement A includes the request website for the CODEx COVID‐19 database, which is freely available for researchers looking to replicate or explore further our work.

Our analyses have important limitations. Given the retrospective nature of our work and the fact that results are based on reported and publicly available studies, our study may be underpowered to detect the impact of important explanatory variables and reflects only the treatments selected for analysis. Although overall patient counts are large, some analyses are hampered by a smaller number of studies per therapy, limiting the power of study‐level meta‐regression to identify explanatory factors. It is also subject to publication bias, which may have evolved during the period under study; a distinct possibility with RWS where early publications may have been more permissive than later in the pandemic. Given our analyses are retrospective, we cannot exclude some of the variability observed across RWS comes from the fact they may have had distinctly different research hypotheses, whereas RCTs tend to be more homogeneous in this regard.

Our analysis is based on summary‐level data rather than patient‐level data. As such, assessments are limited regarding duration of symptoms at entry or other features only available with patient‐level data. Related to this point, some important comorbidity subgroups were not uniformly included in study results, and thus could not be assessed. In the setting of summary‐level data, it is difficult to harmonize data (e.g., endpoint definitions) and analytics (e.g., estimands) strategies across the trials.

Potentially influential variables may not have been reported. Throughout the pandemic, there have been substantial regional variations with respect to variants of concern,^20^ healthcare system burden,^21^ vaccination coverage,^22^ and access to medications.^23^ Each of these may have differential influence on the efficacy of treatments as well as the standard of care, particularly in RWS. The multivariable model included an assessment of impact on our results for various aspects related to chronology: (a) study start, (b) mid‐point of study (defined as the central date between reported study start and end of follow‐up), and (c) timing of publication. None of these were retained as significant explanatory variables. Although there were imperfect measures of the important aspects of variants and rollout of vaccination, they were considered. Region was also assessed and not retained.

Our study was limited to the reporting of mortality data and as such the application of these findings to other outcomes of interest is presently uncertain. Further, our analysis was limited to pooled ORs, rather than adjusted HRs, RRs, and ORs, which may influence our findings. Beyond mortality, other endpoints of interest, such as duration of hospital stay or disease severity, could be analyzed to further characterize the overall benefit of treatments, as well as provide information on whether the divergence in RWS and RCT evidence is true in other outcomes. Such research would be hampered by the lack of harmonization of the often‐heterogeneous definitions and analysis methods utilized across these outcome measurements for COVID‐19.^24^

Despite these limitations and given that data sharing at the patient level has seen limited success at scale during the COVID‐19 pandemic,^25^, ^26^ this work exemplifies the type of hypotheses that can be assessed utilizing publicly available data at scale in a retrospective context. A fully prospective framework for aggregate results or full sharing of patient‐level data in an organized fashion would overcome the limitations stated above.

Finally, our overarching recommendations are provided in Table 3.

**TABLE 3:** Key recommendations for future pandemics.

Recommendation	Commentary
Access to real‐time aggregate evidence in its entirety should be provided	Continued uncoordinated efforts in times of crises create challenges for acquiring robust research evidence. Further, having good understanding of accumulating information helps to prioritize treatments for testing in RCTs. Last, policy makers and the healthcare teams would benefit from seeing the level of certainty (or lack thereof) based on all available studies, rather than the few that get media attention
Research allocation for certain studies should be de‐prioritized	Small studies of either type (*n* < 200), RWS that do not address imbalance in known confounders and RWS with short follow up (<2 weeks) have limited value in determining treatment effect on mortality and can be misleading
Platform studies and larger RCTs are overwhelmingly more influential than multiple minor clinical trials. Preparations should be made for their rapid deployment in future pandemics	These large‐scale studies should be in a state of readiness to be initiated, research sites world‐wide engaged and clear coordination mechanisms established ahead of time
Reporting standards for both RCTs and RWS should be improved	For both RCTs and RWS, improving reporting standards of studies with consistently defined subgroups and harmonized disease definition endpoints would be valuable.^27^ For RWS, better adherence to common data formats (such as OMOP for EHR) and common data collection elements for emergent health threats.^28^
Sharing of patient‐level data should be incentivized	Multiple research hypotheses require patient‐level data sharing. Combined with a good strategy to develop, test and apply data standard formats across both RWS and RCTs, much better insights could be developed quickly.

Abbreviations: EHR, electronic health record; OMOP, Observational Medication Outcomes Partnership; RCT, randomized‐controlled trial; RWS, real‐world studies.

## CONCLUSION

Relative to RCTs, a consistent trend of overly optimistic and heterogeneous mortality treatment effect estimates was seen in RWS of eight investigated treatments for patients with COVID‐19. This variability and potential bias remain largely unexplained, although partially influenced by smaller study sample size, there are unaddressed imbalances in known confounders in RWS and differences in outcome reporting time periods. Whereas reporting earlier, the value of RWS on an integrated evidence synthesis was minimal, as the majority of evidence was weighted toward large RCTs. In future emerging health crises, resources and effort should be focused on large well‐conducted RCTs, for which infrastructure might be prepared in advance. Improvements to reporting of RWS and utilization of bias‐reducing methodologies may improve their integration and utility as part of a holistic evidence‐based assessment.

## AUTHOR CONTRIBUTIONS

N.Z., A.L., A.S., C.B., D.M., E.R.‐C., H.M., J.M., L.D., L.T., N.Q., P.A., S.F., S.M., and S.P. wrote the manuscript. J.M., L.D., L.T., H.M., and N.Z. designed the research. J.M. and S.F. performed the research. A.L., A.S., J.M., S.F., S.M., N.Z., E.R.‐C., C.B., and N.Q. performed the analysis of the data.

## FUNDING INFORMATION

The Bill and Melinda Gates Foundation provided funding for the CODEx COVID‐19 database (https://codex.certara.com/codex/covid‐19/). J.M., S.F., S.M., A.S., and N.Z. all received funding from The Bill and Melinda Gates Foundation for their contributions to the study. This work was supported, in whole or in part, by the Bill & Melinda Gates Foundation (Grant Number INV‐019330). Under the grant conditions of the Foundation, a Creative Commons Attribution 4.0 Generic License has already been assigned to the Author Accepted Manuscript version that might arise from this submission.

## CONFLICT OF INTEREST STATEMENT

L.D. reports equity in Cytel. L.T. reports stock ownership and employment with Genentech. N.Z. was Strategic Advisor to FDA, Office of Commissioner (September 2020–September 2021) regarding RWD and COVID‐19. The article reflects N.Z.'s personal views, not those of the FDA. N.Z. was a member of the executive committee of ICODA (International Covid‐19 Data Alliance) during the period of June 2020–December 2022. N.Z. reports personal fees from Bill and Melinda Gates Foundation, Genentech, BMS, ANOVA, ZS Associates, FDA, Cytokinetics, Intelligencia, Friends of Cancer Research, and Pfizer, outside the submitted work (May 2021–2023). N.Z. held/holds varying levels of stock equity in AstraZeneca, GSK, J&J, Merck, Moderna, Pfizer, Sanofi, Takeda, TranslateBio, Vaxart, Vir, and Inovio during the period May 2021–May 2023. P.A. reports stock ownership and employment with AstraZeneca. H.M. reports receiving fees for consultancy, honoraria, travel, and study committee from AstraZeneca relating to the use of new therapies for COVID‐19 treatment and prophylaxis. N.Q. reports ownership and employement with Oxon Epidemiology. All other authors declared no competing interests for this work.

## Supporting information


Appendix S1

Appendix S2
