# Supplementary material for: Totality of evidence of the effectiveness of repurposed therapies for COVID‐19: Can we use real‐world studies alongside randomized controlled trials?
Source: Clin Transl Sci. 2023 Aug 28;16(10):1842–55. doi: 10.1111/cts.13591 (PMC10582658; doi:10.1111/cts.13591)
Supplement: Supplementary file 1 — Appendix S1 [file CTS-16-1842-s002.docx]

**Supplementary Material**

**Totality of Evidence of the effectiveness of repurposed therapies for COVID-19: Can we use Real-World Studies alongside Randomised Controlled Trials?**

Short Title

Role of RWS and RCTs in Evidence Generation: COVID19 Therapeutics

## Supplements A-E

## Supplement A: COVID-19 database search strategy and included sources

The objective of the COVID-19 clinical outcome database project was to curate and augment all publicly available aggregate data from studies evaluating pharmacological interventions, risk factors, or biomarkers for COVID-19 disease progression, treatment, and prophylaxis. The data is freely available for research purposes. The data is accessible through a web-based interface (CODEx) to facilitate access, to review, summarize, and graph study data, and to perform pair-wise or network meta-analyses (<https://codex.certara.com/codex/covid19/>).

### Data sources

Studies with relevant information were identified by a search of the following sources:

- Pubmed (<https://pubmed.ncbi.nlm.nih.gov/>) including LitCovid indexed papers for COVID-19 (<https://www.ncbi.nlm.nih.gov/research/coronavirus/>)
- Papers indexed for COVID-19 on preprint servers medRxiv, bioRxiv, and ResearchSquare
- studies indexed on the following registries
  - clinicaltrials.gov ([https://clinicaltrials.gov](https://clinicaltrials.gov/ct2/results?cond=COVID-19))
  - ChiCTR (<http://www.chictr.org.cn/enIndex.aspx>)
  - EU clinical trials register (<https://www.clinicaltrialsregister.eu/>)
  - Japanese trials registry (<https://www.umin.ac.jp/ctr/>)
- FDA/EMA summary basis of approval
- Meta-analyses and systematic reviews

The first search was done on May 7, 2020 and updated every week thereafter. The search procedure for the data sources is outlined in the figure below.

Figure S1: Search procedure for data sources included within the CODEx COVID-19 Database

Detailed source information was downloaded from the websites weekly. New sources were identified and parsed for key information. The key information for all sources identified was extracted automatically and maintained in a source database (SDB) that includes fields to characterize the full reference and abstract, URL, digital object identifier (DOI), study name and registry id number, the date on which the source was first identified and the place the source was identified. Sources that provide information on the same study were linked by study name and study registry ID.

Given the large number of sources with potential information of interest, a NLP algorithm was used to index the sources with regards to the treatments being evaluated, type of study (randomised controlled trial (RCT), observational real-world study (RWS), meta-analysis, review) and other variables. The algorithm used all source information including title, abstract and MESH terms. A priority review list was generated on basis of the algorithm that flags all sources with a pharmacological intervention in title/abstract or that report on a RCT and not a systematic review or meta-analysis. The identification of relevant sources by the algorithm was >99% based on a review of a test set of sources during the first months of the pandemic. Each source that was flagged by the algorithm is further reviewed manually for inclusion or exclusion into the COVID-19 outcomes database.

At the highest level the sources were categorized in 5 groups:

- **treatment:** study evaluating treatments of COVID-19
- **vaccine:** study evaluating vaccines for COVID-19
- **prophylaxis:** study evaluating treatments for prophylaxis of COVID-19
- **risk factors:** study evaluating risk factors for COVID-19
- **biomarkers:** study evaluating biomarkers for COVID-19
- none of the above

Information of the reason of exclusion of the source from the COVID-19 outcomes databases was documented. The sources categorized in the treatment group were excluded from the COVID-19 outcomes database if:

- The indication was not treatment of COVID-19.
- The study type was not a RCT or RWS (i.e., a protocol description, review, or meta-analysis)
- There was no control group (only one treatment cohort in the study).
- The intervention was not a pharmacological intervention with a defined mechanism of action. Herbal and traditional Chinese medicines were excluded. Interventions that target the virus directly such as antivirals and antibodies were included, as well as interventions that target the immune response and interventions that help manage or prevent the severe and critical stages of the disease. The interventions included re-purposed drugs and NCEs.
- There was no information on endpoints of special interest.
- The study subjects were not relevant. Studies in healthy volunteers were excluded and studies solely in special patient populations (HIV, immunocompromised, cancer) were excluded as well.
- The sample size was too low (< 10 patients)
- The source was unavailable, withdrawn or retracted.
- The results were deemed unreliable because of inconsistencies or biologically implausible values
- The source was a secondary (duplicate) reference. This is an additional (secondary) source or publication of a study already flagged for inclusion in the database. All sources for a particular trial were evaluated for additional information of interest. Only secondary sources that report additional information were included.

As of February 1, 2022, a total of 251,260 sources have been identified since the start of the pandemic. Of these sources, 22,056 were manually reviewed for potential relevant clinical information on risk factors, treatments, and vaccines. A total of 1,941 were allocated to the treatment group of which 1,121 (492 RCTs and 629 RWS) were flagged for inclusion into the database. The detailed reasons for exclusion are shown in the figure below. Each source was given a priority for curation based on sample size and evaluated treatments. RCTs were always ranked with a higher priority than observational studies. Of the 492 sources reporting result of a RCT, 313 are in the database and 115 have been curated and are pending augmentation and final quality control (QC). Of the 629 sources reporting result of an observational study, 259 are in the database and 55 have been curated and are pending augmentation. The sources included in the database provide information on 543 controlled studies that evaluate treatments for COVID-19.

Figure S2: Number of sources reviewed and included or excluded from the COVID-19 database

COVID-19

sources identified

N = 251,260

Algorithm to flag sources that evaluate treatments and are not a review or meta-analysis

manual review

N= 22,056

Exclusion (N=820)

Secondary: 283

Study type: 78

No control: 227

Indication: 7

Intervention: 104

Subjects: 18

Endpoints: 53

Sample size: 38

Availability: 9

Reliability: 3

Categorized in

Risk factors: 690

prophylaxis: 93

vaccine: 230

biomarkers: 57

other: 19,045

Categorized in

treatment group

N = 1,941

Flagged for inclusion

N = 1,121

RCTs N = 492

In database: 313

Pending QC: 115

Pending curation: 64

RWS N = 629

In database: 259

Pending QC: 55

Pending curation: 315

Once a source was identified to contain relevant information, the study data from the source was extracted into the database. Apart from extracting raw data from the sources, the data was augmented and structured before inclusion into the database. This includes the standardization of endpoint names and definitions, normalization of endpoint units and all statistical calculations that are required to facilitate further analysis of the data. The structuring and augmentation produced a data base that was ready for further analysis with minimal user manipulation. Access to the COVID-19 outcomes database and detailed information on fields extracted into the database are available on request (<https://www.covidpharmacology.com/outcomes-database/>).

## Supplement B: Study Quality Factors - Risk of Bias

### Risk of bias for RCT

The risk of bias for randomised controlled trials (RCTs) as assessed by the Cochrane criteria and reported by <https://covid-nma.com/living_data/index.php> was used for the quality assessment of the RCTs. The studies were categorized into Tier 3 (high risk), Tier 2 (some concerns) and Tier 1 (low risk).

With respect to randomised-controlled trials, six were unavailable within our reference living network meta-analysis (NMA). The RCTs were distributed across the tiers as follows:

- Tier 1 (low risk): 13 studies
- Tier 2 (some concerns): 66 studies
- Tier 3 (High): 10 studies
- Not reported: 6 studies

### Risk of bias due to confounding in RWS

Risk of bias due to confounding is typical in real-world studies (RWS) and occurs if there is a factor that impacts subjects receiving treatment (such as disease severity) which also impacts outcome (such as higher mortality in patients with more severe disease). The COVID-19 database tracks several key variables that were used to determine the overall risk of bias due to confounding:

- Treatment rules: indication of whether the inclusion rules for control patients were similar to the treatment rules for treated patients in an observational study. Treatment rules were considered the same if there was an exact match of criteria that determined allocation of patients to the control and treated groups. They were similar if the target was a similar population. Differences were indicated as well.
- Balance: indication of whether the original cohorts (before matching) displayed a balance or imbalance for key variables; age, sex, disease severity, or comorbidities across the groups. If there was an imbalance, the variables were listed for which the imbalance is observed
- Confounding bias mitigation: analysis methodology used to mitigate risk of confounding bias in observational studies. Methods were a propensity score (PS) based matched cohort, Inverse Probability of Treatment Weighting (IPTW), Multivariable (MV) Analysis, matched cohort by other techniques, or none
- Confounding mitigation variables: listing of key variables that were considered or included in the analysis methodology to mitigate confounding bias. The variables considered were age, sex, race, disease severity, concomitant medications, comorbidities, and biomarkers

The studies were categorized into Tier 1, 2, 3 with regards to the risk of bias due to confounding. All studies started at Tier 3, the highest risk of bias due to confounding. Studies were moved to Tier 2 if they employed a bias mitigating analysis strategy of a PS matched cohort, IPTW, or MV, if they used similar rules to allocate patients to treated and control groups, or if they were well balanced with regards to key variables such as age, sex, disease severity, and comorbidities. The studies that advanced into Tier 2 because they employed a bias mitigating analysis strategy were advanced into Tier 1 if age, sex, disease severity and comorbidities were included in the matching or analysis strategy. The studies that moved into Tier 2 because they employed similar rules to allocate patients to treated and control groups were advanced to Tier 1 if they were well balanced with regards to key variables such as age, sex, disease severity, and comorbidities.

### Risk of immortal time bias in RWS

The figure below indicates important events that influence the risk of immortal time bias. In most studies, the index event (start of follow-up) is hospitalization whereas treatment was initiated up to several days after that. This results in a positive bias for the treatment effect on mortality, i.e. underestimation of odds-ratio for mortality, because by definition the patients in the treatment group could not have died before they received treatment (immortal time).

Figure S3: Important events that affect the risk of immortal time bias


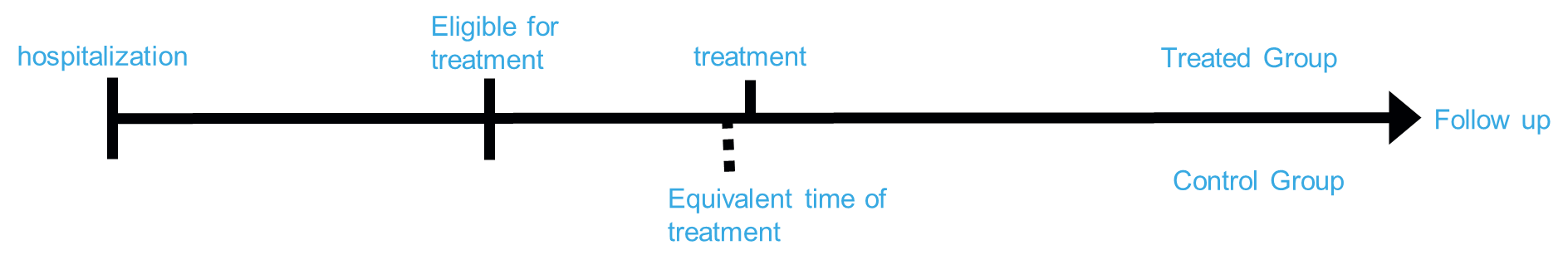


The risk of immortal time bias can be minimized by making sure that treatment is at or very close to the index event. This happens naturally when treatment is given close to hospitalization or to patients reaching a well-defined criteria for treatment eligibility. This can be achieved in the analysis by matching control patients at an equivalent time when treatment would have happened and making that the index event (time dependent matching).

The risk of immortal time bias can be mitigated by removing control patients with events in the “treatment window”, allocating patients with events in the treatment window randomly to each arm, or use treatment as a time-dependent covariate in the analysis. The COVID-19 database tracks several key variables that were used to determine the overall risk of bias due to immortal time:

- Time to treatment: time to treatment in the treated group from an event similar to the index event in control group (i.e. the time it would have taken controls to get from index to treatment). There was a descriptive indication of median or mean day to treatment, for example "median 1 day", or if all patients were treated within a certain time period, for example "within 2 days". If the time of the index in the control group was the same as that for treatment this was indicated with "0 days". This would occur if both groups were treated (i.e. active control), treatment occured at time of eligibility for study, or if a time dependent matching was used. If the time to treatment was likely to be short because the eligibility criteria for the controls were similar to the treatment criteria, and treatment happened almost immediately after this was reached, this was indicated as "probably short".
- Immortal time bias mitigation: methodology used to minimize the impact of immortal time bias. In addition to time dependent matching, studies used time-to-treatment as a covariate, or treatment as a time dependent variable. Some studies removed patients with events within the treatment window (grace period) or allocated patients with events within the treatment window randomly to control and treated arms.

The studies were categorized into Tier 1, 2, 3 with regards to the risk of bias due to immortal time. All studies started at Tier 3, the highest risk of bias due to immortal time. Studies were moved to Tier 1 if treatment occurred in all groups at index, if the mean/median time to treatment is less or equal to 1 day, if all patients were treated within 2 days, or if the analysis implemented a time-dependent matching. Studies were moved to Tier 2 if they used immortal time bias mitigation strategy that included treatment as a time dependent covariate, included time to treatment as a covariate, removed patients with events in the treatment window or allocated patients with events in the treatment window to groups randomly. Studies could also advance to Tier 2 if the time to treatment was judged to be short given trial design or treatment strategy.

Combination bias assessments were also evaluated and two scenarios were considered. In the first, overall Tier was determined by the worst of the two sub-scores (**Table S1**), and in the second, combination overall tier was determined by a combination of scores (**Table S2**).

Table S1. Worst Tier classification based on confounding and immortal time classification

| Worst Tier | confounding tier 1 | confounding tier 2 | confounding tier 3 |
| --- | --- | --- | --- |
| immortal Tier 1 | 1 | 2 | 3 |
| immortal Tier 2 | 2 | 2 | 3 |
| immortal Tier 3 | 3 | 3 | 3 |

Table S2. Combo Tier classification based on confounding and immortal time classification

| Combo Tier | confounding tier 1 | confounding tier 2 | confounding tier 3 |
| --- | --- | --- | --- |
| immortal Tier 1 | 1 | 1 | 2 |
| immortal Tier 2 | 1 | 2 | 3 |
| immortal Tier 3 | 2 | 3 | 3 |

### Risk of Bias in RWS - Results

Figure S4 and Figure S5 show the classification of the RWS into Tier 1, 2, or 3 for risk of bias due to confounding and risk of immortal time bias. The reason to move a study into each tier is provided. The classification strategy resulted in a reasonable balance of studies across the quality tiers.

Figure S4: Flowchart to determine quality “Tier” for risk of bias due to confounding with Tier 1 being the best (low risk) and Tier 3 the worst (high risk) for mortality analysis set. The number of studies is shown ().


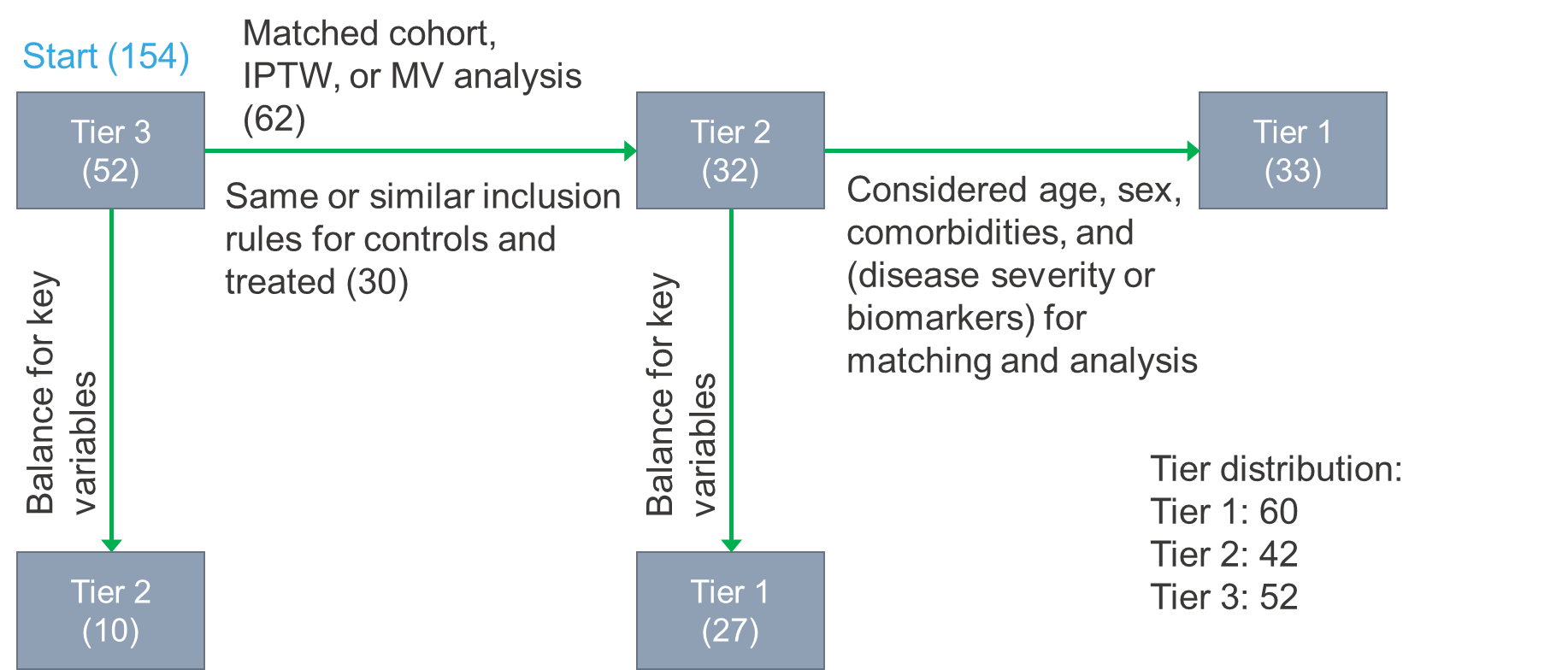


Figure S5: Flowchart to determine quality “Tier” for risk of bias due to immortal time with Tier 1 being the best (low risk) and Tier 3 the worst (high risk) for mortality analysis set. The number of studies is shown ().


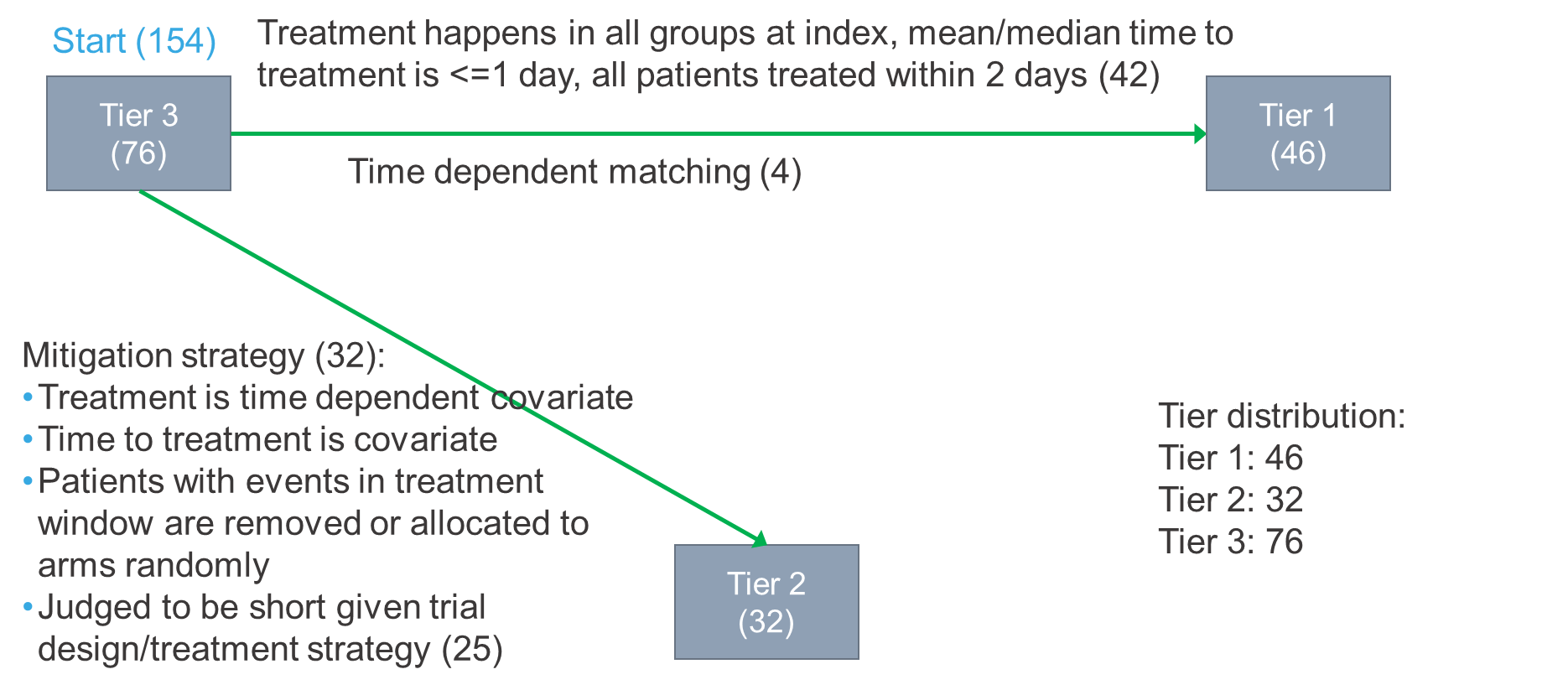


## Supplement C: Study extraction variables

The impact of population and study characteristics on the treatment effect was evaluated. The following variables were considered:

**Study characteristics:**

- Treatment categorized by drug and drug combinations
- Study type: randomised controlled trial (RCT) or real-world observational study (RWS).
- Time of endpoint observation
- Quality tier of assessment, as defined in **Supplement B: Study Quality Factors - Risk of Bias.**
- Bias control mechanisms employed by the authors of RWS to address the lack of randomisation. Categories were:
  - - propensity score matched sample
    - inverse probability of treatment weighting
    - multivariable analyses
    - no bias-mitigation
- Study region: the primary regions were North America, Europe, Africa, Asia, South America, Middle East
- Study publication status as a pre-print or peer reviewed journal article
- Time of first publication relative to start of pandemic. The time of first publication is taken from the CODEx COVID-19 source database marking when the information first became public. Dec 1, 2019 was defined as pandemic day 1.
- Time of study start relative to start of pandemic.
- Time of study midpoint relative to start of pandemic.
- Time of study publication relative to the time of key data readouts defined as the first of either RECOVERY or SOLIDARITY main publication. Categories are “before” and “after”
- Sample size
- Study blinding for RCTs
- Dose. The dose categories were low dose, medium dose, and high dose. The medium dose category was used when the doses covered the low and high groups or were unknown. Thresholds for low and high dose were set based on available data and clinical practice. Doses for different corticosteroids were converted to a methylprednisolone equivalent dose.

**Study population covariates:**

- Baseline disease severity – severity of disease as defined by the WHO scale.^28^ The following 5 level simplification was used given the availability of data across studies:
  - Mild: not hospitalized but unable to resume normal activities
  - Mild/moderate: hospitalized but not requiring oxygen support
  - Moderate: hospitalized requiring oxygen support via nasal cannula
  - Severe: hospitalized requiring high flow oxygen support or non-invasive ventilation
  - Critical: hospitalized requiring invasive ventilation
- Baseline disease severity score calculated as (1x%mild+2x%mild/moderate+3x%moderate+4x%severe+5x%critical)/100
- A simpler baseline disease severity scale was also considered collapsing some of the categories listed above:
  - Mild/moderate: not hospitalized or hospitalized but not requiring oxygen support
  - Moderate: hospitalized requiring oxygen support via nasal cannula
  - Severe/critical: hospitalized requiring high flow oxygen support, non-invasive ventilation, or invasive ventilation
- Imbalance in patient characteristics between the treated and control groups. Differences in the following characteristics known to impact mortality were evaluated:
  - Age – difference in average age of patients between active and control group
  - Sex – difference in percent of males between active and control groups
  - Disease severity – difference in percent of patients with critical disease between active and control groups. Other thresholds for severity were also evaluated such as percent with at least severe disease, or percent on oxygen (at least moderate disease)
  - Diabetes – difference in percent of patients with diabetes between active and control groups
  - Hypertension – difference in percent of patients with hypertension between active and control groups
  - Other co-morbidities will be included if more than 65% of studies include the information.

A select number of studies produce results by subgroup reflecting disease severity, each of which were included.

The table below shows the dose or dose regimen thresholds that were used to group each treatment for which there was information on different dose strengths into a low and high dose group. The classification was based on available data and clinical practice

Table S3. Classification of dose groups for each treatment

| **drug** | **Lower dose** | **Higher dose** |
| --- | --- | --- |
| tocilizumab | <=4 mg/kg; <=400 mg | >=6 mg/kg; >400 mg |
| hydroxychloroquine | <=400 mg/day | >=600 mg/day |
| convalescent plasma | Single dose (1 Unit) | Multiple doses (>=2 Units close together) |
| remdesivir | 5 day regimen | 10 day regimen |
| lopinavir/ritonavir | No dose range | |
| glucocorticoid | <=80 mg/day met eq. | >80 mg/day met eq. |
| azithromycin | No dose range | |

## Supplement D: Study details

### Included treatment classes

**Table S4** provides a listing of all therapies evaluated for inclusion. Therapeutic interventions 9 (ivermectin) and above were not considered eligible for analysis, since they had fewer than 5 RWS and 5 RCTs. Included studies are presented in Table 5**.**

Table S4: A list of therapeutic evaluations considered

| **No** | **Treatment** | **Therapeutic group** | **Mechanism** | **Number of patients** | | | **Number of studies** | | |
| --- | --- | --- | --- | --- | --- | --- | --- | --- | --- |
|  |  |  |  | **total** | **RWS** | **RCT** | **total** | **RWS** | **RCT** |
| 1 | tocilizumab | immunomodulator | anti-IL-6 | 45332 | 37170 | 8162 | 63 | 50 | 13 |
| 2 | glucocorticoid | glucocorticoid | glucocorticoid | 23541 | 15504 | 8037 | 48 | 38 | 10 |
| 3 | hydroxychloroquine | antimalarial | TLR-9 inhibitor | 52246 | 40392 | 11854 | 55 | 29 | 26 |
| 4 | convalescent plasma | antibody | plasma therapy | 29982 | 11016 | 18966 | 45 | 21 | 24 |
| 5 | remdesivir | antiviral | adenosine nucleotide | 119160 | 109495 | 9665 | 24 | 15 | 9 |
| 6 | hydroxychloroquine+ azithromycin | antimalarial+  antibiotic | TLR-9 inhibitor+  antibiotic | 16614 | 15462 | 1152 | 20 | 12 | 8 |
| 7 | lopinavir/ritonavir | antiviral | protease inhibitor | 10626 | 1199 | 9427 | 14 | 7 | 7 |
| 8 | azithromycin | antibiotic | antibiotic | 15364 | 6543 | 8821 | 11 | 5 | 6 |
| 9 | ivermectin | antiparasitic | anthelmintic | 5470 | 3305 | 2165 | 16 | 4 | 12 |
| 10 | favipiravir | antiviral | polymerase inhibitor | 3266 | 1611 | 1655 | 13 | 4 | 9 |
| 11 | anakinra | immunomodulator | anti-IL-1 | 1939 | 460 | 1479 | 7 | 4 | 3 |
| 12 | umifenovir | antiviral | fusion inhibitor | 934 | 658 | 276 | 6 | 3 | 3 |
| 13 | zinc | supplement | supplement | 1505 | 1174 | 331 | 5 | 2 | 3 |
| 14 | immunoglobulin | other | immunoglobulin | 747 | 358 | 389 | 6 | 2 | 4 |
| 15 | colchicine | anti-inflammatory | anti-inflammatory | 19436 | 178 | 19258 | 14 | 2 | 12 |
| 16 | aspirin | anticoagulant | anti-platelet | 15304 | 412 | 14892 | 2 | 1 | 1 |
| 17 | lopinavir/ritonavir+  hydroxychloroquine | antimalarial+  antiviral | protease inhibitor+  TLR-9 inhibitor | 736 | 357 | 379 | 2 | 1 | 1 |

Included studies

Table S5: A list of included studies

| **References** | **Study name** | **Registry name** | **Link** |
| --- | --- | --- | --- |
| Abd-Elsalam S, Ahmed OA, Mansour NO, Abdelaziz DH, Salama M, Fouad MHA, Soliman S, Naguib AM, Hantera MS, Ibrahim IS, Torky M, Dabbous HM, Ghafar MSAE, Abdul-Baki EA, Elhendawy M (2021). Remdesivir Efficacy in COVID-19 Treatment: A Randomized Controlled Trial. Am J Trop Med Hyg. | COVID 19 treatment | NCT04345419 | http://www.ncbi.nlm.nih.gov/pubmed/34649223 |
| Abd-Elsalam S, Esmail ES, Khalaf M, Abdo EF, Medhat MA, Abd El Ghafar MS, Ahmed OA, Soliman S, Serangawy GN, Alboraie M (2020). Hydroxychloroquine in the Treatment of COVID-19: A Multicenter Randomized Controlled Study. Am J Trop Med Hyg. | Abd-Elsalam S 2020 | NCT04353336 | http://www.ncbi.nlm.nih.gov/pubmed/32828135 |
| Abdulrahman A, Alsayed I, Almadhi M, Alarayed J, Mohammed SJ, Sharif AK, Alansari K, Alawadhi AI, Alqahtani M (2020). The efficacy and safety of hydroxychloroquine in COVID19 patients : a multicenter national retrospective cohort. medRxiv. | Abdulrahman A 2020 |  | http://doi.org/10.1101/2020.11.25.20234914 |
| Aboelsaad I, Ashmawy R, Mahrous D, Sharaf S, Aly S, Abdullatif S, Fakhry A, Hassan B, Khamis D, Aldakhs A, Kamal E (2021). Clinical outcomes and survival analysis of Remdesivir as a treatment option for moderate to severe COVID-19 patients.. Research Square. | Aboelsaad I 2021 |  | https://doi.org/10.21203/rs.3.rs-848909/v1 |
| Abolghasemi H, Eshghi P, Cheraghali AM, Imani Fooladi AA, Bolouki Moghaddam F, Imanizadeh S, Moeini Maleki M, Ranjkesh M, Rezapour M, Bahramifar A, Einollahi B, Hosseini MJ, Jafari NJ, Nikpouraghdam M, Sadri N, Tazik M, Sali S, Okati S, Askari E, Tabarsi P, Aslani J, Sharifipour E, Jarahzadeh MH, Khodakarim N, Salesi M, Jafari R, Shahverdi S (2020). Clinical efficacy of convalescent plasma for treatment of COVID-19 infections: Results of a multicenter clinical study. Transfus Apher Sci. | Abolghasemi H 2020 | IRCT20200325046860N1 | http://www.ncbi.nlm.nih.gov/pubmed/32694043 |
| Ader F, Peiffer-Smadja N, Poissy J, Bouscambert-Duchamp M, Belhadi D, Diallo A, Delmas C, Saillard J, Dechanet A, Mercier N, Dupont A, Alfaiate T, Lescure FX, Raffi F, Goehringer F, Kimmoun A, Jaureguiberry S, Reignier J, Nseir S, Danion F, Clere-Jehl R, Bouiller K, Navellou JC, Tolsma V, Cabie A, Dubost C, Courjon J, Leroy S, Mootien J, Gaci R, Mourvillier B, Faure E, Pourcher V, Gallien S, Launay O, Lacombe K, Lanoix J, Makinson A, Martin-Blondel G, Bouadma L, Botelho-Nevers E, Gagneux-Brunon A, Epaulard O, Piroth L, Wallet F, Richard JC, Reuter J, Staub T, Hites M, Noret M, Andrejak C, Peytavin G, Lina B, Costagliola D, Yazdanpanah Y, Burdet C, Mentre F (2021). Antiviral drugs in hospitalized patients with COVID-19 - the DisCoVeRy trial. medRxiv. | DISCOVERY | NCT04315948, EudraCT2020-000936-23 | http://doi.org/10.1101/2021.01.08.20248149 |
| Agarwal A, Mukherjee A, Kumar G, Chatterjee P, Bhatnagar T, Malhotra P, Latha B, Bundas S, Kumar V, Dosi R, Khambholja JK, De SR, Mesipogu RR, Srivastava S, Dube S, Chaudhary K, SS, Mattuvar KSA, Rajendran V, Sundararajaperumal A, Balamanikandan P, Maheswari RSU, Jayanthi R, Ragunanthanan S, Bhandari S, Singh A, Pal A, Handa A, Rankawat G, Kargirwar K, Regi J, Rathod D, Pathrose E, Bhutaka N, Patel MH, Verma RJ, Malukani K, Patel S, Thakur A, Joshi S, Kulkarni R, Suthar NN, Shah NM, Purohit HM, Shah CK, Patel MN, Shah S, Shah SH, Memon T, Beriwala VR, Jashnani K, Ezzy F, Agrawal S, Bhadade R, NAM, Madke T, Kavishwar V, Waghmare R, Valvi N, Chander BT, Sekhar AV, Maurya AK, Hemanth K, Nagamani K, Sudha K, Chandra TR, Rao KT, Vyshnavi J, Upadhyay R, Bahadur S, Pathak R, Seth S, Gupta R, Saxena R, Dwivedi P, Malik R, Chourasia D, Lalwani J, Sharma U, Marko J, Suri A, Kumar V, Kaushik R, Kodan P, Acharya BP, Gaur KK, Gupta A, Sachdeva P, Dogra S, Jindal A, John MJ, Dhanju AS, Khetrepal R, Sharma N, Kukar N, Kavita D, Kumar R, Mahajan R, Singh G, Kaur J, Singh RP, Bassi R, Parikh S, Shrivastav O, Shastri J, Desai M, Udupa S, Bafna VA, Barge V, Madane R, Yadav S, Mishra S, Bajpayee A, Garg MK, Bohra GK, Nag V, Anne PB, Nadeem M, Singh P, Niwas R, Khaire NS, Sharma R, Singh MP, Sachdeva N, Sachdev S, Hans R, Suri V, Yaddanapudi LN, Lakshmi P, Singh N, Bhushan D, Kumar N, Tambe M, Salvi S, Kadgi N, Sangle S, Nakate L, Joshi S, Karyakarte R, Goyanka S, Sharma N, Verma N, Das A, Bahl M, Wadhwa N, Bhat S, Deshmukh S, Wagh V, Kulkarni A, Yardi T, Kalgud RS, Reddy P, Yevoor K, Gajula P, Maleyur V, SM, Hn M, Gurtoo A, Sud R, Pahuja S, Prakash A, Gogoi P, Shukla S, Reddy DH, Chandra T, Pandey S, Maurya P, Wahid A, Kumar V, Upadhyay K, Bhatnagar N, Shah N, Shah M, Patel T, Jaiswal RM, Jain A, Sharma S, Rijhwani P, Gupta N, Patel TC, Solu MG, Patel J, Shah YR, Jarag M, Godbole V, Shah M, Raj R, Nagori I, Jha PR, Shah AD, Yeeli G, Jain A, Gill RK, Babu KS, Babu BS, Mohan A, Vengamma B, Sekhar KC, Damam S, Narsimhulu K, Aparna C, Baleswari G, KRR, Chandrasekhar P, Panjwani SJ, Akholkar PJ, Joshi KP, Shah PH, Barvaliya M, Baldi M, Yadav A, Gupta M, Rawat N, Chawda D, Natarajan M, Sintha M, Kumar DP, Rabbani F, Khadke VK, Patki D, Marathe S, DS C, Tadha V, Arora S, Gupta DK, Dua S, Chauhan N, Chahar AS, Mammen JJ, Kumar S, Daniel D, Singh R, Dhat V, Agarwal Y, Arora S, Pathak A, Purohit M, Sharma A, Sharma J, Madkaikar M, Joshi K, Yadav RM, Bhagwat S, Karnik ND, Gokhale YA, Naik L, Margam S, Das S, Turuk A, Kumar VS, Kanagasabai K, Sabarinathan R, Deshpande G, Sharma S, Gunjikar R, Shete A, Phagiwala D, Patil C, Shingade S, Jarande K, Kaushal H, Yadav P, Sapkal G, Abraham P (2020). Convalescent plasma in the management of moderate COVID-19 in India: An open-label parallel-arm phase II multicentre randomized controlled trial (PLACID Trial). medRxiv. | PLACID | CTRI/2020/04/024775 | http://doi.org/10.1101/2020.09.03.20187252 |
| Albani F, Fusina F, Giovannini A, Ferretti P, Granato A, Prezioso C, Divizia D, Sabaini A, Marri M, Malpetti E, Natalini G (2020). Impact of Azithromycin and/or Hydroxychloroquine on Hospital Mortality in COVID-19. J Clin Med 9: 2800. | Albani F 2020_HCQ/AZT |  | http://www.ncbi.nlm.nih.gov/pubmed/32872629 |
| Albani F, Fusina F, Granato E, Capotosto C, Ceracchi C, Gargaruti R, Santangelo G, Schiavone L, Taranto MS, Tosati C, Vavassori E, Natalini G (2020). Effect of corticosteroid treatment on 1376 hospitalized COVID-19 patients. A cohort study. medRxiv. | Albani F 2020_corticosteroids |  | http://doi.org/10.1101/2020.07.17.20155994 |
| Ali K, Azher T, Baqi M, Binnie A, Borgia S, Carrier FM, Cavayas YA, Chagnon N, Cheng MP, Conly J, Costiniuk C, Daley P, Daneman N, Douglas J, Downey C, Duan E, Duceppe E, Durand M, English S, Farjou G, Fera E, Fontela P, Fowler R, Fralick M, Geagea A, Grant J, Harrison LB, Havey T, Hoang H, Kelly LE, Keynan Y, Khwaja K, Klein G, Klein M, Kolan C, Kronfli N, Lamontagne F, Lau R, Fralick M, Lee TC, Lee N, Lim R, Longo S, Lostun A, MacIntyre E, Malhamé I, Mangof K, McGuinty M, Mergler S, Munan MP, Murthy S, O'Neil C, Ovakim D, Papenburg J, Parhar K, Parvathy SN, Patel C, Perez-Patrigeon S, Pinto R, Rajakumaran S, Rishu A, Roba-Oshin M, Rushton M, Saleem M, Salvadori M, Scherr K, Schwartz K, Semret M, Silverman M, Singh A, Sligl W, Smith S, Somayaji R, Tan DHS, Tobin S, Todd M, Tran TV, Tremblay A, Tsang J, Turgeon A, Vakil E, Weatherald J, Yansouni C, Zarychanski R (2022). Remdesivir for the treatment of patients in hospital with COVID-19 in Canada: a randomized controlled trial.. CMAJ. | CATCO | NCT04330690 | http://www.ncbi.nlm.nih.gov/pubmed/35045989 |
| Alotaibi M, Ali A, Bakhshwin D, Alatawi Y, Alotaibi S, Alhifany A, Alharthi B, Alharthi N, Alyazidi A, Alharthi Y, Alrafiah A (2021). Effectiveness and Safety of Favipiravir Compared to Hydroxychloroquine for Management of Covid-19: A Retrospective Study. Int J Gen Med 14: 5597-5606. | Alotaibi M 2021 |  | http://www.ncbi.nlm.nih.gov/pubmed/34548811 |
| Alqahtani M, Abdulrahman A, Almadani A, Alali SY, Zamrooni AMA, Hejab AH, Conroy RM, Wasif P, Atkin SL, Otoom S, Abduljalil M (2020). Randomized controlled trial of convalescent plasma therapy against standard therapy in patients with severe COVID-19 disease. medRxiv. | AlQahtani M 2020 | NCT04356534 | http://doi.org/10.1101/2020.11.02.20224303 |
| Alsayed HAH, Sharif-Askari FS, Sharif-Askari NS, Hussain AAS, Hamid Q, Halwani R (2021). Early administration of remdesivir to COVID-19 patients associates with higher recovery rate and lower need for ICU admission: A retrospective cohort study.. PLoS One 16: e0258643. | Alsayed HAH 2021 |  | http://www.ncbi.nlm.nih.gov/pubmed/34699552 |
| Alsharidah S, Ayed M, Ameen RM, Alhuraish F, Rouheldeen NA, Alshammari FR, Embaireeg A, Almelahi M, Adel M, Dawoud ME, Aljasmi MA, Alshammari N, Alsaeedi A, Al-Adsani W, Arian H, Awad H, Alenezi HA, Alzafiri A, Gouda EF, Almehanna M, Alqahtani S, Alshammari A, Askar MZ (2020). COVID-19 convalescent plasma treatment of moderate and severe cases of SARS-CoV-2 infection: A multicenter interventional study. Int J Infect Dis 103: 439-446. | MOHKuwait | NCT04474340 | http://www.ncbi.nlm.nih.gov/pubmed/33285283 |
| Altuntas F, Ata N, Yigenoglu TN, Basc? S, Dal MS, Korkmaz S, Namdaroglu S, Basturk A, Hac?bekiroglu T, Dogu MH, Berber ?, Dal K, K?n?k K, Haznedaroglu ?, Y?lmaz FM, K?l?c ?, Demircioglu S, Yosunkaya A, Erkurt MA, Turgut B, Caglayan M, Celik O (2020). Convalescent plasma therapy in patients with COVID-19. Transfus Apher Sci: 102955. | Altuntas F 2020 |  | http://www.ncbi.nlm.nih.gov/pubmed/33011076 |
| An MH, Kim MS, Park YK, Kim BO, Kang SH, Kimn WJ, Park SK, Park HW, Yang W, Jang J, Jang SW, Hwang TH (2020). Treatment Response to Hydroxychloroquine and Antibiotics for mild to moderate COVID-19: a retrospective cohort study from South Korea. medRxiv. | An MH 2020 |  | http://doi.org/10.1101/2020.07.04.20146548 |
| Angus DC, Derde L, Al-Beidh F, Annane D, Arabi Y, Beane A, van Bentum-Puijk W, Berry L, Bhimani Z, Bonten M, Bradbury C, Brunkhorst F, Buxton M, Buzgau A, Cheng AC, de Jong M, Detry M, Estcourt L, Fitzgerald M, Goossens H, Green C, Haniffa R, Higgins AM, Horvat C, Hullegie SJ, Kruger P, Lamontagne F, Lawler PR, Linstrum K, Litton E, Lorenzi E, Marshall J, McAuley D, McGlothin A, McGuinness S, McVerry B, Montgomery S, Mouncey P, Murthy S, Nichol A, Parke R, Parker J, Rowan K, Sanil A, Santos M, Saunders C, Seymour C, Turner A, van de Veerdonk F, Venkatesh B, Zarychanski R, Berry S, Lewis RJ, McArthur C, Webb SA, Gordon AC (2020). Effect of Hydrocortisone on Mortality and Organ Support in Patients With Severe COVID-19: The REMAP-CAP COVID-19 Corticosteroid Domain Randomized Clinical Trial. JAMA 324: 1317-1329. | REMAP-CAP_steroid | NCT02735707 | http://www.ncbi.nlm.nih.gov/pubmed/32876697 |
| Annie FH, Sirbu C, Frazier KR, Broce M, Lucas BD (2020). Hydroxychloroquine in hospitalized COVID-19 patients: Real world experience assessing mortality. Pharmacotherapy 40: 1072-1081. | Annie FH 2020_HCQ/AZT, Annie FH 2020_hydroxychloroquine |  | http://www.ncbi.nlm.nih.gov/pubmed/33044019 |
| Arabi YM, Gordon AC, Derde LPG, Nichol AD, Murthy S, Beidh FA, Annane D, Swaidan LA, Beane A, Beasley R, Berry LR, Bhimani ZB, Bonten MJM, Bradbury CA, Brunkhorst FM, Buxton M, Buzgau A, Cheng A, Jong MD, Detry MA, Duffy EJ, Estcourt LJ, Fitzgerald M, Fowler R, Girard TD, Goligher EC, Goossens H, Haniffa R, Higgins AM, Hills TE, Horvat CM, Huang DT, King AJ, Lamontagne F, Lawler PR, Lewis R, Linstrum K, Litton E, Lorenzi E, Malakouti S, McAuley DF, McGlothlin A,Mcguinness S, McVerry BJ, Montgomery SK, Morpeth SC, Mouncey PR, Orr K, Parke R, Parker JC, Patanwala AE, Rowan KM, Santos MS, Saunders CT, Seymour CW, Hari MS, Tong SYC, Turgeon AF, Turner AM, Veerdonk FLVD, Zarychanski R, Green C, Berry S, Marshall JC, McArthur C, Angus DC, Webb SA (2021). Lopinavir-ritonavir and hydroxychloroquine for critically ill patients with COVID-19: REMAP-CAP randomized controlled trial. Intensive Care Med 47: 867-886. | REMAP-CAP_antiviral | NCT02735707, EudraCT2015-002340-14 | http://www.ncbi.nlm.nih.gov/pubmed/34251506 |
| Arch BN, Kovacs D, Scott JT, Jones AP, Harrison EM, Rosala-Hallas A, Gamble CG, Openshaw PJM, Baillie JK, Semple MG (2021). Evaluation of the effectiveness of remdesivir in treating severe COVID-19 using data from the ISARIC WHO Clinical Characterisation Protocol UK: a prospective, national cohort study. medRxiv. | Arch BN 2021 |  | https://doi.org/10.1101/2021.06.18.21259072 |
| Arshad S, Kilgore P, Chaudhry ZS, Jacobsen G, Wang DD, Huitsing K, Brar I, Alangaden GJ, Ramesh MS, McKinnon JE, O'Neill W, Zervos M, Nauriyal V, Hamed AA, Nadeem O, Swiderek J, Godfrey A, Jennings J, Gray JG, Ackerman AM, Lezotte J, Ruhala J, Fadel R, Vahia A, Gudipati S, Parraga T, Shallal A, Maki G, Tariq Z, Suleyman G, Yared N, Herc E, Williams J, Lanfranco OA, Bhargava P, Reyes K, Chen A (2020). Treatment with Hydroxychloroquine, Azithromycin, and Combination in Patients Hospitalized with COVID-19. Int J Infect Dis. | Arshad S 2020 |  | http://www.ncbi.nlm.nih.gov/pubmed/32623082 |
| Ates i, Erden A, Guven SC, Gurler EK, Caglayan A, Gucbey O, Apaydin H, Sahiner ES, Kucuk H, Varan O, Omma A, Kucuksahin O (2021). Should timing be considered before abandoning convalescent plasma in covid-19? Results from the Turkish experience.. Transfus Apher Sci 60: 103238. | Ates i 2021 |  | http://www.ncbi.nlm.nih.gov/pubmed/34412949 |
| Avendano-Sola C, Ramos-Martinez A, Munez-Rubio E, Ruiz-Antoran B, Malo DMR, Torres F, Fernandez-Cruz A, Callejas-Diaz A, Calderon J, Payares C, Salcedo I, Romera I, Lora-Tamayo J, Mancheno-Losa M, Paciello ML, Villegas C, Estrada V, Saez-Serrano I, Porras-Leal ML, Jarilla-Fernandez MC, Pano-Pardo JR, Moreno-Chulilla JA, Arrieta-Aldea I, Bosch A, Belhassen-Garcia M, Lopez-Villar O, Ramos-Garrido A, Blanco L, Madrigal-Sanchez ME, Contreras E, Muniz-Diaz E, Domingo-Morera JM, Casas-Flecha I, Perez-Olmeda M, Garcia Perez-J, Alcami J, Bueno JL, Duarte RF (2020). Convalescent Plasma for COVID-19: A multicenter, randomized clinical trial. medRxiv. | ConPlas-19 | NCT04345523 | http://doi.org/10.1101/2020.08.26.20182444 |
| Bajpai M, Kumar S, Maheshwari A, Chhabra K, Kale P, Gupta A, Narayanan A, Gupta E, Trehanpati N, Bihari C, Agarwal R, Gupta K, Gupta UK, Bhardwaj A, Kumar G, Islam M, Singh R, Yadav P, Maiwall R, Sarin SK (2020). Efficacy of Convalescent Plasma Therapy compared to Fresh Frozen Plasma in Severely ill COVID-19 Patients: A Pilot Randomized Controlled Trial. medRxiv. | ILBS-COVID-02 | NCT04346446 | http://doi.org/10.1101/2020.10.25.20219337 |
| Bar KJ, Shaw PA, Choi GH, Aqui N, Fesnak A, Yang JB, Soto-Calderon H, Grajales L, Starr J, Andronov M, Mastellone M, Amonu C, Feret G, DeMarshall M, Buchanan M, Caturla M, Gordon J, Wanicur A, Monroy MA, Mampe F, Lindemuth E, Gouma S, Mullin AM, Barilla H, Pronina A, Irwin L, Thomas R, Eichinger RA, Demuth F, Luning Prak ET, Pascual JL, Short WR, Elovitz MA, Baron J, Meyer NJ, Degnan KO, Frank I, Hensley SE, Siegel DL, Tebas P (2021). A randomized controlled study of convalescent plasma for individuals hospitalized with COVID-19 pneumonia.. J Clin Invest 131: e155114. | PennCCP-02 | NCT04397757 | http://www.ncbi.nlm.nih.gov/pubmed/34788233 |
| Barratt-Due A, Olsen IC, Nezvalova-Henriksen K, Kasine T, Lund-Johansen F, Hoel H, Holten AR, Tveita A, Mathiessen A, Haugli M, Eiken R, Kildal AB, Berg Å, Johannessen A, Heggelund L, Dahl TB, Skara KH, Mielnik P, Kieu Le LA, Thoresen L, Ernst G, Lihaug Hoff DA, Skudal H, Kittang BR, Olsen RB, Tholin B, Ystrom CM, Skei NV, Tran T, Dudman S, Andersen JT, Hannula R, Dalgard O, Finbraten AK, Tonby K, Blomberg B, Aballi S, Fladeby C, Steffensen A, Müller F, Dyrhol-Riise AM, Troseid M, Aukrust P (2021). Evaluation of the Effects of Remdesivir and Hydroxychloroquine on Viral Clearance in COVID-19 : A Randomized Trial.. Ann Intern Med. | NOR-Solidarity_hydroxycloroquine, NOR-Solidarity_remdesivir | NCT04321616 | http://www.ncbi.nlm.nih.gov/pubmed/34251903 |
| Bartoletti M, Marconi L, Scudeller L, Pancaldi L, Tedeschi S, Giannella M, Rinaldi M, Bussini L, Valentini I, Ferravante AF, Potalivo A, Marchionni E, Fornaro G, Pascale R, Pasquini Z, Puoti M, Merli M, Barchiesi F, Volpato F, Rubin A, Saracino A, Tonetti T, Gaibani P, Ranieri VM, Viale P, Cristini F (2020). Efficacy of corticosteroid treatment for hospitalized patients with severe COVID-19: a multicenter study. Clin Microbiol Infect. | PREDICO |  | http://www.ncbi.nlm.nih.gov/pubmed/32971254 |
| Begin P, Callum J, Jamula EJ, Cook R, Heddle NM, Tinmouth A, Zeller MP, Beaudoin BG, Amorim L, Bazin R, Cadogan LK, Carl R, Chassé M, Cushing M, Daneman N, Devine DV, Dumaresq J, Ferguson D, Gabe C, Glesby MJ, Li N, Liu Y, Mcgeer A, Robitaille N, Sachais BS, Scales DC, Schwartz L, Shehata N, Turgeon A, Wood H, Zarychanski R, Finzi A, Arnold DM (2021). Convalescent plasma for hospitalized patients with COVID-19 and the effect of plasma antibodies: a randomized controlled, open-label trial. medRxiv. | CONCOR-1 | NCT04348656 | https://www.medrxiv.org/content/10.1101/2021.06.29.21259427v1 |
| Beigel JH, Tomashek KM, Dodd LE, Mehta AK, Zingman BS, Kalil AC, Hohmann E, Chu HY, Luetkemeyer A, Kline S, de Castilla DL, Finberg RW, Dierberg K, Tapson V, Hsieh V, Patterson TF, Paredes R, Sweeney DA, Short WR, Touloumi G, Lye DC, Ohmagari N, Oh MD, Palacios GMR, Benfield T, Fatkenheuer G, Kortepeter MG, Atmar RL, Creech CB, Lundgren J, Babiker AG, Pett S, Neaton JD, Burgess TH, Bonnett T, Green M, Makowski M, Osinusi A, Nayak S, Lane HC (2020). Remdesivir for the Treatment of Covid-19 - Preliminary Report. N Engl J Med. | ACTT-1 | NCT04280705 | https://pubmed.ncbi.nlm.nih.gov/32445440 |
| Bennett-Guerrero E, Romeiser JL, Talbot LR, Ahmed T, Mamone LJ, Singh SM, Hearing JC, Salman H, Holiprosad DD, Freedenberg AT, Carter JA, Browne NJ, Cosgrove ME, Shevik ME, Generale LM, Andrew MA, Nachman S, Fries BC (2021). Severe Acute Respiratory Syndrome Coronavirus 2 Convalescent Plasma Versus Standard Plasma in Coronavirus Disease 2019 Infected Hospitalized Patients in New York: A Double-Blind Randomized Trial. Crit Care Med. | SBU-COVID19-ConvalescentPlasma | NCT04344535 | http://www.ncbi.nlm.nih.gov/pubmed/33870923 |
| Biran N, Ip A, Ahn J, Go RC, Wang S, Mathura S, Sinclaire BA, Bednarz U, Marafelias M, Hansen E, Siegel DS, Goy AH, Pecora AL, Sawczuk IS, Koniaris LS, Simwenyi M, Varga DW, Tank LK, Stein AA, Allusson V, Lin GS, Oser WF, Tuma RA, Reichman J, Brusco L Jr, Carpenter KL, Costanzo EJ, Vivona V, Goldberg SL (2020). Tocilizumab among patients with COVID-19 in the intensive care unit: a multicentre observational study. Lancet Rheumatol 2: e603-e612. | Biran N 2020 | NCT04347993 | http://www.ncbi.nlm.nih.gov/pubmed/32838323 |
| Briggs N, Gormally MV, Li F, Browning SL, Treggiari MM, Morrison A, Laurent-Rolle M, Deng Y, Hendrickson JE, Tormey CA, Desruisseaux MS (2021). Early but not late convalescent plasma is associated with better survival in moderate-to-severe COVID-19.. PLoS One 16: e0254453. | Briggs N 2021_early treatment, Briggs N 2021_late treatment |  | http://www.ncbi.nlm.nih.gov/pubmed/34320004 |
| Budhiraja S, Dewan A, Aggarwal R, Singh O, Juneja D, Pathak S, Singh YP, Gupta A, Rai R, Indrayan A, Jha V, Naithani R (2021). Effectiveness of convalescent plasma in Indian patients with COVID-19. Blood Cells Mol Dis 88: 102548. | Budhiraja S 2021 |  | http://www.ncbi.nlm.nih.gov/pubmed/33621948 |
| Campochiaro C, Della-Torre E, Cavalli G, De Luca G, Ripa M, Boffini N, Tomelleri A, Baldissera E, Rovere-Querini P, Ruggeri A, Monti G, De Cobelli F, Zangrillo A, Tresoldi M, Castagna A, Dagna L (2020). Efficacy and safety of tocilizumab in severe COVID-19 patients: a single-centre retrospective cohort study. Eur J Intern Med 76: 43-49. | COVID-BioB_tocilicumab | NCT04318366 | http://www.ncbi.nlm.nih.gov/pubmed/32482597 |
| Canziani LM, Trovati S, Brunetta E, Testa A, De Santis M, Bombardieri E, Guidelli G, Albano G, Folci M, Squadroni M, Beretta GD, Ciccarelli M, Castoldi M, Lleo A, Aghemo A, Vernile L, Malesci A, Omodei P, Angelini C, Badalamenti S, Cecconi M, Cremonesi A, Selmi C (2020). Interleukin-6 receptor blocking with intravenous tocilizumab in COVID-19 severe acute respiratory distress syndrome: A retrospective case-control survival analysis of 128 patients. J Autoimmun: 102511. | Canziani LM 2020 |  | http://www.ncbi.nlm.nih.gov/pubmed/32713677 |
| Cao B, Wang Y, Wen D, Liu W, Wang J, Fan G, Ruan L, Song B, Cai Y, Wei M, Li X, Xia J, Chen N, Xiang J, Yu T, Bai T, Xie X, Zhang L, Li C, Yuan Y, Chen H, Li H, Huang H, Tu S, Gong F, Liu Y, We Y, Dong C, Zhou F, Gu X, Xu J, Liu Z, Zhang Y, Li H, Shang L, Wang K, Li K, Zhou X, Dong X, Qu Z, Lu S, Hu X, Ruan S, Luo S, Wu J, Peng L, Cheng F, Pan L, Zou J, Jia C, Wang J1, Liu X1, Wang S, Wu X, Ge Q, He J, Zhan H, Qiu F, Guo L, Huang C, Jaki T, Hayden FG, Horby PW, Zhang D, Wang C (2020). A Trial of Lopinavir-Ritonavir in Adults Hospitalized with Severe Covid-19. N Engl J Med 382: 1787-1799. | LOTUS China | ChiCTR2000029308 | http://www.ncbi.nlm.nih.gov/pubmed/32187464 |
| Capra R, Rossi ND, Mattioli F, Romanelli G, Scarpazza C, Sormani MP, Cossi S (2020). Impact of Low Dose Tocilizumab on Mortality Rate in Patients With COVID-19 Related Pneumonia. Eur J Intern Med 76: 31-35. | Capra R 2020 |  | https://pubmed.ncbi.nlm.nih.gov/32405160 |
| Cardona-Pascual I, Berlana D, Martinez-Valle F, Campany-Herrero D, Montoro-Ronsano JB (2021). Effect of tocilizumab versus standard of care in adults hospitalized with moderate-severe COVID-19 pneumonia. Med Clin (Barc) S0025-7753: 00210-4. | Cardona-Pascual I 2021 |  | http://www.ncbi.nlm.nih.gov/pubmed/34147248 |
| Carvalho V, Turon R, Goncalves B, Ceotto VF, Kurtz P, Righy C (2020). Effects of Tocilizumab in Critically Ill Patients With COVID-19: A Quasi-Experimental Study. medRxiv. | Carvalho V 2020 |  | http://doi.org/10.1101/2020.07.13.20149328 |
| Castelnuovo AD, Costanzo S, Antinori A, Berselli N, Blandi L, Bruno R, Cauda R, Guaraldi G, Menicanti L, My I, Parruti G, Patti G, Perlini S, Santilli F, Signorelli C, Spinoni E, Stefanini GG, Vergori A, Ageno W, Agodi A, Aiello L, Agostoni P, Moghazi SA, Astuto M, Aucella F, Barbieri G, Bartoloni A, Bonaccio M, Bonfanti P, Cacciatore F, Caiano L, Cannata F, Carrozzi L, Cascio A, Ciccullo A, Cingolani A, Cipollone F, Colomba C, Crosta F, Pra CD, Danzi GB, D'Ardes D, Donati KG, Giacomo PD, Gennaro FD, Tano GD, D'Offizi G, Filippini T, Fusco FM, Gentile I, Gialluisi A, Gini G, Grandone E, Grisafi L, Guarnieri G, Lamonica S, Landi F, Leone A, Maccagni G, Maccarella S, Madaro A, Mapelli M, Maragna R, Marra L, Maresca G, Marotta C, Mastroianni F, Mazzitelli M, Mengozzi A, Menichetti F, Meschiari M, Minutolo F, Montineri A, Mussinelli R, Mussini C, Musso M, Odone A, Olivieri M, Pasi E, Petri F, Pinchera B, Pivato CA, Poletti V, Ravaglia C, Rinaldi M, Rognoni A, Rossato M, Rossi I, Rossi M, Sabena A, Salinaro F, Sangiovanni V, Sanrocco C, Scorzolini L, Sgariglia R, Simeone PG, Spinicci M, Trecarichi EM, Venezia A, Veronesi G, Vettor R, Vianello A, Vinceti M, Vocciante L, De Caterina R, Iacoviello L (2020). Use of hydroxychloroquine in hospitalised COVID-19 patients is associated with reduced mortality: Findings from the observational multicentre Italian CORIST study. Eur J Intern Med: 30335-6. | CORIST |  | http://www.ncbi.nlm.nih.gov/pubmed/32859477 |
| Catteau L, Dauby N, Montourcy M, Bottieau E, Hautekiet J, Goetghebeur E, van Ierssel S, Duysburgh E, Van Oyen H, Wyndham-Thomas C, Van Beckhoven D (2020). Low-dose Hydroxychloroquine Therapy and Mortality in Hospitalized Patients with COVID-19: A Nationwide Observational Study of 8075 Participants. Int J Antimicrob Agents 56: 106144. | Catteau L 2020 |  | http://www.ncbi.nlm.nih.gov/pubmed/32853673 |
| Cavalcanti AB, Zampieri FG, Rosa RG, Azevedo LCP, Veiga VC, Avezum A, Damiani LP, Marcadenti A, Kawano-Dourado L, Lisboa T, Junqueira DLM, de Barros E Silva PGM, Tramujas L, Abreu-Silva EO, Laranjeira LN, Soares AT, Echenique LS, Pereira AJ, Freitas FGR, Gebara OCE, Dantas VCS, Furtado RHM, Milan EP, Golin NA, Cardoso FF, Maia IS, Hoffmann Filho CR, Kormann APM, Amazonas RB, Bocchi de Oliveira MF, Serpa-Neto A, Falavigna M, Lopes RD, Machado FR, Berwanger O (2020). Hydroxychloroquine with or without Azithromycin in Mild-to-Moderate Covid-19. N Engl J Med. | Coalition-I | NCT04322123 | http://www.ncbi.nlm.nih.gov/pubmed/32706953 |
| Chauhan L, Pattee J, Ford J, Thomas C, Lesteberg K, Richards E, Bernas CA, Loi M, Dumont L, Annen K, Berg M, Zirbes M, Knight V, Miller A, Jenkins TC, Bennett TD, Monkowski D, Boxer RS, Beckham JD (2021). A Multi-center, Prospective, Observational-cohort controlled study of Clinical Outcomes following COVID-19 Convalescent plasma therapy in hospitalized COVID-19 patients.. Clin Infect Dis. | Chauhan L 2021 |  | http://www.ncbi.nlm.nih.gov/pubmed/34549274 |
| Chen C, Lin Y, Chen T, Tseng T, Wong H, Kuo C, Lin W, Huang S, Wang W, Liao J, Liao C, Hung Y, Lin T, Chang T, Hsiao C, Huang Y, Chung W, Cheng C, Cheng S (2020). A Multicenter, randomized, open-label, controlled trial to evaluate the efficacy and tolerability of hydroxychloroquine and a retrospective study in adult patients with mild to moderate Coronavirus disease 2019 (COVID-19). medRxiv. | Chen C 2020b | NCT04384380 | https://doi.org/10.1101/2020.07.08.20148841 |
| Chen H, Xie J, Su N, Wang J, Sun Q, Li S, Jin J, Zhou J, Mo M, Wei Y, Chao Y, Hu W, Du B, Qiu H (2020). Corticosteroid therapy is associated with improved outcome in critically ill COVID-19 patients with hyperinflammatory phenotype. Chest S0012-3692: 35352-6. | Chen H 2020 |  | http://www.ncbi.nlm.nih.gov/pubmed/33316235 |
| Chen J, Liu D, Liu L, Liu P, Xu Q, Xia L, Ling Y, Huang D, Song S, Zhang D, Qian Z, Li T, Shen Y, Lu H (2020). A pilot study of hydroxychloroquine in treatment of patients with common coronavirus disease-19 (COVID-19). Zhejiang Da Xue Xue Bao Yi Xue Ban 49: 215-219. | HC-COVID-19 | NCT04261517 | https://www.ncbi.nlm.nih.gov/pubmed/32391667?dopt=Citation |
| Chen L, Zhang ZY, Fu JG, Feng ZP, Zhang SZ, Han QY, Zhang XB, Xiao X, Chen HM, Liu LL, Chen XL, Lan YP, Zhong DJ, Hu L, Wang JH, Yu XH, She DY, Zhu YH, Yin ZY (2020). Efficacy and safety of chloroquine or hydroxychloroquine in moderate type of COVID-19: a prospective open-label randomized controlled study. medRxiv. | Chen L 2020 | ChiCTR2000030054 | http://doi.org/10.1101/2020.06.19.20136093 |
| Chen Q, Song Y, Wang L, Zhang Y, Han L, Liu J, Yang M, Ma J, Wang T (2020). Corticosteroids treatment in severe patients with COVID-19: a propensity score matching study. Expert Rev Respir Med. | Chen Q 2020 |  | http://www.ncbi.nlm.nih.gov/pubmed/33249945 |
| Chronic Obstructive Pulmonary Disease Trial Network, Denmark (2021). Proactive Protection With Azithromycin and Hydroxychloroquine in Hospitalized Patients With COVID: A Randomized, Placebo-controlled Double-blinded Trial Evaluating Treatment With Azithromycin and Hydroxychloroquine to Patients With COVID-19. clintrials.gov. | ProPAC-COVID | NCT04322396, EudraCT2020-001198-55 | http://clinicaltrials.gov/show/NCT04322396 |
| Colaneri M, Bogliolo L, Valsecchi P, Sacchi P, Zuccaro V, Brandolino F, Montecucco C, Mojoli F, Giusti EM, Bruno R, The Covid Irccs San Matteo Pavia Task Force NA (2020). Tocilizumab for Treatment of Severe COVID-19 Patients: Preliminary Results from SMAtteo COvid19 REgistry (SMACORE).. Microorganisms 8: E695. | SMACORE |  | http://www.ncbi.nlm.nih.gov/pubmed/32397399 |
| Corral-Gudino L, Bahamonde A, Arnaiz-Revillas F, Gomez-Barquero J, Abadia-Otero J, Garcia-Ibarbia C, Mora V, Cerezo-Hernandez A, Hernandez JL, Lopez-Muniz G, Hernandez-Blanco F, Cifrian JM, Olmos JM, Carrascosa M, Nieto L, Farinas MC, Riancho JA (2021). Methylprednisolone in adults hospitalized with COVID-19 pneumonia : An open-label randomized trial (GLUCOCOVID). Wien Klin Wochenschr: 1-9. | GLUCOCOVID | EudraCT2020-001934-37 | http://www.ncbi.nlm.nih.gov/pubmed/33534047 |
| Cusacovich I, Aparisi A, Marcos M, Ybarra-Falcon C, Iglesias-Echevarria C, Lopez-Veloso M, Barraza-Vengoechea J, Duenas C, Martinez SAJ, Rodriguez-Alonso B, Martin-Oterino JA, Montero-Baladia M, Moralejo L, Andaluz-Ojeda D, Gonzalez-Fuentes R (2020). Corticosteroid pulses for hospitalized patients with COVID-19: Effects on mortality and in-hospital stay. medRxiv. | Cusacovich I 2020 |  | http://doi.org/10.1101/2020.09.30.20204719 |
| De Rossi N, Scarpazza C, Filippini C, Cordioli C, Rasia S, Mancinelli CR, Rizzoni D, Romanelli G, Cossi S, Vettoretto N, Bove S, Manfredini S, Beindorf EA, Mosca C, Scipione V, Flamminio G, Albini EA, Giansiracusa P, Capra R (2020). Early use of low dose tocilizumab in patients with COVID-19: A retrospective cohort study with a complete follow-up. EClinicalMedicine. | De Rossi N 2020 |  | http://www.ncbi.nlm.nih.gov/pubmed/32838235 |
| De Santis GC, Oliveira LC, Garibaldi PMM, Almado CEL, Croda J, Arcanjo GGA, Oliveira EAF, Tonacio AC, Langhi DM, Bordin JO, Gilio RN, Palma LC, Santos EV, Haddad SK, Prado BPA, Pontelli MC, Gomes R, Miranda CH, Martins MA, Covas DT, Arruda E, Fonseca BAL, Calado RT (2022). High-Dose Convalescent Plasma for Treatment of Severe COVID-19.. Emerg Infect Dis 28: 548-555. | De Santis GC 2022 |  | http://www.ncbi.nlm.nih.gov/pubmed/35081022 |
| Dequin PF, Heming N, Meziani F, Plantefeve G, Voiriot G, Badie J, Francois B, Aubron C, Ricard JD, Ehrmann S, Jouan Y, Guillon A, Leclerc M, Coffre C, Bourgoin H, Lengelle C, Caille-Fenerol C, Tavernier E, Zohar S, Giraudeau B, Annane D, Le Gouge A (2020). Effect of Hydrocortisone on 21-Day Mortality or Respiratory Support Among Critically Ill Patients With COVID-19: A Randomized Clinical Trial. JAMA. | CAPE COVID | NCT02517489 | http://www.ncbi.nlm.nih.gov/pubmed/32876689 |
| Derde LPG, Gordon AC, Mouncey PR, Al-Beidh F, Rowan KM, Nichol AD, Arabi YM, Annane D, Beane A, Beasley R, Bhimani Z, Bonten MJM, Bradbury CA, Brunkhorst FM, Buzgau A, Buxton M, Cheng AC, Cooper N, Cove M, Cremer OL, Detry MA, Duffy EJ, Estcourt LJ, Fitzgerald M, Galea J, Goossens H, Haniffa R, Hills TH, Ichihara N, King A, Lamontagne F, Lawler PR, Leavis HL, Lewis RJ, Linstrum KM, Litton E, Marshall JC, Mayr FB, McAuley D, McGlothlin A, McGuinness SP, McVerry BJ, Montgomery SK, Morpeth SC, Murthy S, Netea MG, Ogungbenro K, Orr K, Parke RL, Parker JC, Patanwala AE, Pettila V, Reyes LF, Sanil A, Saito H, Santos MS, Saunders CT, Saxena M, Seymour CW, Shankar-Hari M, Sligl WI, Turgeon AF, Turner AM, Tong S, Vaara S, Youngstein T, Zarychanski R, Green C, Higgins AM, McArthur CJ, Berry LR, Lorenzi E, Berry S, Webb SA, Angus DC, van de Veerdonk FL (2021). Effectiveness of Tocilizumab, Sarilumab, and Anakinra for critically ill patients with COVID-19 The REMAP-CAP COVID-19 Immune Modulation Therapy Domain Randomized Clinical Trial. medRxiv. | REMAP-CAP_Immunomodulator | NCT02735707 | https://doi.org/10.1101/2021.06.18.21259133 |
| Ding C, Feng X, Chen Y, Yuan J, Yi P, Li Y, Ni Q, Zou R, Li X, Sheng J, Li L, Xu K (2020). Effect of Corticosteroid Therapy on the Duration of SARS-CoV-2 Clearance in Patients with Mild COVID-19: A Retrospective Cohort Study. Infect Dis Ther 9: 943-952. | Ding C 2020 |  | http://www.ncbi.nlm.nih.gov/pubmed/32986226 |
| Dubee V, Roy PM, Vielle B, Parot-Schinkel E, Blanchet O, Darsonval A, Lefeuvre C, Abbara C, Boucher S, Devaud E, Robineau O, Rispal P, Guimard T, d’Anglejean E, Diamantis S, Custaud MA, Pellier I, Mercat A (2021). Hydroxychloroquine in mild-to-moderate COVID-19: a placebo-controlled double blind trial. Clin Microbiol Infect. | HYCOVID | NCT04325893, EudraCT2020-001271-33 | http://www.ncbi.nlm.nih.gov/pubmed/33813110 |
| Duke University (2020). Pragmatic Factorial Trial of Hydroxychloroquine, Azithromycin, or Both for Treatment of Severe SARS-CoV-2 Infection. clintrials.gov. | PRO00105339 | NCT04335552 | http://clinicaltrials.gov/show/NCT04335552 |
| Edalatifard M, Akhtari M, Salehi M, Naderi Z, Jamshidi A, Mostafaei S, Najafizadeh SR, Farhadi E, Jalili N, Esfahani M, Rahimi B, Kazemzadeh H, Mahmoodi Aliabadi M, Ghazanfari T, Satarian MR, Ebrahimi Louyeh H, Raeeskarami SR, Jamali Moghadam Siahkali S, Khajavirad N, Mahmoudi M, Rostamian A (2020). Intravenous methylprednisolone pulse as a treatment for hospitalised severe COVID-19 patients: results from a randomised controlled clinical trial. Eur Respir J: 2002808. | Edalatifard M 2020 | IRCT20200404046947N1 | http://www.ncbi.nlm.nih.gov/pubmed/32943404 |
| Eimer J, Vesterbacka J, Svensson AK, Stojanovic B, Wagrell C, Sonnerborg A, Nowak P (2020). Tocilizumab shortens time on mechanical ventilation and length of hospital stay in patients with severe COVID-19: a retrospective cohort study. J Intern Med. | Eimer J 2020 |  | http://www.ncbi.nlm.nih.gov/pubmed/32744399 |
| Estcourt LJ (2021). Convalescent Plasma in Critically ill Patients with COVID-19. medRxiv. | REMAP-CAP_Immunoglobulin | NCT02735707, EudraCT2015-002340-14 | https://doi.org/10.1101/2021.06.11.21258760 |
| Fernandez Cruz A, Ruiz-Antoran B, Munoz Gomez A, Sancho Lopez A, Mills Sanchez P, Centeno Soto GA, Blanco Alonso S, Javaloyes Garachana L, Galan Gomez A, Valencia Alijo A, Gomez Irusta J, Payares-Herrera C, Morras Torre I, Sanchez Chica E, Delgado Tellez de Cepeda L, Callejas Diaz A, Ramos Martinez A, Munez Rubio E, Avendano-Sola C (2020). A Retrospective Controlled Cohort Study of the Impact of Glucocorticoid Treatment in SARS-CoV-2 Infection Mortality. Antimicrob Agents Chemother. | Fernandez Cruz A 2020 | EUPAS34753 | http://www.ncbi.nlm.nih.gov/pubmed/32571831 |
| Flisiak R, Jaroszewicz J, Rogalska M, Lapinski T, Berkan-Kawinska A, Bolewska B, Tudrujek-Zdunek M, Kozielewicz D, Rorat M, Leszczynski P, Klos K, Kowalska J, Pabjan P, Piekarska A, Mozer-Lisewska I, Tomasiewicz K, Paw?owska M, Simon K, Polanska J, Zar?bska-Michaluk D (2021). Tocilizumab Improves the Prognosis of COVID-19 in Patients with High IL-6. J Clin Med 10: 1583. | Flisiak R 2021 |  | http://www.ncbi.nlm.nih.gov/pubmed/33918563 |
| Fonseca SNS, Sousa ADQ, Wolkoff AG, Moreira MS, Pinto BC, Takeda CFV, Reboucas E, Abdon APV, Nascimento ALA, Risch HA (2020). Risk of hospitalization for Covid-19 outpatients treated with various drug regimens in Brazil: Comparative analysis. Travel Med Infect Dis 38: 101906. | Fonseca SNS 2020 |  | http://www.ncbi.nlm.nih.gov/pubmed/33137493 |
| Furtado RHM, Berwanger O, Fonseca HA, Correa TD, Ferraz LR, Lapa MG, Zampieri FG, Veiga VC, Azevedo LCP, Rosa RG, Lopes RD, Avezum A, Manoel ALO, Piza FMT, Martins PA, Lisboa TC, Pereira AJ, Olivato GB, Dantas VCS, Milan EP, Gebara OCE, Amazonas RB, Oliveira MB, Soares RVP, Moia DDF, Piano LPA, Castilho K, Momesso RGRAP, Schettino GPP, Rizzo LV, Neto AS, Machado FR, Cavalcanti AB (2020). Azithromycin in addition to standard of care versus standard of care alone in the treatment of patients admitted to the hospital with severe COVID-19 in Brazil (COALITION II): a randomised clinical trial. Lancet 396: 959-967. | COALITION II | NCT04321278 | http://www.ncbi.nlm.nih.gov/pubmed/32896292 |
| Gagliardini R, Cozzi-Lepri A, Mariano A, Taglietti F, Vergori A, Abdeddaim A, Di Gennaro F, Mazzotta V, Amendola A, D'Offizi G, Palmieri F, Marchioni L, Piselli P, Agrati C, Nicastri E, Capobianchi MR, Petrosillo N, Ippolito G, Vaia F, Girardi E, Antinori A. (2021). No Efficacy of the Combination of Lopinavir/Ritonavir Plus Hydroxychloroquine Versus Standard of Care in Patients Hospitalized With COVID-19: A Non-Randomized Comparison. Front Pharmacol 12: 621676. | Gagliardini R 2021 |  | http://www.ncbi.nlm.nih.gov/pubmed/33967755 |
| Gao G, Wang A, Wang S, Qian F, Chen M, Yu F, Zhang J, Wang X, Ma X, Zhao T, Zhang F, Chen Z (2020). Retrospective Evaluation on the Efficacy of Lopinavir/Ritonavir and Chloroquine to Treat Nonsevere COVID-19 Patients. J Acquir Immune Defic Syndr 85: 239-243. | Gao G 2020 |  | http://www.ncbi.nlm.nih.gov/pubmed/32740371 |
| Garcia-Vidal C, Alonso R, Camon AM, Cardozo C, Albiach L, Aguero D, Marcos MA, Ambrosioni J, Bodro M, Chumbita M, de la Mora L, Garcia-Pouton N, Duenas G, Hernandez-Meneses M, Inciarte A, Cuesta G, Meira F, Morata L, Puerta-Alcalde P, Herrera S, Tuset M, Castro P, Prieto-Gonzalez S, Almuedo-Riera A, Mensa J, Martinez JA, Sanjuan G, Nicolas JM, Del Rio A, Munoz J, Vila J, Garcia F, Soriano A (2021). Impact of remdesivir according to the pre-admission symptom duration in patients with COVID-19. J Antimicrob Chemother 76: 3296-3302. | Garcia-Vidal C 2021 |  | http://www.ncbi.nlm.nih.gov/pubmed/34473275 |
| Garibaldi BT, Wang K, Robinson ML, Betz J, Alexander GC, Andersen KM, Joseph CS, Mehta HB, Korwek K, Sands KE, Fisher AM, Bollinger RC, Xu Y (2021). Real-World Effectiveness Of Remdesivir In Adults Hospitalized With Covid-19: A Retrospective, Multicenter Comparative Effectiveness Study.. Clin Infect Dis: ciab 1035. | Garibaldi BT 2021 |  | http://www.ncbi.nlm.nih.gov/pubmed/34910128 |
| Garibaldi BT, Wang K, Robinson ML, Zeger SL, Roche KB, Wang M, Alexander GC, Gupta A, Bollinger R, Xu Y (2020). Effectiveness of remdesivir with and without dexamethasone in hospitalized patients with COVID-19. medRxiv. | Garibaldi BT 2020 |  | http://doi.org/10.1101/2020.11.19.20234153 |
| Geleris J, Sun Y, Platt J, Zucker J, Baldwin M, Hripcsak G, Labella A, Mason DK, Kubin C, Barr G, Sobieszczyk ME, Schluger NW (2020). Observational Study of Hydroxychloroquine in Hospitalized Patients with Covid-19. N Engl J Med. | Geleris J 2020 |  | https://www.ncbi.nlm.nih.gov/pubmed/32379955?dopt=Citation |
| Gharbharan A, Jordans CC, Geurtsvankessel C, Den HJG, Karim F, Mollema FP, Stalenhoef JE, Dofferhoff A, Ludwig I, Koster A, Hassing R, Bos JC, Van PGR, Vlasveld IN, Ammerlaan HS, Segarceanu E, Miedema J, Van DEM, Papageorgiou G, Te BP, Swaneveld FH, Katsikis PD, Mueller Y, Okba NM, Koopmans MP, Haagmans BL, Rokx C, Rijnders B (2020). Convalescent Plasma for COVID-19. A randomized clinical trial. medRxiv. | CONCOVID | NCT04342182 | https://www.medrxiv.org/content/10.1101/2020.07.01.20139857v1 |
| Gilead Sciences (2021). A Phase 3 Randomized, Double-Blind Placebo-Controlled Trial to Evaluate the Efficacy and Safety of Remdesivir (GS-5734) Treatment of COVID-19 in an Outpatient Setting. clintrials.gov. | GS-US-540-9012 | NCT04501952, EudraCT2020-003510-12 | http://clinicaltrials.gov/show/NCT04501952 |
| Gong Y, Guan L, Jin Z, Chen S, Xiang G, Gao B (2020). Effects of methylprednisolone use on viral genomic nucleic acid negative conversion and CT imaging lesion absorption in COVID-19 patients under 50 years old. J Med Virol. | Gong Y 2020 |  | http://www.ncbi.nlm.nih.gov/pubmed/32441786 |
| Gonzalez JLB, Gamez MG, Enciso EAM , Maldonado RJE , Hernandez PD, Duenas CS, Robles IO, Macias GMJ, Garcia DAL, Gutierrez PCM, Medina LM, Colin VAM , Manuel AGJ (2021). Efficacy and safety of Ivermectin and Hydroxychloroquine in patients with severe COVID-19. A randomized controlled trial. medRxiv. | 2020-A-09 | NCT04391127 | http://doi.org/10.1101/2021.02.18.21252037 |
| Grimaldi D, Aissaoui N, Blonz G, Carbutti G, Courcelle R, Gaudry S, Gaultier A, D'hondt A, Higny J, Horlait G, Hraiech S, Lefebvre L, Lejeune F, Ly A, Piagnerelli M, Sauneuf B, Serck N, Soumagne T, Szychowiak P, Textoris J, Vandenbunder B, Vinsonneau C, Lascarrou JB (2020). Characteristics and outcomes of acute respiratory distress syndrome related to COVID-19 in Belgian and French intensive care units according to antiviral strategies: the COVADIS multicentre observational study. Ann Intensive Care 10: 131. | COVADIS |  | http://www.ncbi.nlm.nih.gov/pubmed/33025225 |
| Guaraldi G, Meschiari M, Cozzi-Lepri A, Milic J, Tonelli R, Menozzi M, Franceschini E, Cuomo G, Orlando G, Borghi V, Santoro A, Di Gaetano M, Puzzolante C, Carli F, Bedini A, Corradi L, Fantini R, Castaniere I, Tabbi L, Girardis M, Tedeschi S, Giannella M, Bartoletti M, Pascale R, Dolci G, Brugioni L, Pietrangelo A, Cossarizza A, Pea F, Clini E, Salvarani C, Massari M, Viale PL, Mussini C (2020). Tocilizumab in patients with severe COVID-19: a retrospective cohort study. Lancet Rheumatol 2: e474-e484. | TESEO |  | http://www.ncbi.nlm.nih.gov/pubmed/32835257 |
| Gupta S, Dixit PK, Ghana P, Abhisheka K, Khurana H, Jha VK, Mahapatra D, Goel J, Ahmed S, Varadaraj G (2021). Open-label randomized control trial of hydroxychloroquine in patients with moderate to severe coronavirus disease 2019 infection. Med J Armed Forces India 77: S305-S311. | Gupta S 2021 | CTRI/2020/04/024479 | http://www.ncbi.nlm.nih.gov/pubmed/34334898 |
| Gupta S, Wang W, Hayek SS, Chan L, Mathews KS, Melamed ML, Brenner SK, Leonberg-Yoo A, Schenck EJ, Radbel J, Reiser J, Bansal A, Srivastava A, Zhou Y, Finkel D, Green A, Mallappallil M, Faugno AJ, Zhang J, Velez JCQ, Shaefi S, Parikh CR, Charytan DM, Athavale AM, Friedman AN, Redfern RE, Short SAP, Correa S, Pokharel KK, Admon AJ, Donnelly JP, Gershengorn HB, Douin DJ, Semler MW, Hernan MA, Leaf DE (2020). Association Between Early Treatment With Tocilizumab and Mortality Among Critically Ill Patients With COVID-19. JAMA Intern Med: e206252. | STOP-COVID |  | http://www.ncbi.nlm.nih.gov/pubmed/33080002 |
| Hamed DM, Belhoul KM, Al Maazmi NA, Ghayoor F, Moin M, Al Suwaidi M, Narainen M, Makki M, AbdulRahman M (2021). Intravenous methylprednisolone with or without tocilizumab in patients with severe COVID-19 pneumonia requiring oxygen support: A prospective comparison.. J Infect Public Health 14: 985-989. | Hamed DM 2021 |  | http://www.ncbi.nlm.nih.gov/pubmed/34153729 |
| Hermine O, Mariette X, Tharaux PL, Resche-Rigon M, Porcher R, Ravaud P (2020). Effect of Tocilizumab vs Usual Care in Adults Hospitalized With COVID-19 and Moderate or Severe Pneumonia: A Randomized Clinical Trial. JAMA Intern Med. | CORIMUNO-TOCI 1 | NCT04331808, EudraCT2020-001246-18 | http://www.ncbi.nlm.nih.gov/pubmed/33080017 |
| Hernandez-Cardenas C, Thirion-Romero I, Rivera-Martinez NE, Meza-Meneses P, Remigio-Luna A, Perez-Padilla R (2021). Hydroxychloroquine for the treatment of severe respiratory infection by covid-19: a randomized controlled trial. medRxiv. | HYDRA | NCT04315896 | http://doi.org/10.1101/2021.02.01.21250371 |
| Hinks TSC, Cureton L, Knight R, Wang A, Cane JL, Barber VS, Black J, Dutton SJ, Melhorn J, Jabeen M, Moss P, Garlapati R, Baron T, Johnson G, Cantle F, Clarke D, Elkhodair S, Underwood J, Lasserson D, Pavord ID, Morgan S, Richards D (2021). A randomised clinical trial of azithromycin versus standard care in ambulatory COVID-19 - the ATOMIC2 trial. medRxiv. | ATOMIC2 | NCT04381962, EudraCT2020-001740-26 | http://doi.org/10.1101/2021.04.21.21255807 |
| Hoertel N, Sanchez M, Vernet R, Beeker N, Neuraz A, Alvarado J, Daniel C, Paris N, Gramfort A, Lemaitre G, Salamanca E, Bernaux M, Bellamine A, Burgun A, Limosin F (2020). Dexamethasone use and Mortality in Hospitalized Patients with Coronavirus Disease 2019: a Multicenter Retrospective Observational Study. medRxiv. | Hoertel N 2020_no oxygen, Hoertel N 2020_oxygen |  | http://doi.org/10.1101/2020.10.23.20218172 |
| Horby P, Lim WS, Emberson J, Mafham M, Bell J, Linsell L, Staplin N, Brightling C, Ustianowski A, Elmahi E, Prudon B, Green C, Felton T, Chadwick D, Rege K, Fegan C, Chappell LC, Faust SN, Jaki T, Jeffery K, Montgomery A, Rowan K, Juszczak E, Baillie JK, Haynes R, Landray MJ (2020). Effect of Dexamethasone in Hospitalized Patients with COVID-19: Preliminary Report. medRxiv. | RECOVERY_dexamethasone | NCT04381936, EudraCT2020-001113-21 | http://doi.org/10.1101/2020.06.22.20137273 |
| Horby P, Mafham M, Linsell L, Bell JL, Staplin N, Emberson JR, Wiselka M, Ustianowski A, Elmahi E, Prudon B, Whitehouse A, Felton T, Williams J, Faccenda J, Underwood J, Baillie JK, Chappell L, Faust SN, Jaki T, Jeffery K, Lim WS, Montgomery A, Rowan K, Tarning J, Watson JA, White NJ, Juszczak E, Haynes R, Landray MJ (2020). Effect of Hydroxychloroquine in Hospitalized Patients with COVID-19: Preliminary results from a multi-centre, randomized, controlled trial. medRxiv. | RECOVERY_Hydroxychloroquine | NCT04381936, EudraCT2020-001113-21, ISRCTN50189673 | https://doi.org/10.1101/2020.07.15.20151852 |
| Horby PW, Estcourt L, Peto L, Emberson JR, Staplin N, Spata E, Amorim GP, Campbell M, Roddick A, Brunskill NE, George T, Zehnder D, Tiberi S, Aung NN, Uriel A, Widdrington J, Koshy G, Brown T, Scott S, Baillie JK, Buch MH, Chappell LC, Day JN, Faust SN, Jaki T, Jeffery K, Juszczak E, Lim WS, Montgomery A, Mumford A, Rowan K, Thwaites G, Mafham M, Roberts D, Haynes R, Landray MJ (2021). Convalescent plasma in patients admitted to hospital with COVID-19 (RECOVERY): a randomised, controlled, open-label, platform trial. medRxiv. | RECOVERY_convalescent plasma | NCT04381936, ISRCTN50189673 | http://doi.org/10.1101/2021.03.09.21252736 |
| Horby PW, Mafham M, Bell JL, Linsell L, Staplin N, Emberson J, Palfreeman A, Raw J,Elmahi E, Prudon B, Green C, Carley S, Chadwick D, Davies M, Wise MP, Baillie JK, Chappell LC, Faust SN, Jaki T, Jeffery K, Lim WS, Montgomery A, Rowan K, Juszczak E, Haynes R, Landray MJ (2020). Lopinavir-ritonavir in patients admitted to hospital with COVID-19 (RECOVERY): a randomised, controlled, open-label, platform trial. Lancet. | RECOVERY_lopinavir | NCT04381936, EudraCT2020-001113-21, ISRCTN50189673 | http://www.ncbi.nlm.nih.gov/pubmed/33031764 |
| Horby PW, Pessoa-Amorim G, Peto L, Brightling CE, Sarkar R, Thomas K, Jeebun V, Ashish A, Tully R, Chadwick D, Sharafat M, Stewart R, Rudran B, Baillie JK, Buch MH, Chappell LC, Day JN, Furst SN, Jaki T, Jeffery K, Juszczak E, Lim WS, Montgomery A, Mumford A, Rowan K, Thwaites G, Mafham M, Haynes R, Landray MJ (2021). Tocilizumab in patients admitted to hospital with COVID-19 (RECOVERY): preliminary results of a randomised, controlled, open-label, platform trial. medRxiv. | RECOVERY_tocilizumab | NCT04381936, EudraCT2020-001113-21, ISRCTN50189673 | http://doi.org/10.1101/2021.02.11.21249258 |
| Horby PW, Roddick A, Spata E, Staplin N, Emberson J, Pessoa-Amorim G, Brightling C, Prudon B, Chadwick D, Ustianowski A, Ashish A, Todd S, Yates B, Buttery R, Scott S, Maseda D, Baillie JK, Buch M, Chappell L, Day J, Faust SN, Jaki T, Jeffery K, Juszczak E, Lim WS, Montgomery A, Mumford A, Rowan K, Thwaites G, Mafham M, Haynes R, Landray MJ (2020). Azithromycin in Hospitalised Patients with COVID-19 (RECOVERY): a randomised, controlled, open-label, platform trial. medRxiv. | RECOVERY_Azithromycin | NCT04381936, EudraCT2020-001113-21, ISRCTN50189673 | http://doi.org/10.1101/2020.12.10.20245944 |
| Hraiech S, Bourenne J, Kuteifan K, Helms J, Carvelli J, Gainnier M, Meziani F, Papazian L (2020). Lack of viral clearance by the combination of hydroxychloroquine and azithromycin or lopinavir and ritonavir in SARS-CoV-2-related acute respiratory distress syndrome. Ann Intensive Care 10: 63. | Hraiech S 2020 |  | http://www.ncbi.nlm.nih.gov/pubmed/32449091 |
| Hu Y, Wang T, Hu Z, Wang X, Zhang Z, Li L, Peng P (2020). Clinical efficacy of glucocorticoid on the treatment of patients with COVID-19 pneumonia: A single-center experience. Biomed Pharmacother 130: 110529. | Hu Y 2020 |  | http://www.ncbi.nlm.nih.gov/pubmed/32736237 |
| Hu Z, Lv Y, Xu C, Sun W, Chen W, Peng Z, Chen C, Cui X, Jiao D, Cheng C, Chi Y, Wei H, Hu C, Zeng Y, Zhang X, Yi Y (2020). Clinical Use of Short-Course and Low-Dose Corticosteroids in Patients With Non-severe COVID-19 During Pneumonia Progression. Front Public Health 8: 355. | Hu Z 2020 |  | http://www.ncbi.nlm.nih.gov/pubmed/32719766 |
| Huang HD, Jneid H, Aziz M, Ravi V, Sharma PS, Larsen T, Chatterjee N, Saour B, Aziz Z, Nayak H, Trohman RG, Krishnan K (2020). Safety and Effectiveness of Hydroxychloroquine and Azithromycin Combination Therapy for Treatment of Hospitalized Patients with COVID-19: A Propensity-Matched Study. Cardiol Ther 9: 523-534. | Huang HD 2020 |  | http://www.ncbi.nlm.nih.gov/pubmed/33058086 |
| Huang R, Zhu C, Wang J, Xue L, Li C, Yan X, Huang S, Zhang B, Zhu L, Xu T, Ming F, Zhao Y, Cheng J, Shao H, Zhao XA, Sang D, Zhao H, Guan X, Chen X, Chen Y, Wei J, Issa R, Liu L, Yan X, Wu C (2020). Corticosteroid therapy is associated with the delay of SARS-CoV-2 clearance in COVID-19 patients. Eur J Pharmacol 889. | Huang R 2020 |  | http://www.ncbi.nlm.nih.gov/pubmed/32941927 |
| Ip A, Ahn J, Zhou Y, Goy AH, Hansen E, Pecora AL, Sinclaire BA, Bednarz U, Marafelias M, Mathura S, Sawczuk IS, Underwood JP, Walker DM, Prasad R, Sweeney RL, Ponce MG, Lacapra S, Cunningham FJ, Calise AG, Pulver BL, Ruocco D, Mojares GE, Eagan MP, Ziontz KL, Mastrokyriakos P, Goldberg SL (2020). Hydroxychloroquine in the treatment of outpatients with mildly symptomatic COVID-19: A multi-center observational study. medRxiv. | Ip A 2020b | NCT04347993 | http://doi.org/10.1101/2020.08.20.20178772 |
| Ip A, Berry DA, Hansen E, Goy AH, Pecora AL, Sinclaire BA, Bednarz U, Marafelias M, Berry SM, Berry NS, Mathura S, Sawczuk IS, Biran N, Go RC, Sperber S, Piwoz JA, Balani B, Cicogna C, Sebti R, Zuckerman J, Rose KM, Tank L, Jacobs L, Korcak J, Timmapuri SL, Underwood JP, Sugalski G, Barsky C, Varga DW, Asif A, Landolfi JC, Goldberg SL (2020). Hydroxychloroquine and Tocilizumab Therapy in COVID-19 Patients - An Observational Study. medRxiv. | Ip A 2020, Ip A 2020_tocilizumab | NCT04347993 | https://doi.org/10.1101/2020.05.21.20109207 |
| Jamaati H, Hashemian SM, Farzanegan B, Malekmohammad M, Tabarsi P, Marjani M, Moniri A, Abtahian Z, Haseli S, Mortaz E, Dastan A, Mohamadnia A, Vahedi A, Monjazebi F, Yassari F, Fadaeizadeh L, Saffaei A, Dastan F (2021). No clinical benefit of high dose corticosteroid administration in patients with COVID-19: A preliminary report of a randomized clinical trial. Eur J Pharmacol 897. | Jamaati H 2021 | IRCT20151227025726N17 | http://www.ncbi.nlm.nih.gov/pubmed/33607104 |
| Jeronimo CMP, Farias MEL, Val FFA, Sampaio VS, Alexandre MAA, Melo GC, Safe IP, Borba MGS, Abreu-Netto RL, Maciel ABS, Neto JRS, Oliveira LB, Figueiredo EFG, Dinelly KMO, Rodrigues MGA, Brito M, Mourao MPG, Pivoto Joao GA, Hajjar LA, Bassat Q, Romero GAS, Naveca FG, Vasconcelos HL, Tavares MA, Brito-Sousa JD, Costa FTM, Nogueira ML, Baia-da-Silva D, Xavier MS, Monteiro WM, Lacerda MVG (2020). Methylprednisolone as Adjunctive Therapy for Patients Hospitalized With COVID-19 (Metcovid): A Randomised, Double-Blind, Phase IIb, Placebo-Controlled Trial. Clin Infect Dis. | Metcovid | NCT04343729 | http://www.ncbi.nlm.nih.gov/pubmed/32785710 |
| Johnston C, Brown ER, Stewart J, Karita HCS, Kissinger PJ, Dwyer J, Hosek S, Oyedele T, Paasche-Orlow MK, Paolino K, Heller KB, Leingang H, Haugen HS, Dong TQ, Bershteyn A, Sridhar AR, Poole J, Noseworthy PA, Ackerman MJ, Morrison S, Greninger AL, Huang ML, Jerome KR, Wener MH, Wald A, Schiffer JT, Celum C, Chu HY, Barnabas RV, Baeten JM (2021). Hydroxychloroquine with or without azithromycin for treatment of early SARS-CoV-2 infection among high-risk outpatient adults: A randomized clinical trial. EClinicalMedicine 33: 100773. | STUDY00009878 | NCT04354428 | http://www.ncbi.nlm.nih.gov/pubmed/33681731 |
| Kalligeros M, Tashima KT, Mylona EK, Rybak N, Flanigan TP, Farmakiotis D, Beckwith CG, Sanchez M, Neill M, Johnson JE, Garland JM, Aung S, Byrd KM, O'Brien T, Pandita A, Aridi J, Macias Gil R, Larkin J, Shehadeh F, Mylonakis E (2020). Remdesivir Use Compared With Supportive Care in Hospitalized Patients With Severe COVID-19: A Single-Center Experience. Open Forum Infect Dis 7: ofaa319. | Kalligeros M 2020 |  | http://www.ncbi.nlm.nih.gov/pubmed/33117850 |
| Karolyi M, Pawelka E, Mader T, Omid S, Kelani H, Ely S, Jilma B, Baumgartner S, Laferl H, Ott C, Traugott M, Turner M, Seitz T, Wenisch C, Zoufaly A (2020). Hydroxychloroquine versus lopinavir/ritonavir in severe COVID-19 patients : Results from a real-life patient cohort. Wien Klin Wochenschr 10: 1-8. | Karolyi M 2020 |  | http://www.ncbi.nlm.nih.gov/pubmed/32776298 |
| Kelly M, O'Connor R, Townsend L, Coghlan M, Relihan E, Moriarty M, Carr B, Melanophy G, Doyle C, Bannan C, O'Riordan R, Merry C, Clarke S, Bergin C (2020). Clinical outcomes and adverse events in patients hospitalised with COVID -19, treated with off- label hydroxychloroquine and azithromycin.. Br J Clin Pharmacol. | Kelly M 2020 |  | http://www.ncbi.nlm.nih.gov/pubmed/32687645 |
| Kewan T, Covut F, Al-Jaghbeer MJ, Rose L, Gopalakrishna KV, Akbik B (2020). Tocilizumab for treatment of patients with severe COVID-19: A retrospective cohort study. EClinicalMedicine 24: 100418. | Kewan T 2020 |  | http://www.ncbi.nlm.nih.gov/pubmed/32766537 |
| Kimmig LM, Wu D, Gold M, Pettit NN, Pitrak D, Mueller J, Husain AN, Mutlu EA, Mutlu GM (2020). IL6 inhibition in critically ill COVID-19 patients is associated with increased secondary infections. medRxiv. | Kimmig LM 2020 |  | http://doi.org/10.1101/2020.05.15.20103531 |
| Kirenga B, Byakika-Kibwika P, Muttamba W, Kayongo A, Loryndah NO, Mugenyi L, Kiwanuka N, Lusiba J, Atukunda A, Mugume R, Ssali F, Ddungu H, Katagira W, Sekibira R, Kityo C, Kyeyune D, Acana S, Aanyu-Tukamuhebwa H, Kabweru W, Nakwagala F, Bagaya BS, Kimuli I, Nantanda R, Buregyeya E, Byarugaba B, Olaro C, Mwebesa H, Joloba ML, Siddharthan T, Bazeyo W (2021). Efficacy of convalescent plasma for treatment of COVID-19 in Uganda.. BMJ Open Respir Res 8. | COVIDIT | NCT04542941 | http://www.ncbi.nlm.nih.gov/pubmed/34376401 |
| Klopfenstein T, Zayet S, Lohse A, Balblanc JC, Badie J, Royer PY, Toko L, Mezher C, Kadiane-Oussou NJ, Bossert M, Bozgan AM, Charpentier A, Roux MF, Contreras R, Mazurier I, Dussert P, Gendrin V, Conrozier T (2020). Tocilizumab therapy reduced intensive care unit admissions and/or mortality in COVID-19 patients.. Med Mal Infect. | Klopfenstein T 2020 |  | https://www.ncbi.nlm.nih.gov/pubmed/32387320 |
| Klopfenstein T, Zayet S, Lohse A, Selles P, Zahra H, Kadiane-Oussou NJ, Toko L, Royer PY, Balblanc JC, Gendrin V, Conrozier T (2020). Impact of tocilizumab on mortality and/or invasive mechanical ventilation requirement in a cohort of 206 COVID-19 patients. Int J Infect Dis  Int J Infect Dis. 99: 491-495. | Klopfenstein T 2020a |  | http://www.ncbi.nlm.nih.gov/pubmed/32798660 |
| Korley FK, Durkalski-Mauldin V, Yeatts SD, Schulman K, Davenport RD, Dumont LJ, El Kassar N, Foster LD, Hah JM, Jaiswal S, Kaplan A, Lowell E, McDyer JF, Quinn J, Triulzi DJ, Van Huysen C, Stevenson VLW, Yadav K, Jones CW, Kea B, Burnett A, Reynolds JC, Greineder CF, Haas NL, Beiser DG, Silbergleit R, Barsan W, Callaway CW (2021). Early Convalescent Plasma for High-Risk Outpatients with Covid-19. N Engl J Med. | SIREN-C3PO | NCT04355767 | http://www.ncbi.nlm.nih.gov/pubmed/34407339 |
| Korper S, Weiss M, Zickler D, Wiesmann T, Zacharowski K, corman VM, Gruner B, Ernst L, Spieth P, Lepper PM, Bentz M, Zinn S, Paul G, Kalbhenn J, Dollinger M, Rosenberger P, Kirschning T, Thiele T, Appl T, Mayer B, Schmidt M, Drosten C, Wulf H, Kruse JM, Jungwirth B, Seifried E, Schrezenmeier H (2021). High Dose Convalescent Plasma in COVID-19: Results from the Randomized Trial CAPSID. medRxiv. | CAPSID | NCT04433910, EudraCT2020-001310-38 | http://doi.org/10.1101/2021.05.10.21256192 |
| Lagier JC, Million M, Gautret P, Colson P, Cortaredona S, Giraud-Gatineau A, Honore S, Gaubert JY, Fournier PE, Tissot-Dupont H, Chabriere E, Stein A, Deharo JC, Fenollar F, Rolain JM, Obadia Y, Jacquier A, La Scola B, Brouqui P, Drancourt M, Parola P, Raoult D (2020). Outcomes of 3,737 COVID-19 patients treated with hydroxychloroquine/azithromycin and other regimens in Marseille, France: A retrospective analysis. Travel Med Infect Dis 36: 101791. | Lagier JC 2020 |  | http://www.ncbi.nlm.nih.gov/pubmed/32593867 |
| Lammers AJJ, Brohet RM, Theunissen REP, Koster C, Rood R, Verhagen DWM, Brinkman K, Hassing RJ, Dofferhoff A, El Moussaoui R, Hermanides G, Ellerbroek J, Bokhizzou N, Visser H, van den Berge M, Bax H, Postma DF, Groeneveld PHP (2020). Early Hydroxychloroquine but not Chloroquine use reduces ICU admission in COVID-19 patients. Int J Infect Dis. | Lammers AJJ 2020 |  | http://www.ncbi.nlm.nih.gov/pubmed/33007454 |
| Lapadula G, Bernasconi DP, Bellani G, Soria A, Rona R, Bombino M, Avalli L, Rondelli E, Cortinovis B, Colombo E, Valsecchi MG, Migliorino GM, Bonfanti P, Foti G (2020). Remdesivir Use in Patients Requiring Mechanical Ventilation due to COVID-19. Open Forum Infect Dis 7. | Lapadula 2020 |  | http://www.ncbi.nlm.nih.gov/pubmed/33204761 |
| Lauriola M, Pani A, Ippoliti G, Mortara A, Milighetti S, Mazen M, Perseghin G, Pastori D, Grosso P, Scaglione F (2020). Effect of Combination Therapy of Hydroxychloroquine and Azithromycin on Mortality in Patients With COVID-19. Clin Transl Sci. | Lauriola M 2020 |  | http://www.ncbi.nlm.nih.gov/pubmed/32926573 |
| Lecronier M, Beurton A, Burrel S, Haudebourg L, Deleris R, Le Marec J, Virolle S, Nemlaghi S, Bureau C, Mora P, De Sarcus M, Clovet O, Duceau B, Grisot PH, Pari MH, Arzoine J, Clarac U, Boutolleau D, Raux M, Delemazure J, Faure M, Decavele M, Morawiec E, Mayaux J, Demoule A, Dres M (2020). Comparison of hydroxychloroquine, lopinavir/ritonavir, and standard of care in critically ill patients with SARS-CoV-2 pneumonia: an opportunistic retrospective analysis. Crit Care 24: 418. | Lecronier M 2020 |  | http://www.ncbi.nlm.nih.gov/pubmed/32653015 |
| Lewis TC, Adhikari S, Tatapudi V, Holub M, Kunichoff D, Troxel AB, Montgomery RA, Sterman DH (2020). A Propensity-Matched Cohort Study of Tocilizumab in Patients With Coronavirus Disease 2019. Crit Care Explor 2: e0283. | Lewis TC 2020 |  | http://www.ncbi.nlm.nih.gov/pubmed/33225307 |
| Li L, Zhang W, Hu Y, Tong X, Zheng S, Yang J, Kong Y, Ren L, Wei Q, Mei H, Hu C, Tao C, Yang R, Wang J, Yu Y, Guo Y, Wu X, Xu Z, Zeng L, Xiong N, Chen L, Wang J, Man N, Liu Y, Xu H, Deng E, Zhang X, Li C, Wang C, Su S, Zhang L, Wang J, Wu Y, Liu Z (2020). Effect of Convalescent Plasma Therapy on Time to Clinical Improvement in Patients With Severe and Life-threatening COVID-19: A Randomized Clinical Trial. JAMA 324: 1-11. | Li L 2020 | ChiCTR2000029757 | https://pubmed.ncbi.nlm.nih.gov/32492084 |
| Li M, Yoo EJ, Baram M, McArthur M, Skeehan C, Awsare B, George G, Summer R, Zurlo J, Jallo J, Roman J (2020). Tocilizumab in the Management of COVID-19: A Preliminary Report. Am J Med Sci S0002-9629: 30491-2. | Li M 2020 |  | http://www.ncbi.nlm.nih.gov/pubmed/33358502 |
| Li Y, Meng Q, Rao X, Wang B, Zhang X, Dong F, Yu T, Li Z, Feng H, Zhang J, Chen X, Li H, Cheng Y, Hong X, Wang X, Yin Y, Zhang Z, Wang D (2020). Corticosteroid therapy in critically ill patients with COVID-19: a multicenter, retrospective study. Crit Care 24: 698. | Li Y 2020 |  | http://www.ncbi.nlm.nih.gov/pubmed/33339536 |
| Li Y, Xie Z, Lin W, Cai W, Wen C, Guan Y, Mo X, Wang J, Wang Y, Peng P, Chen X, Hong W, Xiao G, Liu J, Zhang L, Hu F, Li F, Zhang F, Deng X, Li L (2020). Efficacy and Safety of Lopinavir/Ritonavir or Arbidol in Adult Patients with Mild/Moderate COVID-19: An Exploratory Randomized Controlled Trial. Med (N Y). | ELACOI | NCT04252885 | http://www.ncbi.nlm.nih.gov/pubmed/32838353 |
| Libster R, Perez MG, Wappner D, Coviello S, Bianchi A, Braem V, Esteban I, Caballero MT, Wood CJ, Berrueta M, Rondan A, Lescano G, Cruz P, Ritou I, Fernandez VV, Alvarez PD, Esperante S, Ferretti A, Ofman G, Ciganda A, Rodriguez R, Lantos J, Valentini R, Itcovici N, Hintze A, Oyarvide L, Etchegaray C, Neira A, Name I, Alfonso J, Lopez CR, Caruso G, Rapelius S, Alvez F, Etchenique FC, Dimase F, Alvarez DR, Aranda SS, Sanchez YC, Deluca J, Jarez BS, Laudanno SL, Nowogrodzki F, Izetta F, Paniguetti MT, Fernandez EP, Gutierrez MME, Dominguez V, Balduzzi M, Militerno R, Ochoa J, Perez MS, Dinunzio L, Aizpurua M, Zadoff R, Marchionatti C, Garcia EN, Romero R, Iraizos N, Valls EE, Rearte CP, Franco J, Estrada N, Rusconi J, Ochoa G, Paz MV, Lesch P, Caracciolo MF, Macaneo ME, Pocket L, Marquez S, Pellegrino G, Geffner J, Zarlenga R, Witteveen C, Venditti A, Pichetto OI, Vargas JM, Piani M, Galnarez DC, De LFF, Gamarnik A, Nigro MDC, Villaroel S, Soler RC, Langellotti L, Taffarel C, Scapellato JL, Girasolli M, De ZM, Riera JS, Garcia E, Rovere M, Canela J, Pagella A, Pampuro C, Miragaya Y, Kuperman S, Raggio A, Larrea RM, Silveyra MD, Leberzstein G, Debonis A, Molinos J, Gonzalez M, Perez E, Kreplak N, Pastor AS, Gibbons L, Althabe F, Bergel E, Polack FP (2020). Prevention of severe COVID-19 in the elderly by early high-titer plasma. medRxiv. | FundacionINFANT-Plasma | NCT04479163 | http://doi.org/10.1101/2020.11.20.20234013 |
| Liu J, Zhang S, Dong X, Li Z, Xu Q, Feng H, Cai J, Huang S, Guo J, Zhang L, Chen Y, Zhu W, Du H, Liu Y, Wang T, Chen L, Wen Z, Annane D, Qu J, Chen D (2020). Corticosteroid treatment in severe COVID-19 patients with acute respiratory distress syndrome. J Clin Invest 130: 6417-6428. | Liu J 2020 |  | http://www.ncbi.nlm.nih.gov/pubmed/33141117 |
| Liu L, Qu H, Li JJ, Yang YW, Zeng QX, Gong YW, He ZZ, Zhang YH, Zhang W, Liu B, Che LC (2021). Effectiveness of methylprednisolone therapy in patients with a high-risk common type of COVID-19 pneumonia: a retrospective cohort study. Clin Exp Med: 1-11. | Liu L 2021 |  | http://www.ncbi.nlm.nih.gov/pubmed/34677701 |
| Liu Q, Fang X, Tian L, Chung U, Chen X, Wang K, Li D, Dai X, Zhu Q, Xu F, Shen L, Wang B, Yao L, Peng P (2020). The effect of Arbidol Hydrochloride on reducing mortality of Covid-19 patients: a retrospective study of real-world data from three hospitals in Wuhan. medRxiv. | Liu Q 2020_lopinavir/ritonavir |  | https://doi.org/10.1101/2020.04.11.20056523 |
| Liu STH, Lin HM, Baine I, Wajnberg A, Gumprecht JP, Rahman F, Rodriguez D, Tandon P, Bassily-Marcus A, Bander J, Sanky C, Dupper A, Zheng A, Altman DR, Chen BK, Krammer F, Mendu DR, Firpo-Betancourt A, Levin MA, Bagiella E, Casadevall A, Cordon-Cardo C, Jhang JS, Arinsburg SA, Reich DL, Aberg JA, Bouvier NM (2020). Convalescent plasma treatment of severe COVID-19: A matched control study. medRxiv. | Liu STH 2020 |  | https://doi.org/10.1101/2020.05.20.20102236 |
| Liu Z, Li X, Fan G, Zhou F, Wang Y, Huang L, Yu J, Yang L, Shang L, Xie K, Xu J, Huang Z, Gu X, Li H, Zhang Y, Wang Y, Huang Z, Cao B (2020). Low-to-moderate dose corticosteroids treatment in hospitalized adults with COVID-19. Clin Microbiol Infect. | Liu Z 2020 |  | http://www.ncbi.nlm.nih.gov/pubmed/33007478 |
| Lu X, Chen T, Wang Y, Wang J, Yan F (2020). Adjuvant corticosteroid therapy for critically ill patients with COVID-19. Crit Care 24: 241. | Lu X 2020 |  | http://www.ncbi.nlm.nih.gov/pubmed/32430057 |
| Lyngbakken MN, Berdal JE, Eskesen A, Kvale D, Olsen IC, Rueegg CS, Rangberg A, Jonassen CM, Omland T, Rosjo H, Dalgard O (2020). A pragmatic randomized controlled trial reports lack of efficacy of hydroxychloroquine on coronavirus disease 2019 viral kinetics. Nat Commun 11: 5284. | NO COVID-19 | NCT04316377 | http://www.ncbi.nlm.nih.gov/pubmed/33082342 |
| Ma Q, Qi D, Deng XY, Yuan GD, Tian WG, Cui Y, Yan XF, Wang DX (2020). Corticosteroid therapy for patients with severe novel Coronavirus disease 2019. Eur Rev Med Pharmacol Sci 24: 8194-8201. | Ma Q 2020 |  | http://www.ncbi.nlm.nih.gov/pubmed/32767349 |
| Madan S, Patel A, Sharan K, Ghosh S, Venugopal V, Shah N, Shah B, Thakkar V, Chovatiya R, Shah H, Dabhi P, Patel M, Meghnathi B, Sankhla V, Kapoor V, Patel T, Soni M, Bapat N, Shah K, Chandarana R, Bhatt P, Rana M (2021). Remdesivir for the treatment of COVID-19 disease: A retrospective comparative study of patients treated with and without Remdesivir. medRxiv. | Madan S 2021 | CTRI/2020/05/025247 | https://doi.org/10.1101/2021.07.15.21260600 |
| Magagnoli J, Narendran S, Pereira F, Cummings T, Hardin JW, Sutton SS, Ambati J (2020). Outcomes of hydroxychloroquine usage in United States veterans hospitalized with Covid-19. medRxiv. | Magagnoli J 2020 |  | https://doi.org/10.1101/2020.04.16.20065920 |
| Mahajan L, Singh AP, Gifty NA (2021). Clinical outcomes of using remdesivir in patients with moderate to severe COVID-19: A prospective randomised study. Indian J Anaesth 65: S41-S46. | Mahajan L 2021 |  | http://www.ncbi.nlm.nih.gov/pubmed/33814589 |
| Mahevas M, Tran VT, Roumier M, Chabrol A, Paule R, Guillaud C, Gallien S, Lepeule R, Szwebel TA, Lescure X, Schlemmer F, Matignon M, Khellaf M, Crickx E, Terrier B, Morbieu C, Legendre P, Dang J, Schoindre Y, Pawlotski JM, Michel M, Perrodeau E, Carlier N, Roche N, De Lastours V, Mouthon L, Audureau E, Ravaud P, Godeau B, Costedoat N (2020). No evidence of clinical efficacy of hydroxychloroquine in patients hospitalized for COVID-19 infection with oxygen requirement: results of a study using routinely collected data to emulate a target trial. medRxiv. | Mahevas M 2020 |  | https://doi.org/10.1101/2020.04.10.20060699 |
| Majmundar M, Kansara T, Lenik JM, Park H, Ghosh K, Doshi R, Shah P, Kumar A, Amin H, Chaudhari S, Habtes I (2020). Efficacy of corticosteroids in non-intensive care unit patients with COVID-19 pneumonia from the New York Metropolitan region. PLoS One 15: e0238827. | Majmundar M 2020 |  | http://www.ncbi.nlm.nih.gov/pubmed/32903258 |
| Marrone A, Nevola R, Sellitto A, Cozzolino D, Romano C, Cuomo G, Aprea C, Schwartzbaum MXP, Ricozzi C, Imbriani S, Rinaldi L, Gjeloshi K, Padula A, Ranieri R, Ruosi C, Meo LA, Abitabile M, Cinone F, Carusone C, Adinolfi LE. (2022). Remdesivir plus dexamethasone versus dexamethasone alone for the treatment of COVID-19 patients requiring supplemental O2 therapy: a prospective controlled non randomized study. Clin Infect Dis. | Marrone A 2022 |  | http://www.ncbi.nlm.nih.gov/pubmed/35084022 |
| Martinez-Guerra BA, de-Leon-Cividanes NA, Tamez-Torres KM, Roman-Montes CM, Rajme-Lopez S, Ortiz-Brizuela E, Aguilar-Salinas CA, Sierra-Madero J, Sifuentes-Osornio J, Ponce-de-Leon A, Gonzalez-Lara MF (2021). Effect of Tocilizumab in Mortality among Patients with Severe and Critical Covid-19: Experience in a Third-Level Medical Center.. Rev Invest Clin. | Martinez-Guerra BA 2021 |  | http://www.ncbi.nlm.nih.gov/pubmed/34618802 |
| Martinez-Guerra BA, Gonzalez-Lara MF, Roman-Montes CM, Tamez-Torres KM, Dardon-Fierro FE, Rajme-Lopez S, Medrano-Borromeo C, Martinez-Valenzuela A, Ortiz-Brizuela E, Sifuentes-Osornio J, Ponce-de-Leon A (2021). Outcomes of Patients with Severe and Critical COVID-19 Treated with Dexamethasone: a Prospective Cohort Study. Emerg Microbes Infect 11: 50-59. | Martinez-Guerra BA 2021_corticosteroid |  | http://www.ncbi.nlm.nih.gov/pubmed/34839785 |
| Martinez-Sanz J, Muriel A, Ron R, Herrera S, Perez-Molina JA, Moreno S, Serrano-Villar S (2020). Effects of tocilizumab on mortality in hospitalized patients with COVID-19: A multicenter cohort study. Clin Microbiol Infect S1198-743X: 30573-5. | Martinez-Sanz J 2020 |  | http://www.ncbi.nlm.nih.gov/pubmed/32979572 |
| Masia M, Fernandez-Gonzalez M, Padilla S, Ortega P, Garcia JA, Agullo V, Garcia-Abellan J, Telenti G, Guillen L, Gutierrez F (2020). Impact of interleukin-6 blockade with tocilizumab on SARS-CoV-2 viral kinetics and antibody responses in patients with COVID-19: A prospective cohort study. EBioMedicine. | Masia M 2020 |  | http://www.ncbi.nlm.nih.gov/pubmed/32950003 |
| Menichetti F, Popoli P, Puopolo M, Spila Alegiani S, Tiseo G, Bartoloni A, De Socio GV, Luchi S, Blanc P, Puoti M, Toschi E, Massari M, Palmisano L, Marano G, Chiamenti M, Martinelli L, Franchi S, Pallotto C, Suardi LR, Luciani Pasqua B, Merli M, Fabiani P, Bertolucci L, Borchi B, Modica S, Moneta S, Marchetti G, d'Arminio Monforte A, Stoppini L, Ferracchiato N, Piconi S, Fabbri C, Beccastrini E, Saccardi R, Giacometti A, Esperti S, Pierotti P, Bernini L, Bianco C, Benedetti S, Lanzi A, Bonfanti P, Massari M, Sani S, Saracino A, Castagna A, Trabace L, Lanza M, Focosi D, Mazzoni A, Pistello M, Falcone M (2021). Effect of High-Titer Convalescent Plasma on Progression to Severe Respiratory Failure or Death in Hospitalized Patients With COVID-19 Pneumonia: A Randomized Clinical Trial. JAMA Netw Open 4: e2136246. | TSUNAMI | NCT04716556 | http://www.ncbi.nlm.nih.gov/pubmed/34842924 |
| Menzella F, Fontana M, Salvarani C, Massari M, Ruggiero P, Scelfo C, Barbieri C, Castagnetti C, Catellani C, Gibellini G, Falco F, Ghidoni G, Livrieri F, Montanari G, Casalini E, Piro R, Mancuso P, Ghidorsi L, Facciolongo N (2020). Efficacy of tocilizumab in patients with COVID-19 ARDS undergoing noninvasive ventilation. Crit Care. | Menzella F 2020 |  | http://www.ncbi.nlm.nih.gov/pubmed/32993751 |
| Mert A, Vahaboglu H, Arslan F, Batirel A, Saracoglu KT, Bastug A, Cagatay A, Irmak I, Dizman GT, Ertenli I, Altunal LN, Sengel BE, Bayram M, Omma A, Amikishiyev S, Aypak A, Bes C, Bolukcu S, Icten S, Topeli A, Bektas M, Arslan BY, Ozturk S, Comoglu S, Aydin S, Kucuksahin O, Icacan OC, Ince B, Aghamuradov S, Mutlu MY, Simsek F, Emre S, Ustun C, Ergen P, Aydin O, Koc MM, Sevindik OG, Odabasi Z, Korten V, Bodur H, Guner R, Unal S, Kocak M, Gul A (2021). Tocilizumab treatment in severe COVID-19: a multicenter retrospective study with matched controls.. Rheumatol Int: 1-11. | Mert A 2021 |  | http://www.ncbi.nlm.nih.gov/pubmed/34515808 |
| Mikulska M, Nicolini LA, Signori A, Di Biagio A, Sepulcri C, Russo C, Dettori S, Berruti M, Sormani MP, Giacobbe DR, Vena A, De Maria A, Dentone C, Taramasso L, Mirabella M, Magnasco L, Mora S, Delfino E, Toscanini F, Balletto E, Alessandrini AI, Baldi F, Briano F, Camera M, Dodi F, Ferrazin A, Labate L, Mazzarello G, Pincino R, Portunato F, Tutino S, Barisione E, Bruzzone B, Orsi A, Schenone E, Rosseti N, Sasso E, Da Rin G, Pelosi P, Beltramini S, Giacomini M, Icardi G, Gratarola A, Bassetti M (2020). Tocilizumab and steroid treatment in patients with COVID-19 pneumonia. PLoS ONE 15: e0237831. | Mikulska M 2020 |  | http://www.ncbi.nlm.nih.gov/pubmed/32817707 |
| Mitja O, Corbacho-Monne M, Ubals M, Tebe C, Penafiel J, Tobias A, Ballana E, Alemany A, Riera-Marti N, Perez CA, Suner C, Laporte P, Admella P, Mitja J, Clua M, Bertran L, Sarquella M, Gavilan S, Ara J, Argimon JM, Casabona J, Cuatrecasas G, Canadas P, Elizalde-Torrent A, Fabregat R, Farre M, Forcada A, Flores-Mateo G, Muntada E, Nadal N, Narejos S, Gil-Ortega AN, Prat N, Puig J, Quinones C, Reyes-Urena J, Ramirez-Viaplana F, Ruiz L, Riveira-Munoz E, Sierra A, Velasco C, Vivanco-Hidalgo RM, Sentis A, G-Beiras C, Clotet B, Vall-Mayans M (2020). Hydroxychloroquine for Early Treatment of Adults with Mild Covid-19: A Randomized-Controlled Trial. Clin Infect Dis. | PEP CoV-2 | NCT04304053, EudraCT2020-001031-27 | http://www.ncbi.nlm.nih.gov/pubmed/32674126 |
| Moreno-Garcia E, Rico CV, Albiach L, Aguero D, Ambrosioni J, Bodro M, Cardozo C, Chumbita M, Mora LDL, Garcia-Pouton N, Garcia-Vidal C, Gonzalez-Cordon A, Hernandez-Meneses M, Inciarte A, Laguno M, Leal L, Linares L, Macaya I, Meira F, Mensa J, Moreno A, Morata L, Puerta-Alcalde P, Rojas J, Sola M, Torres B, Torres M, Tome A, Castro P, Fernandez S, Nicolas JM, Almuedo-Riera A, Munoz J, Fernandez-Pittol MJ, Marcos MA, Soy D, Martinez JA, Garcia F, Soriano A (2020). Tocilizumab is associated with reduction of the risk of ICU admission and mortality in patients with SARS-CoV-2 infection. medRxiv. | Moreno-Garcia E 2020 |  | http://doi.org/10.1101/2020.06.05.20113738 |
| Moreno-Perez O, Andres M, Leon-Ramirez JM, Sanchez-Paya J, Rodriguez JC, Sanchez R, Garcia-Sevila R, Boix V, Gil J, Merino E (2020). Experience with tocilizumab in severe COVID-19 pneumonia after 80 days of follow-up: A retrospective cohort study. J Autoimmun 114: 102523. | Moreno-Perez O 2020 |  | http://www.ncbi.nlm.nih.gov/pubmed/32690352 |
| Mozaffari E, Chandak A, Zhang Z, Liang S, Thrun M, Gottlieb RL, Kuritzkes DR, Sax PE, Wohl DA, Casciano R, Hodgkins P, Haubrich R (2021). Remdesivir treatment in hospitalized patients with COVID-19: a comparative analysis of in-hospital all-cause mortality in a large multi-center observational cohort.. Clin Infect Dis. | Mozaffari E 2021 |  | http://www.ncbi.nlm.nih.gov/pubmed/34596223 |
| Munch MW, Meyhoff TS, Helleberg M, Kjaer MN, Granholm A, Hjortsø CJS, Jensen TS, Møller MH, Hjortrup PB, Wetterslev M, Vesterlund GK, Russell L, Jørgensen VL, Tjelle Kristiansen K, Benfield T, Ulrik CS, Andreasen AS, Bestle MH, Poulsen LM, Hildebrandt T, Knudsen LS, Møller A, Sølling CG, Brøchner AC, Rasmussen BS, Nielsen H, Christensen S, Strøm T, Cronhjort M, Wahlin RR, Jakob SM, Cioccari L, Venkatesh B, Hammond N, Jha V, Myatra SN, Jensen MQ, Leistner JW, Mikkelsen VS, Svenningsen JS, Laursen SB, Hatley EV, Kristensen CM, Al-Alak A, Clapp E, Jonassen TB, Bjerregaard CL, Østerby NCH, Jespersen MM, Abou-Kassem D, Lassen ML, Zaabalawi R, Daoud MM, Abdi S, Meier N, la Cour K, Derby CB, Damlund BR, Laigaard J, Andersen LL, Mikkelsen J, Jensen JLS, Rasmussen AH, Arnerlöv E, Lykke M, Holst-Hansen MZB, Tøstesen BW, Schwab J, Madsen EK, Gluud C, Lange T, Perner A (2021). Low-dose hydrocortisone in patients with COVID-19 and severe hypoxia: the COVID STEROID randomised, placebo-controlled trial.. Acta Anaesthesiol Scand. | COVID STEROID | NCT04348305, EudraCT 2020-001395-15 | http://www.ncbi.nlm.nih.gov/pubmed/34138478 |
| National Institute of Allergy and Infectious Diseases (NIAID) (2021). A Randomized, Double-blind, Placebo-controlled Trial to Evaluate the Efficacy of Hydroxychloroquine and Azithromycin to Prevent Hospitalization or Death in Persons With COVID-19. clintrials.gov. | ACTG A5395 | NCT04358068 | http://clinicaltrials.gov/show/NCT04358068 |
| Nelson BC, Laracy J, Shoucri S, Dietz D, Zucker J, Patel N, Sobieszczyk ME, Kubin CJ, Gomez-Simmonds A (2020). Clinical Outcomes Associated with Methylprednisolone in Mechanically Ventilated Patients with COVID-19. Clin Infect Dis. | Nelson 2020 |  | http://www.ncbi.nlm.nih.gov/pubmed/32772069 |
| Nigo M, Rasmy L, May SB, Rao A, Karimaghaei S, Kannadath BS, De la Hoz A, Arias CA, Li L, Zhi D (2021). Real World Long-term Assessment of The Efficacy of Tocilizumab in Patients with COVID19: Results From A Large De-identified Multicenter Electronic Health Record Dataset in the United States.. Int J Infect Dis 113: 148-154. | Nigo M 2021 |  | http://www.ncbi.nlm.nih.gov/pubmed/34597766 |
| Novartis (2021). A Multi-center, Randomized, Double-blinded, Placebo-controlled Study to Evaluate the Safety and Efficacy of Hydroxychloroquine Monotherapy and in Combination With Azithromycin in Patients With Moderate and Severe COVID-19 Disease. clintrials.gov. | CJWT629A12301 | NCT04358081 | http://clinicaltrials.gov/ct2/show/study/NCT04358081 |
| O’donnell MR, Grinsztejn B, Cummings MJ, Justman J, Lamb MR, Eckhardt CM, Philip NM, Cheung YK, Gupta V, Joao E, Pilotto JH, Diniz MP, Cardoso SW, Abrams D, Rajagopalan K, Borden S, Wolf A, Sidi LC, Vizzoni A, Veloso VG, Bitan ZC, Scotto DE, Meyer BJ, Jacobson SD, Kantor A, Mishra N, Chauhan LV, Stone E, Zotti DF, La CF, Hudson KE, Ferrera SA, Schwartz J, Stotler B, Lin WH, Wontakal S, Shaz B, Briese T, Hod EA, Spitalnik SL, Eisenberger A, Lipkin WI (2021). A randomized, double-blind, controlled trial of convalescent plasma in adults with severe COVID-19. medRxiv. | AAAS9924 | NCT04359810 | http://doi.org/10.1101/2021.03.12.21253373 |
| Ohl ME, Miller DR, Lund BC, Kobayashi T, Richardson Miell K, Beck BF, Alexander B, Crothers K, Vaughan Sarrazin MS (2021). Association of Remdesivir Treatment With Survival and Length of Hospital Stay Among US Veterans Hospitalized With COVID-19. JAMA Netw Open 4: e2114741. | Ohl ME 2021 |  | http://www.ncbi.nlm.nih.gov/pubmed/34264329 |
| Okoh AK, Bishburg E, Grinberg S, Nagarakanti S (2020). Tocilizumab use in COVID-19-associated pneumonia. J Med Virol. | Okoh AK 2020 |  | http://www.ncbi.nlm.nih.gov/pubmed/32860432 |
| Olender SA, Perez KK, Go AS, Balani B, Price-Haywood EG, Shah NS, Wang S, Walunas TL, Swaminathan S, Slim J, Chin B, Wit SD, Ali SM, Viladomiu AS, Robinson P, Gottlieb RL, Tsang TYO, Lee IH, Haubrich RH, Chokkalingam AP, Lin L, Zhong L, Bekele BN, Mera-Giler R, Gallant J, Smith LE, Osinusi AO, Brainard DM, Hu H, Phulpin C, Edgar H, Diaz-Cuervo H, Bernardino JI (2020). Remdesivir for Severe COVID-19 versus a Cohort Receiving Standard of Care.. Clin Infect Dis. | GS-US-540-5773+GS-US-540-5807 |  | http://www.ncbi.nlm.nih.gov/pubmed/32706859 |
| Omrani AS, Pathan SA, Thomas SA, Harris TRE, Coyle PV, Thomas CE, Qureshi I, Bhutta ZA, Mawlawi NA, Kahlout RA, Elmalik A, Azad AM, Daghfal J, Mustafa M, Jeremijenko A, Soub HA, Khattab MA, Maslamani MA, Thomas SH (2020). Randomized double-blinded placebo-controlled trial of hydroxychloroquine with or without azithromycin for virologic cure of non-severe Covid-19. EClinicalMedicine 29: 100645. | Q-PROTECT | NCT04349592 | http://www.ncbi.nlm.nih.gov/pubmed/33251500 |
| Omrani AS, Zaqout A, Baiou A, Daghfal J, Elkum N, Alattar RA, Bakdach D, Abusriwil H, Mostafa AM, Alhariri B, Ambra N, Khatib M, Eldeeb AM, Merenkov Z, Fawzi Z, Hmissi SM, Hssain AA, Coyle PV, Alsoub H, Almaslamani MA, Alkhal A (2020). Convalescent Plasma for the Treatment of Patients with Severe Coronavirus Disease 2019; a Preliminary Report. J Med Virol. | Omrani AS 2020 |  | http://www.ncbi.nlm.nih.gov/pubmed/32965715 |
| Owen R, Qizilbash N, Diaz SV, Vazquez JMC, Pocock S (2021). Making sense of non-randomized comparative treatment studies in times of Covid-19: A case study of tocilizumab. medRxiv. | Owen R 2021 |  | http://doi.org/10.1101/2021.04.06.21254612 |
| Paccoud O, Tubach F, Baptiste A, Bleibtreu A, Hajage D, Monsel G, Tebano G, Boutolleau D, Klement E, Godefroy N, Palich R, Itani O, Fayssal A, Valantin MA, Tubiana R, Burrel S, Calvez V, Caumes E, Marcelin AG, Pourcher V (2020). Compassionate use of hydroxychloroquine in clinical practice for patients with mild to severe Covid-19 in a French university hospital. Clin Infect Dis. | Paccoud O 2020 |  | http://www.ncbi.nlm.nih.gov/pubmed/32556143 |
| Pan H, Peto R, Karim QA, Alejandria M, Henao-Restrepo AM, García CH, Kieny M-P, Malekzadeh R, Murthy S, Preziosi M-P, Reddy S, Periago MR, Sathiyamoorthy V, Røttingen J-A, Swaminathan S (2020). Repurposed antiviral drugs for COVID-19; interim WHO SOLIDARITY trial results. medRxiv. | SOLIDARITY_hydroxychloroquine, SOLIDARITY_lopinavir, SOLIDARITY_remdesivir | NCT04315948, ISRCTN83971151 | https://www.medrxiv.org/content/10.1101/2020.10.15.20209817v1 |
| Papamanoli A, Yoo J, Grewal P, Predun W, Hotelling J, Jacob R, Mojahedi A, Skopicki HA, Mansour M, Marcos LA, Kalogeropoulos AP (2020). High-Dose Methylprednisolone in Nonintubated Patients with Severe COVID-19 Pneumonia. Eur J Clin Invest: e13458. | Papamanoli A 2020 |  | http://www.ncbi.nlm.nih.gov/pubmed/33219551 |
| Pasquini Z, Montalti R, Temperoni C, Canovari B, Mancini M, Tempesta M, Pimpini D, Zallocco N, Barchiesi F (2020). Effectiveness of remdesivir in patients with COVID-19 under mechanical ventilation in an Italian ICU. J. Antimicrob. Chemother.. | Pasquini Z 2020 |  | http://www.ncbi.nlm.nih.gov/pubmed/32829390 |
| Perotti C, Baldanti F, Bruno R, Del Fante C, Seminari E, Casari S, Percivalle E, Glingani C, Musella V, Belliato M, Garuti M, Meloni F, Frigato M, Di Sabatino A, Klersy C, De Donno G, Franchini M (2020). Mortality reduction in 46 severe Covid-19 patients treated with hyperimmune plasma. A proof of concept single arm multicenter trial.. Haematologica. | Perotti C 2020 | NCT04321421 | http://www.ncbi.nlm.nih.gov/pubmed/32703797 |
| Perrone F, Piccirillo MC, Ascierto PA, Salvarani C, Parrella R, Marata AM, Popoli P, Ferraris L, Marrocco TMM, Ripamonti D, Binda F, Bonfanti P, Squillace N, Castelli F, Muiesan ML, Lichtner M, Calzetti C, Salerno ND, Atripaldi L, Cascella M, Costantini M, Dolci G, Facciolongo NC, Fraganza F, Massari M, Montesarchio V, Mussini C, Negri EA, Botti G, Cardone C, Gargiulo P, Gravina A, Schettino C, Arenare L, Chiodini P, Gallo C (2020). Tocilizumab for patients with COVID-19 pneumonia. The TOCIVID-19 prospective phase 2 trial. medRxiv. | TOCIVID-19_phase 2, TOCIVID-19_validation | NCT04317092, EudraCT2020-001110-38 | https://doi.org/10.1101/2020.06.01.20119149 |
| Potere N, Di Nisio M, Cibelli D, Scurti R, Frattari A, Porreca E, Abbate A, Parruti G (2020). Interleukin-6 receptor blockade with subcutaneous tocilizumab in severe COVID-19 pneumonia and hyperinflammation: a case-control study. Ann Rheum Dis. | Potere N 2020 |  | http://www.ncbi.nlm.nih.gov/pubmed/32647027 |
| Potere N, Nisio MD, Rizzo G, Vella ML, Polilli E, Agostinone A, Spacone A, Carlo SD, Costantini A, Abbate A, Porreca E, Parruti G (2020). Low-dose subcutaneous tocilizumab to prevent disease progression in patients with moderate COVID-19 pneumonia and hyperinflammation. Int J Infect Dis: 421-424. | Potere N 2020b |  | http://www.ncbi.nlm.nih.gov/pubmed/32768701 |
| Pouladzadeh M, Safdarian M, Eshghi P, Abolghasemi H, Bavani AG, Sheibani B, Moradi Choghakabodi P, Feghhi A, Ghafourian Boroujerdnia M, Forouzan A, Jalali Far MA, Kaydani GA, Rajaei E, Amin M, Torabizadeh M, Yousefi F, Hadaddezfuli R (2021). A randomized clinical trial evaluating the immunomodulatory effect of convalescent plasma on COVID-19-related cytokine storm. Intern Emerg Med: 1-11. | Pouladzadeh M 2021 | IRCT20200310046736N1 | http://www.ncbi.nlm.nih.gov/pubmed/33837906 |
| Priscilla Hsue, MD (2021). A Randomized Controlled Adaptive Study Comparing COVID-19 Convalescent Plasma (CCP) to Non-immune Plasma to Limit Coronavirus-associated Complications in Hospitalized Patients. clintrials.gov. | CAPRI | NCT04421404 | http://clinicaltrials.gov/ct2/show/NCT04421404 |
| Quartuccio L, Sonaglia A, McGonagle D, Fabris M, Peghin M, Pecori D, Monte A, Bove T, Curcio F, Bassi F, Vita S, Tascini C (2020). Profiling COVID-19 Pneumonia Progressing Into the Cytokine Storm Syndrome: Results From a Single Italian Centre Study on Tocilizumab Versus Standard of Care. J Clin Virol. | Quartuccio L 2020 | EudraCT2020-001110-38 | https://pubmed.ncbi.nlm.nih.gov/32425662 |
| Rahman O, Trigonis RA, Craft MK, Kruer RM, Miller EM, Terry CL, Persaud SA, Kapoor R (2020). Corticosteroid Use in Severely Hypoxemic COVID-19 Patients: An Observational Cohort Analysis of Dosing Patterns and Outcomes in the Early Phase of the Pandemic. medRxiv. | Rahman O 2020 |  | http://doi.org/10.1101/2020.07.29.20164277 |
| Rajendram P, Sacha GL, Mehkri O, Wang X, Han X, Vachharajani V, Duggal A (2021). Tocilizumab in Coronavirus Disease 2019-Related Critical Illness: A Propensity Matched Analysis.. Crit Care Explor 3: e0327. | Rajendram P 2021 |  | http://www.ncbi.nlm.nih.gov/pubmed/33490955 |
| Ramaswamy M, Mannam P, Comer R, Sinclair E, McQuaid DB, Schmidt ML (2020). Off-Label Real World Experience Using Tocilizumab for Patients Hospitalized with COVID-19 Disease in a Regional Community Health System: A Case-Control Study. medRxiv. | Ramaswamy M 2020 |  | http://doi.org/10.1101/2020.05.14.20099234 |
| Ramiro S, Mostard RLM, Magro-Checa C, van Dongen CMP, Dormans T, Buijs J, Gronenschild M, de Kruif MD, van Haren EHJ, van Kraaij T, Leers MPG, Peeters R, Wong DR, Landewe RBM (2020). Historically controlled comparison of glucocorticoids with or without tocilizumab versus supportive care only in patients with COVID-19-associated cytokine storm syndrome: results of the CHIC study.. Ann Rheum Dis 79: 1143-1151. | CHIC study |  | http://www.ncbi.nlm.nih.gov/pubmed/32719045 |
| Rashad A, Nafady A, Hassan MH, Mansour H, Taya U, Bazeed SES, Aref ZF, Sayed MAA, Nafady-Hego H, Abdelmaksoud AA (2021). Therapeutic efficacy of macrolides in management of patients with mild COVID-19. Sci Rep 11: 16361. | SVU-MED-CHT019-420860 | NCT04622891 | http://www.ncbi.nlm.nih.gov/pubmed/34381155 |
| Rasheed AM, Fatak DF, Hashim HA, Maulood MF, Kabah KK, Almusawi YA, Abdulamir AS (2020). The therapeutic potential of convalescent plasma therapy on treating critically-ill COVID-19 patients residing in respiratory care units in hospitals in Baghdad, Iraq. Infez Med 28: 357-366. | Rasheed AM 2020 | NCT04441424 | http://www.ncbi.nlm.nih.gov/pubmed/32920571 |
| Ray Y, Paul SR, Bandopadhyay P, D'rozario R, Sarif J, Lahiri A, Bhowmik D, Vasudevan JS, Maurya R, Kanakan A, Sharma S, Kumar M, Singh P, Roy R, Chaudhury K, Maiti R, Bagchi S, Maiti A, Perwez MM, Mondal A, Tewari A, Mandal S, Roy A, Saha M, Biswas D, Maiti C, Chakraborty S, Sarkar BS, Haldar A, Saha B, Sengupta S, Pandey R, Chatterjee S, Bhattacharya P, Paul S, Ganguly D (2020). Clinical and immunological benefits of convalescent plasma therapy in severe COVID-19: insights from a single center open label randomised control trial. medRxiv. | Ray Y 2020 | CTRI/2020/05/025209 | http://doi.org/10.1101/2020.11.25.20237883 |
| Reis G, Silva EADSM, Silva DCM, Thabane L, Singh G, Park JJH, Forrest JI, Harari O, Santos CVQD, de Almeida APFG, Neto ADDF, Savassi LCM, Milagres AC, Teixeira MM, Simplicio MIC, Ribeiro LB, Oliveira R, Mills EJ (2021). Effect of Early Treatment With Hydroxychloroquine or Lopinavir and Ritonavir on Risk of Hospitalization Among Patients With COVID-19: The TOGETHER Randomized Clinical Trial. JAMA Netw Open 4: e216468. | TOGETHER_hydroxychloroquine | NCT04403100 | http://www.ncbi.nlm.nih.gov/pubmed/33885775 |
| Rodrigues C, Freitas-Santos RS, Levi JE, Senerchia AA, Lopes ATA, Santos SR, Siciliano RF, Pierrotti LC (2021). Hydroxychloroquine plus azithromycin early treatment of mild COVID-19 in outpatient setting: a randomized, double-blinded, placebo-controlled clinical trial evaluating viral clearance. Int J Antimicrob Agents: 106428. | Rodrigues C 2021 |  | http://www.ncbi.nlm.nih.gov/pubmed/34454044 |
| Rodriguez-Bano J, Pachon J, Carratala J, Ryan P, Jarrin I, Yllescas M, Arribas JR, Berenguer J (2020). Treatment with tocilizumab or corticosteroids for COVID-19 patients with hyperinflammatory state: a multicentre cohort study (SAM-COVID-19). Clin Microbiol Infect: 30492-4. | SAM-COVID | NCT04355871 | http://www.ncbi.nlm.nih.gov/pubmed/32860964 |
| Rogers R, Shehadeh F, Mylona EK, Rich J, Neill M, Touzard-Romo F, Geffert S, Larkin J, Bailey JA, Lu S, Sweeney J, Mylonakis E (2020). Convalescent plasma for patients with severe COVID-19: a matched cohort study. Clin Infect Dis: ciaa1548. | Rogers R 2020 |  | http://www.ncbi.nlm.nih.gov/pubmed/33038227 |
| Rojas-Marte GR, Khalid M, Mukhtar O, Hashmi AT, Waheed MA, Ehrlich S, Aslam A, Siddiqui S, Agarwal C, Malyshev Y, Henriquez-Felipe C, Sharma D, Sharma S, Chukwuka N, Rodriguez DC, Alliu S, Le J, Shani J (2020). Outcomes in patients with severe COVID-19 disease treated with tocilizumab: a case-controlled study. QJM 113: 546-550. | Rojas-Marte GR 2020 |  | http://www.ncbi.nlm.nih.gov/pubmed/32569363 |
| Roman JMG, Garcia SCR, Vallejo ER, Jimenez A, Alonso SS, Diaz CF, Serna AA, Albero TM, Cortes PR, Cerrillo IS, Esparcia L, Fleta PM, Sanz CL, Gabrie L, Guerola LDC, Suarez C, Ancochea J, Canabal A, Albert P, Serrano DAR, Aguilar JM, Arco CD, Santos IL, Fraile LG, Camara R, Serra JM, Ramirez E, Alonso T, Landete P, Soriano JB, Gayo ME, Torres AF, Cruz NDZ, Vicuna RG, Cardenoso L, Madrid FS, Alfranca A, Calleja CM, Alvaro IG (2020). IL-6 serum levels predict severity and response to Tocilizumab in COVID-19: an observational study. J Allergy Clin Immunol. | Roman JMG 2020 |  | http://www.ncbi.nlm.nih.gov/pubmed/33010257 |
| Romon I, Dominguez-Garcia JJ, Arroyo JL, Suberviola B, Cabezon I, Abascal B, Baldeon C, Cuesta A, Portilla R, Casuso E, Ocio E, Briz M (2021). Convalescent plasma treatment for patients of 80 years and older with COVID-19 pneumonia. BMC Geriatr 21: 566. | Romon I 2021 |  | http://www.ncbi.nlm.nih.gov/pubmed/34663227 |
| Rosas IO, Brau N, Waters M, Go R, Hunter BD, Bhagani S, Skiest D, Aziz MS, Cooper N, Douglas IS, Savic S, Youngstein T, Sorbo LD, Gracian AC, Zerda DJDL, Ustianowski A, Bao M, Dimonaco S, Graham E, Matharu B, Spotswood H, Tsai L, Malhotra A (2021). Tocilizumab in Hospitalized Patients with Severe Covid-19 Pneumonia. N Engl J Med. | COVACTA | NCT04320615, EudraCT2020-001154-22 | http://www.ncbi.nlm.nih.gov/pubmed/33631066 |
| Rosas IO, Diaz G, Gottlieb RL, Lobo SM, Robinson P, Hunter BD, Cavalcante AW, Overcash JS, Hanania NA, Skarbnik A, Garcia-Diaz J, Gordeev I, Carratala J, Gordon O, Graham E, Lewin-Koh N, Tsai L, Tuckwell K, Cao H, Brainard D, Olsson JK (2021). Tocilizumab and remdesivir in hospitalized patients with severe COVID-19 pneumonia: a randomized clinical trial.. Intensive Care Med: 1-13. | REMDACTA | NCT04409262 | http://www.ncbi.nlm.nih.gov/pubmed/34609549 |
| Rosenberg ES, Dufort EM, Udo T, Wilberschied LA, Kumar J, Tesoriero J, Weinberg P, Kirkwood J, Muse A, DeHovitz J, Blog DS, Hutton B, Holtgrave DR, Zucker HA (2020). Association of Treatment With Hydroxychloroquine or Azithromycin With In-Hospital Mortality in Patients With COVID-19 in NewYork State. JAMA. | Rosenberg ES 2020 |  | http://www.ncbi.nlm.nih.gov/pubmed/32392282 |
| Roumier M, Paule R, Vallée A, Rohmer J, Ballester M, Brun AL, Cerf C, Chabi ML, Chinet T, Colombier MA, Farfour E, Fourn E, Géri G, Khau D, Marroun I, Ponsoye M, Roux A, Salvator H, Schoindre Y, Si Larbi AG, Tchérakian C, Vasse M, Verrat A, Zuber B, Couderc LJ, Kahn JE, Groh M, Ackermann F (2020). Tocilizumab for Severe Worsening COVID-19 Pneumonia: a Propensity Score Analysis. J Clin Immunol. | TOCICOVID |  | http://www.ncbi.nlm.nih.gov/pubmed/33188624 |
| Ruiz-Antoran B, Sancho-Lopez A, Torres F, Moreno-Torres V, Pablo-Lopez Id, Garcia-Lopez P, Abad-Santos F, Rosso-Fernandez CM, Aldea-Perona A, Montane E, Aparicio-Hernandez RM, Llop-Rius R, Pedros C, Gijon P, Hernandez-Carballo C, Pedrosa-Martinez MJ, Rodriguez-Jimenez C, Prada-Ramallal G, Cabrera-Garcia L, Aguilar-Garcia JA, Sanjuan-Jimenez R, Ortiz-Barraza EI, Sanchez-Chica E, Fernandez-Cruz A (2020). Combination of Tocilizumab and Steroids to Improve Mortality in Patients with Severe COVID-19 Infection: A Spanish, Multicenter, Cohort Study.. Infect Dis Ther: 1-16. | TOCICOV |  | http://www.ncbi.nlm.nih.gov/pubmed/33280066 |
| Russo A, Binetti E, Borrazzo C, Cacciola EG, Battistini L, Ceccarelli G, Mastroianni CM, d'Ettorre G (2021). Efficacy of Remdesivir-Containing Therapy in Hospitalized COVID-19 Patients: A Prospective Clinical Experience. J Clin Med 10. | Russo A 2021 |  | http://www.ncbi.nlm.nih.gov/pubmed/34501233 |
| Russo G, Solimini A, Zuccala P, Zingaropoli MA, Carraro A, Pasculli P, Perri V, Marocco R, Kertusha B, Del Borgo C, Del Giudice E, Fondaco L, Tieghi T, D'Agostino C, Oliva A, Vullo V, Ciardi MR, Mastroianni CM, Lichtner M (2021). Real-life use of tocilizumab with or without corticosteroid in hospitalized patients with moderate-to-severe COVID-19 pneumonia: A retrospective cohort study.. PLoS One 16: e0257376. | Russo G 2021 |  | http://www.ncbi.nlm.nih.gov/pubmed/34506608 |
| Rutgers A, Westerweel PE, Holt BVD, Postma S, Vonderen MGAV, Piersma DP, Postma D, Berge MVD, Jong E, Vries MD, Burg LVD, Huugen D, Poel MVD, Kampschreur LM, Nijland M, Strijbos JH, Tamminga M, Mutsaers PGNJ, Schol-Gelok S, Dijkstra-Tiekstra M, Sidorenkov G, Vincenten J, Geffen WHV, Knoester M, Kosterink J, Gans R, Stegeman C, Huls G, Meerten TV (2021). Timely administration of tocilizumab improves survival of hospitalized COVID-19 patients. . | PreToVid | NL8504, EudrCT2020-001375-149 | http://dx.doi.org/10.2139/ssrn.3834311 |
| Salama C, Han J, Yau L, Reiss WG, Kramer B, Neidhart JD, Criner GJ, Kaplan LE, Baden R, Pandit L, Cameron ML, Garcia DJ, Chavez V, Mekebeb RM, Menezes FL, Shah R, Gonzalez LMF, Assman B, Freedman J, Mohan SV (2020). Tocilizumab in Patients Hospitalized with Covid-19 Pneumonia. N Engl J Med 384: 20-30. | EMPACTA | NCT04372186 | http://www.ncbi.nlm.nih.gov/pubmed/33332779 |
| Salazar E, Christensen PA, Graviss EA, Nguyen DT, Castillo B, Chen J, Lopez BV, Eagar TN, Yi X, Zhao P, Rogers J, Shehabeldin A, Joseph D, Leveque C, Olsen RJ, Bernard DW, Gollihar J, Musser JM (2020). Treatment of COVID-19 Patients with Convalescent Plasma Reveals a Signal of Significantly Decreased Mortality. Am J Pathol. | Salazar E 2020b |  | http://www.ncbi.nlm.nih.gov/pubmed/32795424 |
| Salazar MR, Gonzalez SE, Regairaz L, Ferrando NS, Martinez VVG, Ramos PMC, Munoz L, Pesci SA, Vidal JM, Kreplak N, Estenssoro E (2020). EFFECT OF CONVALESCENT PLASMA ON MORTALITY IN PATIENTS WITH COVID-19 PNEUMONIA. medRxiv. | Salazar MR 2020 |  | http://doi.org/10.1101/2020.10.08.20202606 |
| Saliaj M, Li M, Rizzo V, Nassar M, Nso N, Idrees Z, Medina AL, Novikov A, Yaghi S, Cuevas FJ, Salafia CM, Razaki H, Berman C, Sachmechi I, Trandafirescu T, Brennessel D, Martin GT, Mantis J (2021). Tocilizumab Treatment Reduces Mortality in Hospitalized Patients with COVID-19. Research Square. | Saliaj M 2021 |  | https://doi.org/10.21203/rs.3.rs-752079/v2 |
| Salton F, Confalonieri P, Meduri GU, Santus P, Harari S, Scala R, Lanini S, Vertui V, Oggionni T, Caminati A, Patruno V, Tamburrini M, Scartabellati A, Parati M, Villani M, Radovanovic D, Tomassetti S, Ravaglia C, Poletti V, Vianello A, Gaccione AT, Guidelli L, Raccanelli R, Lucernoni P, Lacedonia D, Foschino Barbaro MP, Centanni S, Mondoni M, Davì M, Fantin A, Cao X, Torelli L, Zucchetto A, Montico M, Casarin A, Romagnoli M, Gasparini S, Bonifazi M, D'Agaro P, Marcello A, Licastro D, Ruaro B, Volpe MC, Umberger R, Confalonieri M (2020). Prolonged Low-Dose Methylprednisolone in Patients With Severe COVID-19 Pneumonia.. Open Forum Infect Dis 7: ofaa421. | MP-C19 | NCT04323592 | http://www.ncbi.nlm.nih.gov/pubmed/33072814 |
| Salvarani C, Dolci G, Massari M, Merlo DF, Cavuto S, Savoldi L, Bruzzi P, Boni F, Braglia L, Turra C, Ballerini PF, Sciascia R, Zammarchi L, Para O, Scotton PG, Inojosa WO, Ravagnani V, Salerno ND, Sainaghi PP, Brignone A, Codeluppi M, Teopompi E, Milesi M, Bertomoro P, Claudio N, Salio M, Falcone M, Cenderello G, Donghi L, Del Bono V, Colombelli PL, Angheben A, Passaro A, Secondo G, Pascale R, Piazza I, Facciolongo N, Costantini M (2020). Effect of Tocilizumab vs Standard Care on Clinical Worsening in Patients Hospitalized With COVID-19 Pneumonia: A Randomized Clinical Trial. JAMA Intern Med: e206615. | RCT-TCZ-COVID-19 | NCT04346355, EudraCT2020-001386-37 | http://www.ncbi.nlm.nih.gov/pubmed/33080005 |
| Sands K, Wenzel R, McLean L, Korwek K, Roach J, Miller K, Poland RE, Burgess LH, Jackson E, Perlin JB. No clinical benefit in mortality associated with hydroxychloroquine treatment in patients with COVID-19. Int J Infect Dis. | Sands K 2020 |  | http://www.ncbi.nlm.nih.gov/pubmed/33359949 |
| Sbidian E, Josse J, Lemaitre G, Mayer I, Bernaux M, Gramfort A, Lapidus N, Paris N, Neuraz A, Lerner I, Garcelon N, Rance B, Grisel O, Moreau T, Bellamine A, Wolkenstein P, Varoquaux G, Caumes E, Lavielle M, Mekontso DA, Audureau E (2020). Hydroxychloroquine with or without azithromycin and in-hospital mortality or discharge in patients hospitalized for COVID-19 infection: a cohort study of 4,642 in-patients in France. medRxiv. | Sbidian E 2020 |  | http://doi.org/10.1101/2020.06.16.20132597 |
| Schwartz I, Boesen ME, Cerchiaro G, Doram C, Edwards BD, Ganesh A, Greenfield J, Jamieson S, Karnik V, Kenney C, Lim R, Menon BK, Mponponsuo K, Rathwell S, Ryckborst KJ, Stewart B, Yaskina M, Metz L, Richer L, Hill MD (2021). Assessing the efficacy and safety of hydroxychloroquine as outpatient treatment of COVID-19: a randomized controlled trial.. CMAJ Open 9: E693-E702. | ABCOV-01 version 1.5 | NCT04329611 | http://ncbi.nlm.nih.gov/pubmed/34145052 |
| Sekhavati E, Jafari F, SeyedAlinaghi S, Siahkali SJM, Sadr S, Tabarestani M, Pirhayati M, Zendehdel A, Manafi N, Hajiabdolbaghi M, Ahmadinejad Z, Kouchak HE, Jafari S, Khalili H, Salehi M, Seifi A, Golestan FS, Ghiasvand F (2020). Safety and effectiveness of azithromycin in patients with COVID-19: An open-label randomised trial. Int J Antimicrob Agents 56: 106143. | Sekhavati E 2020 | IRCT20200415047092N1 | http://www.ncbi.nlm.nih.gov/pubmed/32853672 |
| Sekine L, Arns B, Fabro BR, Cipolatt MM, Machado RRG, Durigon EL, Edino Parolo E, Pellegrini JAS, Viana MV, Schwarz P, Lisboa TC, Dora JMS, Balsan AM, Schirmer FDS, Franz JPM, Silveira LMD, Breunig RC, Petersen V, Sosnoski M, Mesquita NF, Volpato FCZ, Sganzerla D, Falavigna M, Rosa RG, Zavascki AP (2021). Convalescent plasma for COVID-19 in hospitalised patients: an open-label, randomised clinical trial. Eur Respir J: 2101471. | PLACOVID | NCT04547660 | http://www.ncbi.nlm.nih.gov/pubmed/34244316 |
| Self WH, Semler MW, Leither LM, Casey JD, Angus DC, Brower RG, Chang SY, Collins SP, Eppensteiner JC, Filbin MR, Files DC, Gibbs KW, Ginde AA, Gong MN, Harrell FE, Hayden DL, Hough CL, Johnson NJ, Khan A, Lindsell CJ, Matthay MA, Moss M, Park PK, Rice TW, Robinson BRH, Schoenfeld DA, Shapiro NI, Steingrub JS, Ulysse CA, Weissman A, Yealy DM, Thompson BT, Brown SM, Steingrub J, Smithline H, Tiru B, Tidswell M, Kozikowski L, Thornton-Thompson S, De Souza L, Hou P, Baron R, Massaro A, Aisiku I, Fredenburgh L, Seethala R, Johnsky L, Riker R, Seder D, May T, Baumann M, Eldridge A, Shapiro N, Talmor D, O'Mara T, Kirk C, Harrison K, Kurt L, Schermerhorn M, Banner-Goodspeed V, Boyle K, Dubosh N, Filbin M, Hibbert K, Parry B, Lavin-Parsons K, Pulido N, Lilley B, Lodenstein C, Margolin J, Brait K, Jones A, Galbraith J, Peacock R, Nandi U, Wachs T, Matthay M, Liu K, Kangelaris K, Wang R, Calfee C, Yee K, Hendey G, Chang S, Lim G, Qadir N, Tam A, Beutler R, Levitt J, Wilson J, Rogers A, Vojnik R, Roque J, Albertson T, Chenoweth J, Adams J, Pearson S, Juarez M, Almasri E, Fayed M, Hughes A, Hillard S, Huebinger R, Wang H, Vidales E, Patel B, Ginde A, Moss M, Baduashvili A, McKeehan J, Finck L, Higgins C, Howell M, Douglas I, Haukoos J, Hiller T, Lyle C, Cupelo A, Caruso E, Camacho C, Gravitz S, Finigan J, Griesmer C, Park P, Hyzy R, Nelson K, McDonough K, Olbrich N, Williams M, Kapoor R, Nash J, Willig M, Ford H, Gardner-Gray J, Ramesh M, Moses M, Ng Gong M, Aboodi M, Asghar A, Amosu O, Torres M, Kaur S, Weiler M, Chen JT, Hope A, Lopez B, Rosales K, You JY, Mosier J, Hypes C, Natt B, Borg B, Salvagio E, Hite RD, Hudock K, Cresie A, Alhasan F, Gomez-Arroyo A, Duggal O, Mehkri A, Hastings D, Sahoo F, Abi Fadel S, Gole V, Shaner A, Wimer Y, Meli A, King T, Terndrup M, Exline S, Pannu E, Robart S, Karow C, Hough B, Robinson N, Johnson D, Henning M, Campo S, Gundel S, Seghal S, Katsandres S, Dean A, Khan O, Krol M, Jouzestani P, Huynh A, Weissman D, Yealy D, Scholl P, Adams B, McVerry D, Huang D, Angus J, Schooler S, Moore DC, Files C, Miller K, Gibbs M, LaRose L, Flores L, Koehler C, Morse J, Sanders C, Langford K, Nanney M, MdalaGausi P, Yeboah P, Morris J, Sturgill S, Seif E, Cassity S, Dhar M, de Wit J, Mason A, Goodwin G, Hall A, Grady A, Chamberlain S, Brown J, Bledsoe L, Leither I, Peltan N, Starr M, Fergus V, Aston Q, Montgomery R, Smith M, Merrill K, Brown B, Armbruster E, Harris E, Middleton R, Paine S, Johnson M, Barrios J, Eppensteiner A, Limkakeng L, McGowan T, Porter A, Bouffler JC, Leahy B, deBoisblanc M, Lammi K, Happel P, Lauto W, Self J, Case M, Semler S, Collins F, Harrell C, Lindsell T, Rice W, Stubblefield C, Gray J, Johnson M, Roth M, Hays D, Torr A, Zakaria D, Schoenfeld BT, Thompson D, Hayden N, Ringwood C, Oldmixon C, Ulysse R, Morse A, Muzikansky L, Fitzgerald S, Whitaker A, Lagakos L, Reineck N, Aggarwal K, Bienstock M, Freemer M, Maclawiw G, Weinmann L, Morrison M, Gillespie R, Kryscio D, Brodie W, Zareba A, Rompalo M, Boeckh P, Parsons J, Christie J, Hall J, Horton N, Zoloth L, Dickert N, Diercks D (2020). Effect of Hydroxychloroquine on Clinical Status at 14 Days in Hospitalized Patients With COVID-19 A Randomized Clinical Trial. JAMA 324: 2165-2176. | ORCHID | NCT04332991 | http://www.ncbi.nlm.nih.gov/pubmed/33165621 |
| Shenoy AG, Hettinger AZ, Fernandez SJ, Blumenthal J, Baez V (2021). Early mortality benefit with COVID-19 convalescent plasma: a matched control study.. Br J Haematol 192: 706-713. | Shenoy AG 2021 |  | http://www.ncbi.nlm.nih.gov/pubmed/33482025 |
| Simonovich VA, Burgos Pratx LD, Scibona P, Beruto MV, Vallone MG, Vazquez C, Savoy N, Giunta DH, Perez LG, Sanchez MDL, Gamarnik AV, Ojeda DS, Santoro DM, Camino PJ, Antelo S, Rainero K, Vidiella GP, Miyazaki EA, Cornistein W, Trabadelo OA, Ross FM, Spotti M, Funtowicz G, Scordo WE, Losso MH, Ferniot I, Pardo PE, Rodriguez E, Rucci P, Pasquali J, Fuentes NA, Esperatti M, Speroni GA, Nannini EC, Matteaccio A, Michelangelo HG, Follmann D, Lane HC, Belloso WH (2020). A Randomized Trial of Convalescent Plasma in Covid-19 Severe Pneumonia. N Engl J Med. | PLASM-AR | NCT04383535 | http://www.ncbi.nlm.nih.gov/pubmed/33232588 |
| Singh AK, Oks M, Husk G, Dechario SP, Mina B, Singh K, Kirschenbaum L, Carpati CM, Mahmoud O, Gabra NI, Ishikawa O, Altschultz E, Shah V, Mahajan A, Gautam A, Birnbaum B, Antonacci AC, Raoof S (2021). Impact of Timing of Tocilizumab Use in Hospitalized Patients With SARS-CoV-2 Infection. Respir Care 66: 1805-1814. | Singh AK 2021 |  | http://www.ncbi.nlm.nih.gov/pubmed/34548407 |
| Singh S, Khan A, Chowdhry M, Chatterjee A (2020). Outcomes of Hydroxychloroquine Treatment Among Hospitalized COVID-19 Patients in the United States- Real-World Evidence From a Federated Electronic Medical Record Network. medRxiv. | Singh S 2020 |  | http://doi.org/10.1101/2020.05.12.20099028 |
| Skipper CP, Pastick KA, Engen NW, Bangdiwala AS, Abassi M, Lofgren SM, Williams DA, Okafor EC, Pullen MF, Nicol MR, Nascene AA, Hullsiek KH, Cheng MP, Luke D, Lother SA, MacKenzie LJ, Drobot G, Kelly LE, Schwartz IS, Zarychanski R, McDonald EG, Lee TC, Rajasingham R, Boulware DR (2020). Hydroxychloroquine in Nonhospitalized Adults With Early COVID-19: A Randomized Trial. Ann Intern Med. | COVID-19 PEP | NCT04308668 | http://www.ncbi.nlm.nih.gov/pubmed/32673060 |
| Soin AS, Kumar K, Choudhary NS, Sharma P, Mehta Y, Kataria S, Govil D, Deswal V, Chaudhry D, Singh PK, Gupta A, Agarwal V, Kumar S, Sangle SA, Chawla R, Narreddy S, Pandit R, Mishra V, Goel M, Ramanan AV (2021). Tocilizumab plus standard care versus standard care in patients in India with moderate to severe COVID-19-associated cytokine release syndrome (COVINTOC): an open-label, multicentre, randomised, controlled, phase 3 trial. Lancet Respir Med S2213-2600. | COVINTOC | CTRI/2020/05/025369 | http://www.ncbi.nlm.nih.gov/pubmed/33676589 |
| Somers EC, Eschenauer GA, Troost JP, Golob JL, Gandhi TN, Wang L, Zhou N, Petty LA, Baang JH, Dillman NO, Frame D, Gregg KS, Kaul DR, Nagel J, Patel TS, Zhou S, Lauring AS, Hanauer DA, Martin E, Sharma P, Fung CM, Pogue JM (2020). Tocilizumab for treatment of mechanically ventilated patients with COVID-19. Clin Infect Dis. | Somers EC 2020 |  | http://www.ncbi.nlm.nih.gov/pubmed/32651997 |
| Soto-Becerra P, Culquichicon C, Hurtado-Roca Y, Araujo-Castillo RV (2020). Real-World Effectiveness of hydroxychloroquine, azithromycin, and ivermectin among hospitalized COVID-19 patients: Results of a target trial emulation using observational data from a nationwide Healthcare System in Peru. medRxiv. | Soto-Becerra P 2020 |  | http://doi.org/10.1101/2020.10.06.20208066 |
| Spagnuolo V, Guffanti M, Galli L, Poli A, Querini PR, Ripa M, Clementi M, Scarpellini P, Lazzarin A, Tresoldi M, Dagna L, Zangrillo A, Ciceri F, Castagna A (2020). Viral clearance after early corticosteroid treatment in patients with moderate or severe covid-19. Sci Rep 10: 21291. | Spagnuolo V 2020 | NCT04318366 | http://www.ncbi.nlm.nih.gov/pubmed/33277573 |
| Spinner CD, Gottlieb RL, Criner GJ, Lopez JRA, Cattelan AM, Viladomiu AS, Ogbuagu O, Malhotra P, Mullane KM, Castagna A, Chai LYA, Roestenberg M, Tsang OTY, Bernasconi E, Turnier PL, Chang SC, SenGupta D, Hyland RH, Osinusi AO, Cao H, Blair C, Wang H, Gaggar A, Brainard DM, McPhail MJ, Bhagani S, Ahn MY, Sanyal AJ, Huhn G, Marty FM (2020). Effect of Remdesivir vs Standard Care on Clinical Status at 11 Days in Patients With Moderate COVID-19: A Randomized Clinical Trial. JAMA. | GS-US-540-5774 | NCT04292730 | http://www.ncbi.nlm.nih.gov/pubmed/32821939 |
| Stone JH, Frigault MJ, Serling-Boyd NJ, Fernandes AD, Harvey L, Foulkes AS, Horick NK, Healy BC, Shah R, Bensaci AM, Woolley AE, Nikiforow S, Lin N, Sagar M, Schrager H, Huckins DS, Axelrod M, Pincus MD, Fleisher J, Sacks CA, Dougan M, North CM, Halvorsen YD, Thurber TK, Dagher Z, Scherer A, Wallwork RS, Kim AY, Schoenfeld S, Sen P, Neilan TG, Perugino CA, Unizony SH, Collier DS, Matza MA, Yinh JM, Bowman KA, Meyerowitz E, Zafar A, Drobni ZD, Bolster MB, Kohler M, D'Silva KM, Dau J, Lockwood MM, Cubbison C, Weber BN, Mansour MK (2020). Efficacy of Tocilizumab in Patients Hospitalized with Covid-19. N Engl J Med. | 2020P001159 | NCT04356937 | http://www.ncbi.nlm.nih.gov/pubmed/33085857 |
| Sulaiman T, Mohana A, Alawdah L, Mahmoud N, Hassanein M, Wani T, Alfaifi A, Alenazi E, Radwan N, Alkhalifah N, Elkady E, Alanazi M, Alqahtani M, Abdalla K, Yousif Y, AboGazalah F, Awwad F, AlabdulKareem K, AlGhofaili F, AlJedai A, Jokhdar H, Alrabiah F (2020). The Effect of Early Hydroxychloroquine-based Therapy in COVID-19 Patients in Ambulatory Care Settings: A Nationwide Prospective Cohort Study. medRxiv. | Sulaiman T 2020 |  | http://doi.org/10.1101/2020.09.09.20184143 |
| Sullivan DJ, Gebo KA, Shoham S, Bloch EM, Lau B, Shenoy AG, Mosnaim GS, Gniadek TJ, Fukuta Y, Patel B, Heath SL, Levine AC, Meisenberg BR, Spivak ES, Anjan S, Huaman MA, Blair JE, Currier JS, Paxton JH, Gerber JM, Petrini JR, Broderick PB, Rausch W, Cordisco ME, Hammel J, Greenblatt B, Cluzet VC, Cruser D, Oei K, Abinante M, Hammitt LL, Sutcliffe CG, Forthal DN, Zand MS, Cachay ER, Raval JS, Kassaye SG, Foster EC, Roth M, Marshall CE, Yarava A, Lane K, McBee NA, Gawad AL, Karlen N, Singh A, Ford DE, Jabs DA, Appel LJ, Shade DM, Ehrhardt S, Baksh SN, Laeyendecker O, Pekosz A, Klein SL, Casadevall A, Tobian AAR, Hanley DF (2021). Randomized Controlled Trial of Early Outpatient COVID-19 Treatment with High-Titer Convalescent Plasma. medRxiv. | CSSC-004 | NCT04373460 | https://doi.org/10.1101/2021.12.10.21267485 |
| Talaschian M, Akhtari M, Mahmoudi M, Mostafaei S, Jafary M, Husseini AS, Sadeghi K, Moghadam KG, Tadi HA, Jamshidi A (2021). Tocilizumab Failed to Reduce Mortality in Severe COVID-19 Patients: Results From a ?Randomized Controlled Clinical Trial. Research Square. | Talaschian M 2021 | IRCT20081027001411N4 | http://doi.org/10.21203/rs.3.rs-463921/v1 |
| Tang W, Cao Z, Han M, Wang Z, Chen J, Sun W, Wu Y, Xiao W, Liu S, Chen E, Chen W, Wang X, Yang J, Lin J, Zhao Q, Yan Y, Xie Z, Li D, Yang Y, Liu L, Qu J, Ning G, Shi G, Xie Q (2020). Hydroxychloroquine in patients with mainly mild to moderate coronavirus disease 2019: open label, randomised controlled trial. BMJ 369: m1849. | Tang W 2020 | ChiCTR2000029868 | http://www.ncbi.nlm.nih.gov/pubmed/32409561 |
| Tang X, Feng YM, Ni JX, Zhang JY, Liu LM, Hu K, Wu ZX, Zhang JX, Chen JW, Zhang JC, Su J, Li YL, Zhao Y, Xie J, Ding Z, He XL, Wang W, Jin RH, Shi HZ, Sun B (2021). Early Use of Corticosteroid May Prolong SARS-CoV-2 Shedding in Non-Intensive Care Unit Patients with COVID-19 Pneumonia: A Multicenter, Single-Blind, Randomized Control Trial. Respiration: 1-11. | Tang X 2021 | NCT04273321 | http://www.ncbi.nlm.nih.gov/pubmed/33486496 |
| Tomazini BM, Maia IS, Cavalcanti AB, Berwanger O, Rosa RG, Veiga VC, Avezum A, Lopes RD, Bueno FR, Silva MVAO, Baldassare FP, Costa ELV, Moura RAB, Honorato MO, Costa AN, Damiani LP, Lisboa T, Kawano-Dourado L, Zampieri FG, Olivato GB, Righy C, Amendola CP, Roepke RML, Freitas DHM, Forte DN, Freitas FGR, Fernandes CCF, Melro LMG, Gedealvares FS Junior, Morais DC, Zung S, Machado FR, Azevedo LCP (2020). Effect of Dexamethasone on Days Alive and Ventilator-Free in Patients With Moderate or Severe Acute Respiratory Distress Syndrome and COVID-19: The CoDEX Randomized Clinical Trial. JAMA. | CoDEX | NCT04327401 | http://www.ncbi.nlm.nih.gov/pubmed/32876695 |
| Tran VT, Mahevas M, Bani-Sadr F, Robineau O, Perpoint T, Perrodeau E, Gallay L, Ravaud P, Goehringer F, Lescure FX (2020). Association between corticosteroids and intubation or death among patients with COVID-19 pneumonia in non-ICU settings: an observational study using of real-world data from 51 hospitals in France and Luxembourg. Clin Microbiol Infect. | COCORICO |  | http://www.ncbi.nlm.nih.gov/pubmed/33301928 |
| Tsai A, Diawara O, Nahass RG, Brunetti L (2020). Impact of tocilizumab administration on mortality in severe COVID-19. Sci Rep 10: 19131. | Tsai A 2020 |  | http://www.ncbi.nlm.nih.gov/pubmed/33154452 |
| Tworek A, Jaron K, Uszynska-Kaluza B, Rydzewski A, Gil R, Deptala A, Franek E, Wojtowicz R, Zycinska K, Walecka I, Cicha M, Wierzba W, Zaczynski A, Krol ZJ, Rydzewska G (2021). Convalescent plasma treatment is associated with lower mortality and better outcomes in high risk COVID-19 patients - propensity score matched case-control study. Int J Infect Dis 105: 209-215. | Tworek A 2021 |  | http://www.ncbi.nlm.nih.gov/pubmed/33607305 |
| Ulrich RJ, Troxel AB, Carmody E, Eapen J, Backer M, DeHovitz JA, Prasad PJ, Li Y, Delgado C, Jrada M, Robbins GA, Henderson B, Hrycko A, Delpachitra D, Raabe V, Austrian JS, Dubrovskaya Y, Mulligan MJ (2020). Treating COVID-19 With Hydroxychloroquine (TEACH): A Multicenter, Double-Blind Randomized Controlled Trial in Hospitalized Patients. Open Forum Infect Dis 7: ofaa446. | TEACH | NCT04369742 | http://www.ncbi.nlm.nih.gov/pubmed/33134417 |
| UNICEF (2020). Clearing the Fog: Is HCQ Effective in Reducing COVID-19 progression-a Randomized Controlled Trial. clintrials.gov. | Sultan Mehmood Kamran 2 | NCT04491994 | http://clinicaltrials.gov/show/NCT04491994 |
| Veiga VC, Prats JAGG, Farias DLC, Rosa RG, Dourado LK, Zampieri FG, Machado FR, Lopes RD, Berwanger O, Azevedo LCP, Avezum A, Lisboa TC, Rojas SSO, Coelho JC, Leite RT, Carvalho JC, Andrade LEC, Sandes AF, Pintao MCT, Castro CG Jr, Santos SV, de Almeida TML, Costa AN, Gebara OCE, de Freitas FGR, Pacheco ES, Machado DJB, Martin J, Conceicao FG, Siqueira SRR, Damiani LP, Ishihara LM, Schneider D, de Souza D, Cavalcanti AB, Scheinberg P (2021). Effect of tocilizumab on clinical outcomes at 15 days in patients with severe or critical coronavirus disease 2019: randomised controlled trial. BMJ 372: n84. | TOCIBRAS | NCT04403685 | http://www.ncbi.nlm.nih.gov/pubmed/33472855 |
| Wadud N, Ahmed N, Mannu SM, Khan M, Krishna MG, Gilani A, El ZS, Galaydick J, Linga K, Koor S, Galea J, Stuczynski L, Osundele MB (2020). Improved survival outcome in SARs-CoV-2 (COVID-19) Acute Respiratory Distress Syndrome patients with Tocilizumab administration. medRxiv. | Wadud N 2020 |  | http://doi.org/10.1101/2020.05.13.20100081 |
| Wang Y, Jiang W, He Q, Wang C, Wang B, Zhou P, Dong N, Tong Q (2020). A retrospective cohort study of methylprednisolone therapy in severe patients with COVID-19 pneumonia. Signal Transduct Target Ther 5: 57. | Wang Y 2020 |  | http://www.ncbi.nlm.nih.gov/pubmed/32341331 |
| Wang Y, Zhang D, Du G, Du R, Zhao J, Jin Y, Fu S, Gao L, Cheng Z, Lu Q, Hu Y, Luo G, Wang K, Lu Y, Li H, Wang S, Ruan S, Yang C, Mei C, Wang Y, Ding D, Wu F, Tang X, Ye X, Ye Y, Liu B, Yang J, Yin W, Wang A, Fan G, Zhou F, Liu Z, Gu X, Xu J, Shang L, Zhang Y, Cao L, Guo T, Wan Y, Qin H, Jiang Y, Jaki T, Hayden FG, Horby PW, Cao B, Wang C (2020). Remdesivir in adults with severe COVID-19: a randomised, double-blind, placebo-controlled, multicentre trial. Lancet 395: 1569-1578. | CAP-China remdesivir 2 | NCT04257656 | https://doi.org/10.1016/S0140-6736(20)31022-9 |
| Wu C, Hou D, Du C, Cai Y, Zheng J, Xu J, Chen X, Chen C, Hu X, Zhang Y, Song J, Wang L, Chao YC, Feng Y, Xiong W, Chen D, Zhong M, Hu J, Jiang J, Bai C, Zhou X, Xu J, Song Y, Gong F (2020). Corticosteroid therapy for coronavirus disease 2019-related acute respiratory distress syndrome: a cohort study with propensity score analysis. Crit Care 24: 643. | Wu C 2020 |  | https://www.ncbi.nlm.nih.gov/pubmed/33172477 |
| Wu J, Huang J, Zhu G, Liu Y, Xiao H, Zhou Q, Si X, Yi H, Wang C, Yang D, Chen S, Liu X, Liu Z, Wang Q, Lv Q, Huang Y, Yu Y, Guan X, Li Y, Nirantharakumar K, Cheng K, Peng S, Xiao H (2020). Systemic corticosteroids and mortality in severe and critical COVID-19 patients in Wuhan, China. J Clin Endocrinol Metab 105. | Wu J 2020 |  | http://www.ncbi.nlm.nih.gov/pubmed/32880390 |
| Xia X, Li K, Wu L, Wang Z, Zhu M, Huang B, Li J, Wang Z, Wu W, Wu M, Li W, Li L, Cai Y, Bosco B, Zhong A, Liu X, Lv T, Gan Z, Chen G, Pan Y, Liu C, Zhang K, Xu X, Wang C, Wang Q (2020). Improved clinical symptoms and mortality among patients with severe or critical COVID-19 after convalescent plasma transfusion. Blood 136: 755-759. | Xia X 2020 |  | http://www.ncbi.nlm.nih.gov/pubmed/32573724 |
| Yang R, Xiong Y, Ke H, Chen T, Gao S (2020). The Role of Methylprednisolone on Preventing Disease Progression for Hospitalized Patients with Severe COVID-19. Eur J Clin Invest 50: e13412. | Yang R 2020 |  | http://www.ncbi.nlm.nih.gov/pubmed/32954492 |
| Yoon HA, Bartash R, Gendlina I, Rivera J, Nakouzi A, Bortz RH, Wirchnianski AS, Paroder M, Fehn K, Serrano-Rahman L, Babb R, Sarwar UN, Haslwanter D, Laudermilch E, Florez C, Dieterle ME, Jangra RK, Fels JM, Tong K, Mariano MC, Vergnolle O, Georgiev GI, Herrera NG, Malonis RJ, Quiroz JA, Morano NC, Krause GJ, Sweeney JM, Cowman K, Allen S, Annam J, Applebaum A, Barboto D, Khokhar A, Lally BJ, Lee A, Lee M, Malaviya A, Sample R, Yang XA, Li Y, Ruiz R, Thota R, Barnhill J, Goldstein DY, Uehlinger J, Garforth SJ, Almo SC, Lai JR, Gil MR, Fox AS, Chandran K, Wang T, Daily JP, Pirofski LA (2020). Treatment of Severe COVID-19 with Convalescent Plasma in the Bronx, NYC. medRxiv. | Yoon HA 2020 |  | https://doi.org/10.1101/2020.12.02.20242909 |
| Yu B, Wang DW, Li C (2020). Hydroxychloroquine application is associated with a decreased mortality in critically ill patients with COVID-19. medRxiv. | Yu B 2020 |  | https://www.medrxiv.org/content/10.1101/2020.04.27.20073379v1 |
| Zarebska-Michaluk D, Jaroszewicz J, Rogalska M, Martonik D, Pabjan P, Berkan-Kawinska A, Bolewska B, Oczko-Grzesik B, Kozielewicz D, Tudrujek-Zdunek M, Kowalska J, Moniuszko-Malinowska A, K?os K, Rorat M, Leszczynski P, Piekarska A, Polanska J, Flisiak R (2021). Effectiveness of Tocilizumab with and without Dexamethasone in Patients with Severe COVID-19: A Retrospective Study. J Inflamm Res 14: 3359-3366. | Zarebska-Michaluk D 2021 |  | http://www.ncbi.nlm.nih.gov/pubmed/34295173 |
| Zeng QL, Yu ZJ, Gou JJ, Li GM, Ma SH, Zhang GF, Xu JH, Lin WB, Cui GL, Zhang MM, Li C, Wang ZS, Zhang ZH, Liu ZS (2020). Effect of Convalescent Plasma Therapy on Viral Shedding and Survival in COVID-19 Patients. J Infect Dis. | Zeng QL 2020 |  | http://www.ncbi.nlm.nih.gov/pubmed/32348485 |
| Zha L, Li S, Pan L, Tefsen B, Li Y, French N, Chen L, Yang G, Villanueva EV (2020). Corticosteroid Treatment of Patients With Coronavirus Disease 2019 (COVID-19). Med J Aust 212: 416-420. | Zha L 2020 |  | https://pubmed.ncbi.nlm.nih.gov/32266987 |
| Zheng KL, Xu Y, Guo YF, Diao L, Kong XY, Wan XJ, Zhao F, Ning FZ, Wang LB, Qiao F, Zhao JM, Zhou JH, Zhong YQ, Wu SX, Chen Y, Jin G, Dong YC (2020). Efficacy and safety of tocilizumab in COVID-19 patients. Aging (Albany NY) 12: 18878-18888. | Zheng KL 2020 |  | http://www.ncbi.nlm.nih.gov/pubmed/33031060 |

### Exploratory meta-analysis by treatment and study type, including study details

The following graphs provide an overview of observed odds-ratio for mortality for each included study by treatment and study type. The graphs show additional information on publication day since pandemic start (Day), Dose (low/medium/high), time in days since index for endpoint observation (time), age in years (Age), statistical analysis as a crude mean, IPTW, MV, or PS matched cohort (stat), quality of RWS with regards to confounding (Tier 1/2/3) and immortal time bias (Tier 1/2/3), and quality of RCT (Tier 1/2/3). The colour coding for the observed OR is by worst Tier for RWS. The graphs also show a mean estimate of treatment effect by treatment and study type from a random effects meta-analysis.

Figure S6: Exploratory meta-analysis and study detail for azithromycin in RWS


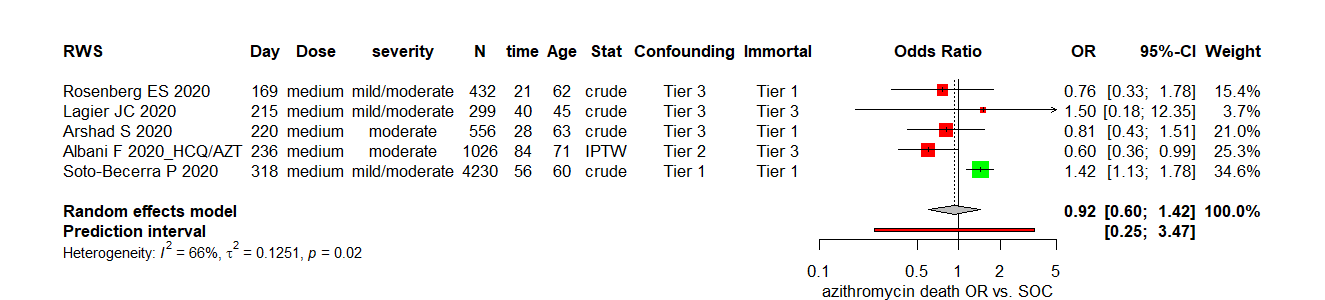


Figure S7: Exploratory meta-analysis and study detail for azithromycin in RCTs


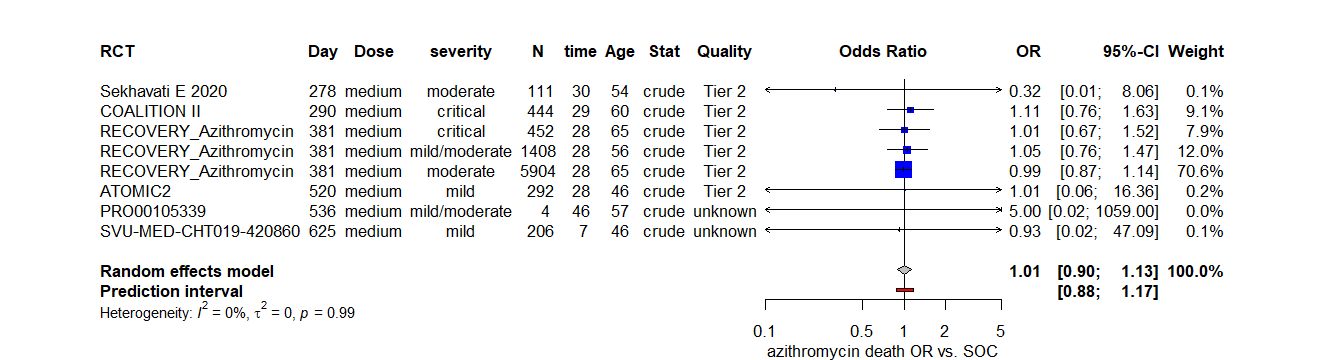


Figure S8: Exploratory meta-analysis and study detail for convalescent plasma in RWS


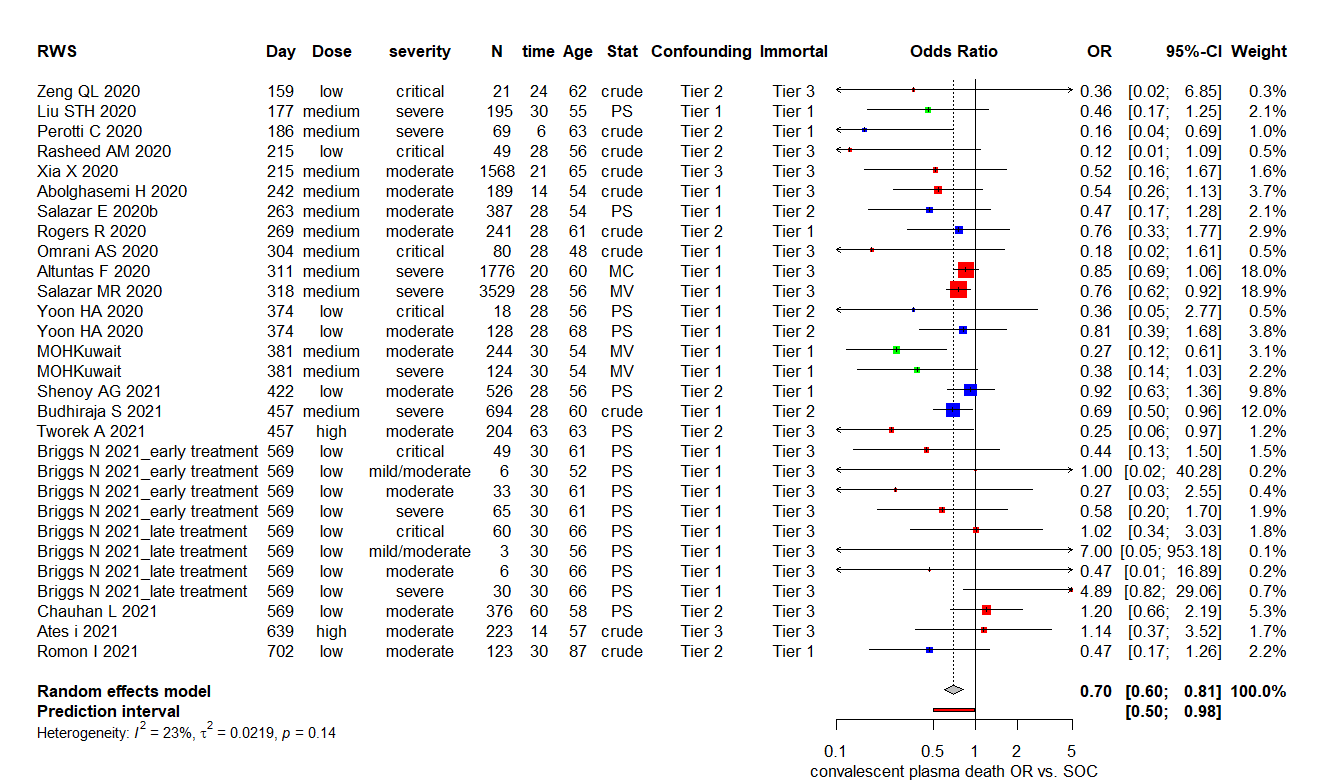


Figure S9: Exploratory meta-analysis and study detail for convalescent plasma in RCT


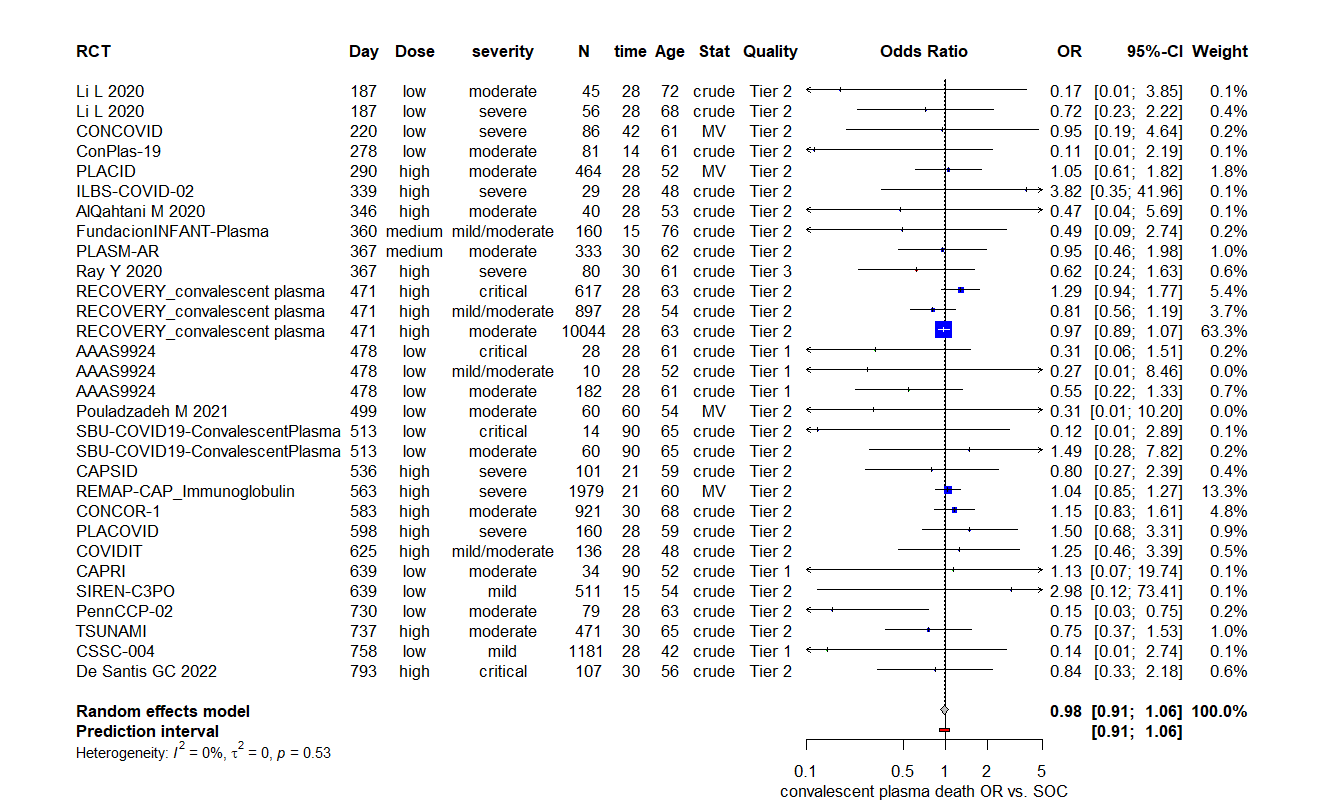


Figure S10: Exploratory meta-analysis and study detail for glucocorticoids in RWS


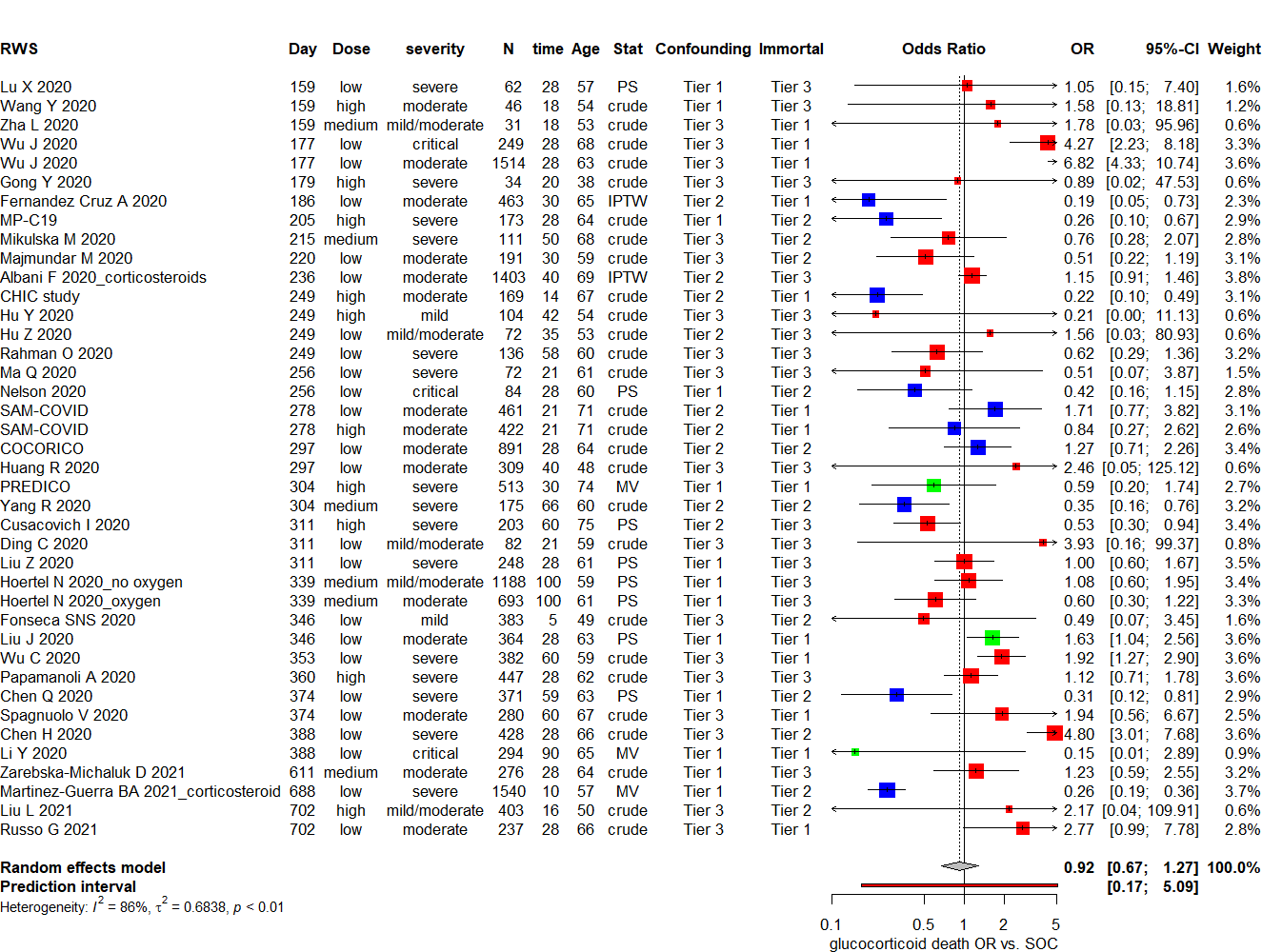


Figure S11: Exploratory meta-analysis and study detail for glucocorticoids in RCTs


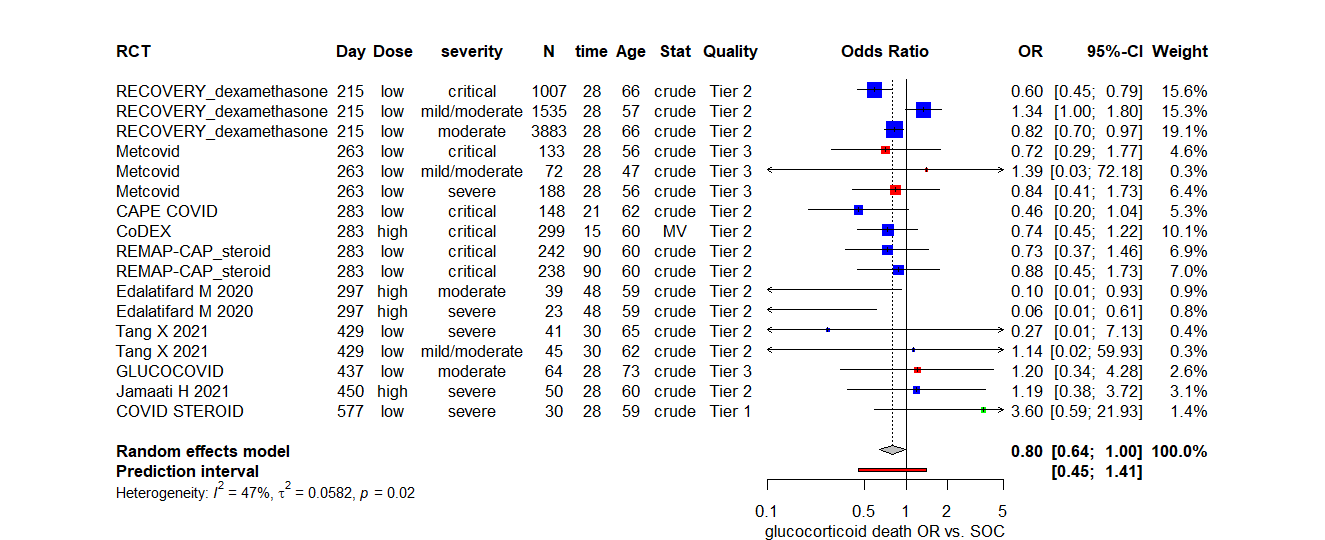


Figure S12: Exploratory meta-analysis and study detail for hydroxychloroquine in RWS


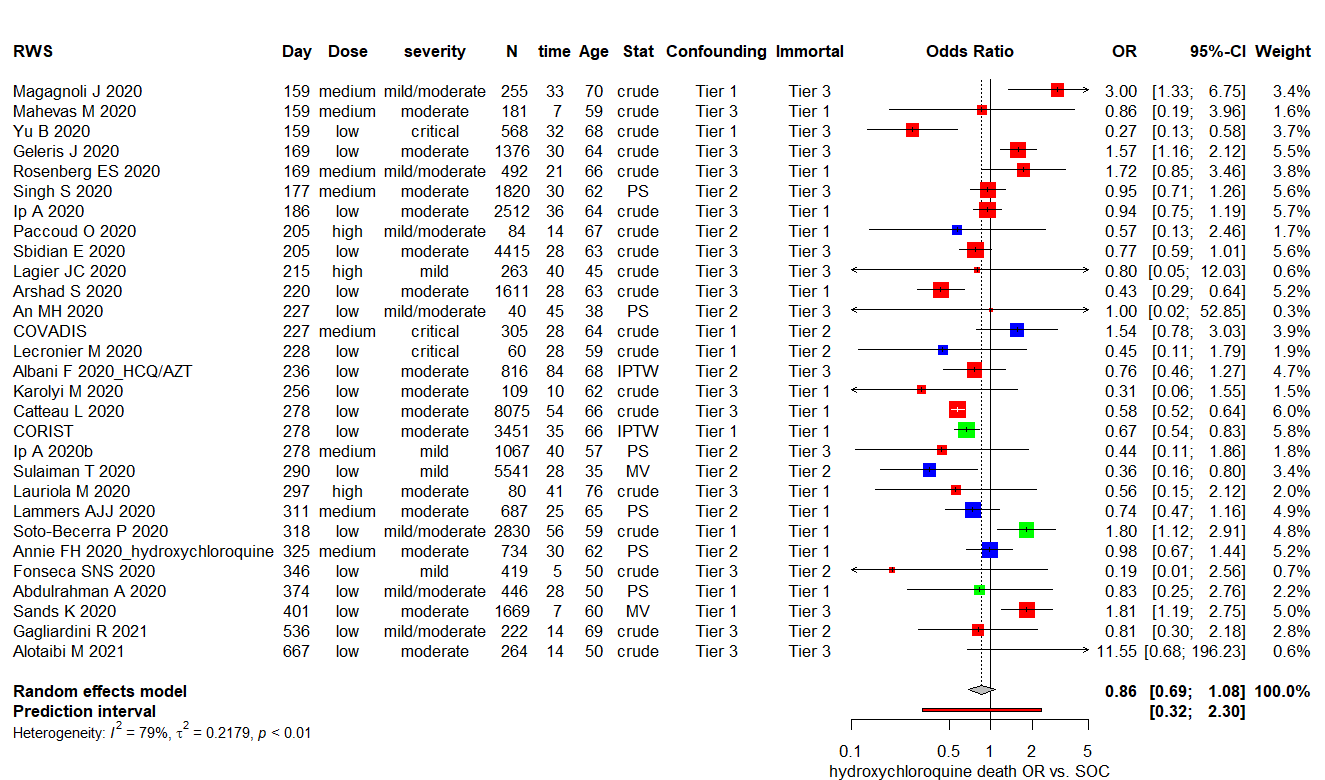


Figure S13: Exploratory meta-analysis and study detail for hydroxychloroquine in RCTs


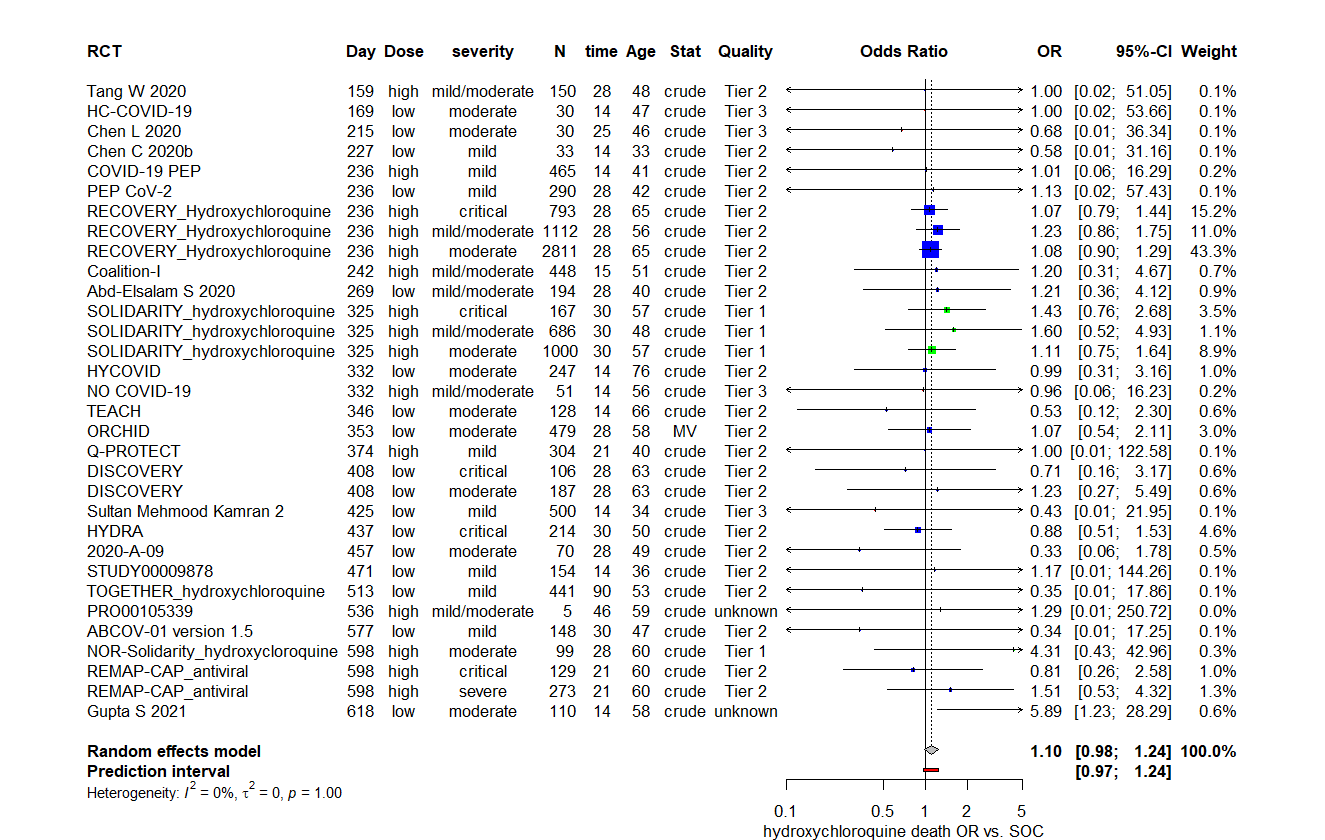


Figure S14: Exploratory meta-analysis and study detail for hydroxychloroquine plus azithromycin in RWS


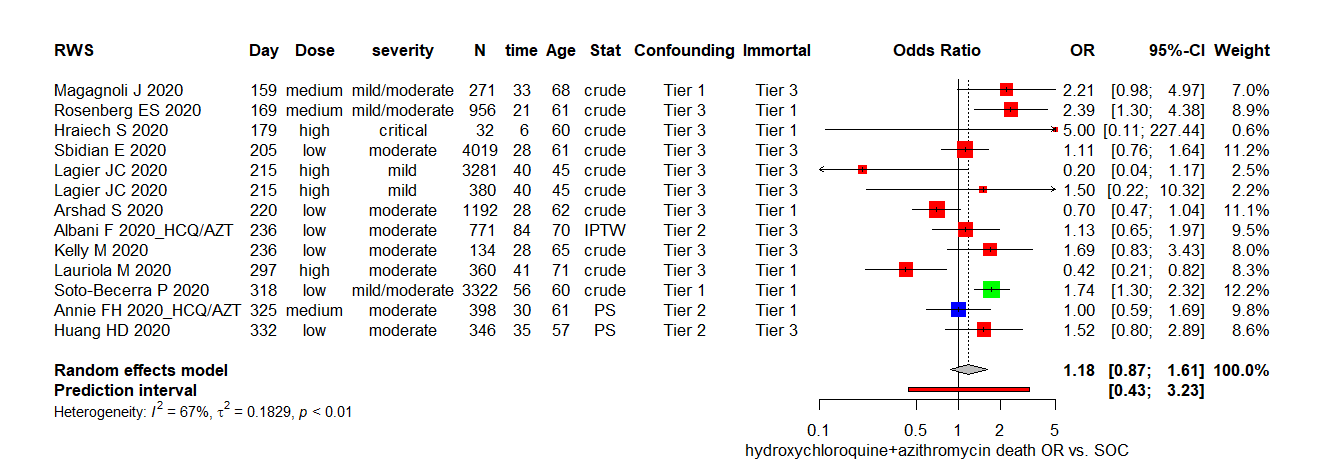


Figure S15: Exploratory meta-analysis and study detail for hydroxychloroquine plus azithromycin in RCTs


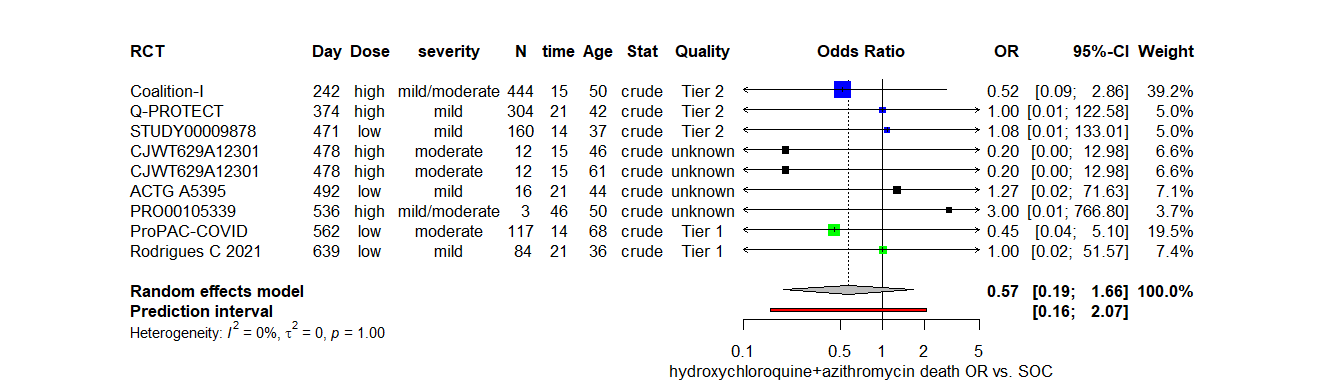


Figure S16: Exploratory meta-analysis and study detail for lopinavir in RWS


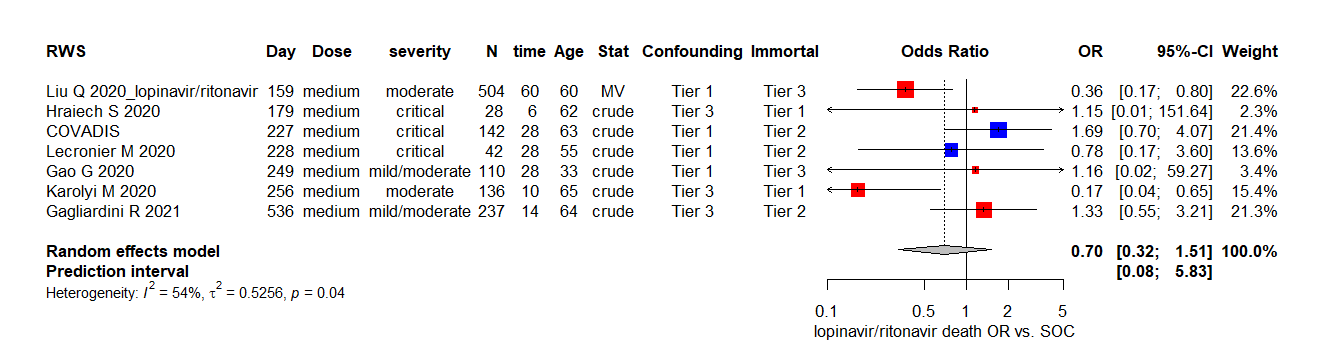


Figure S17: Exploratory meta-analysis and study detail for lopinavir in RCTs


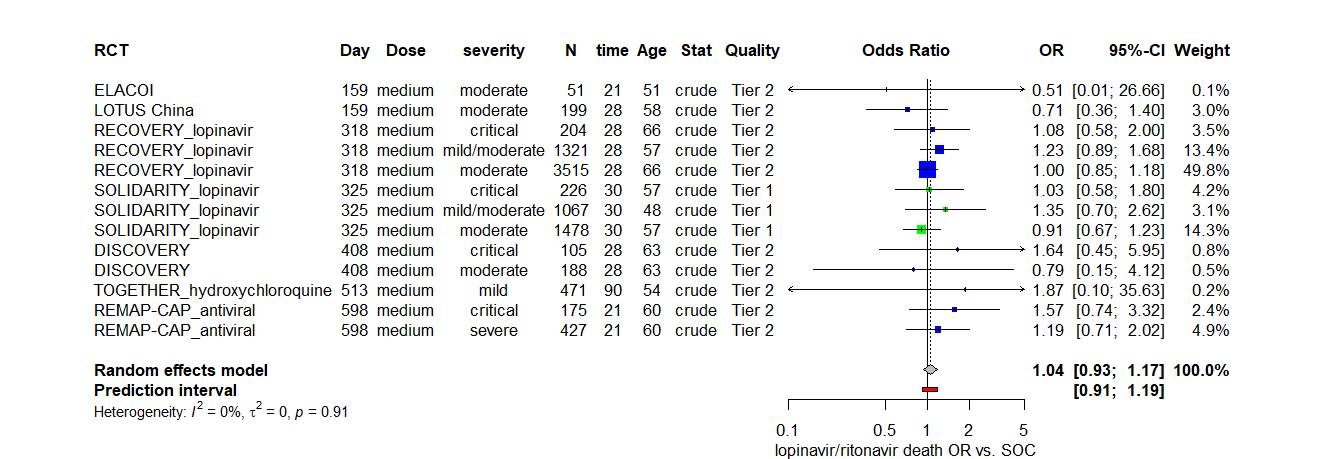


Figure S18: Exploratory meta-analysis and study detail for remdesivir in RWS


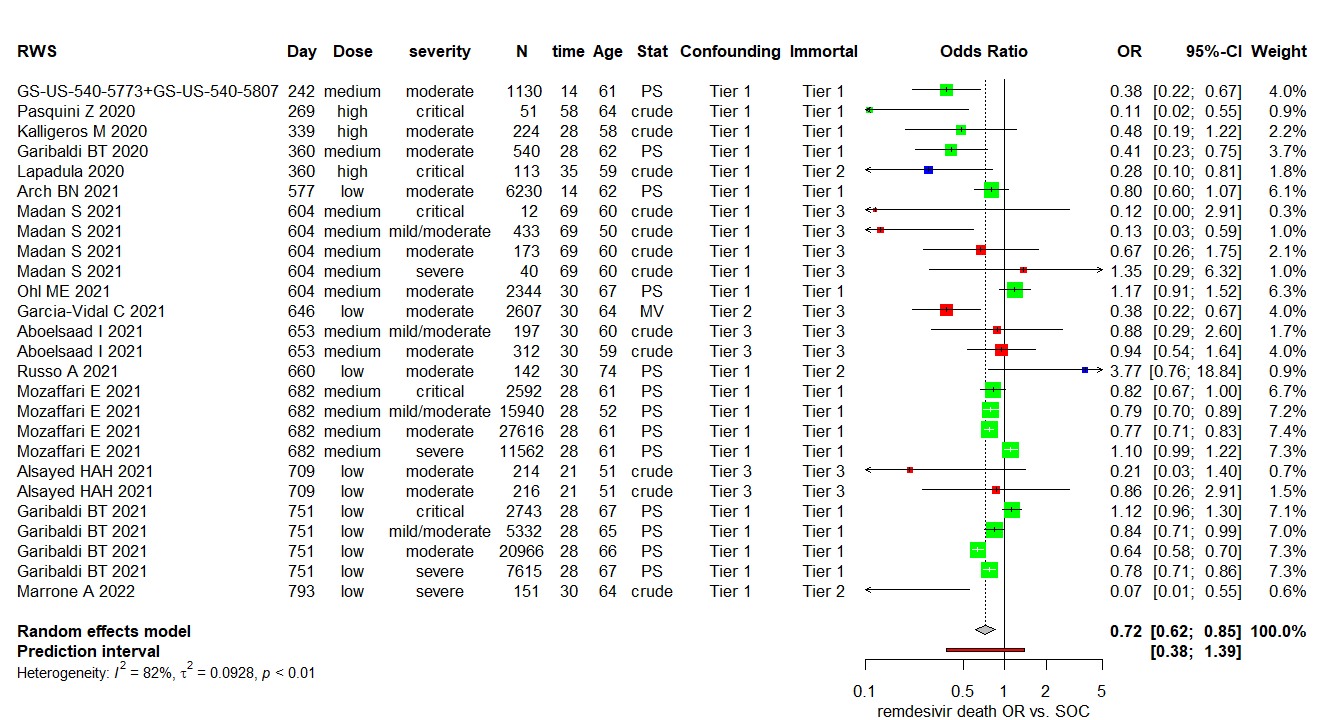


Figure S19: Exploratory meta-analysis and study detail for remdesivir in RCTs


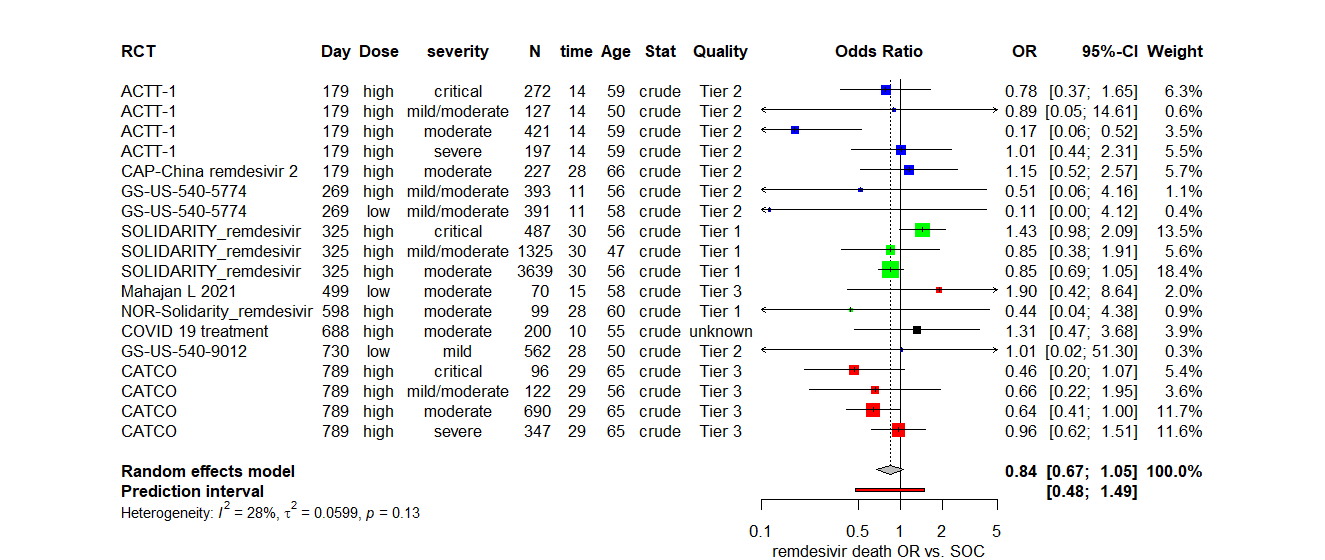


Figure S20: Exploratory meta-analysis and study detail for tocilizumab in RWS


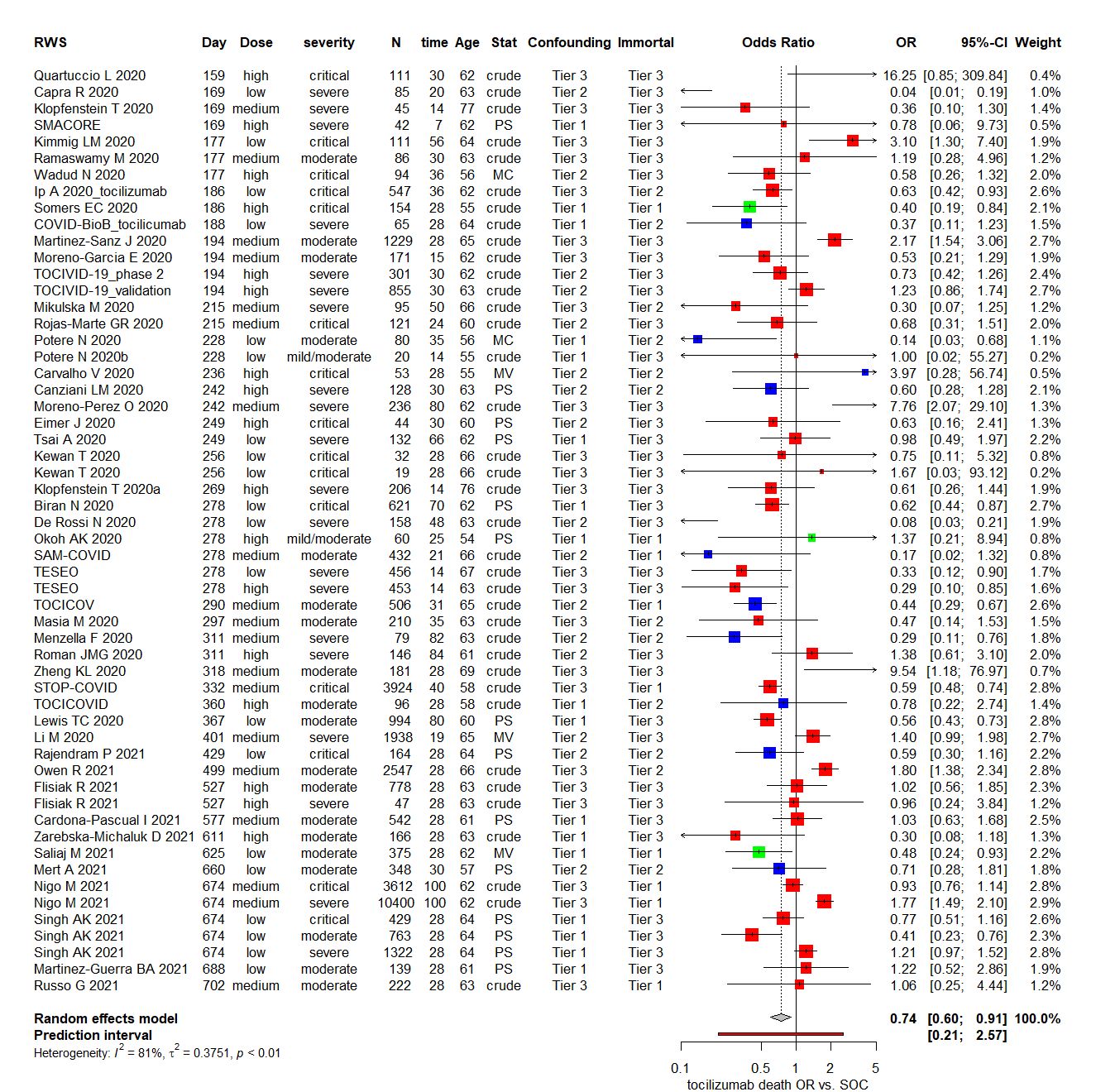


Figure S21: Exploratory meta-analysis and study detail for tocilizumab in RCTs


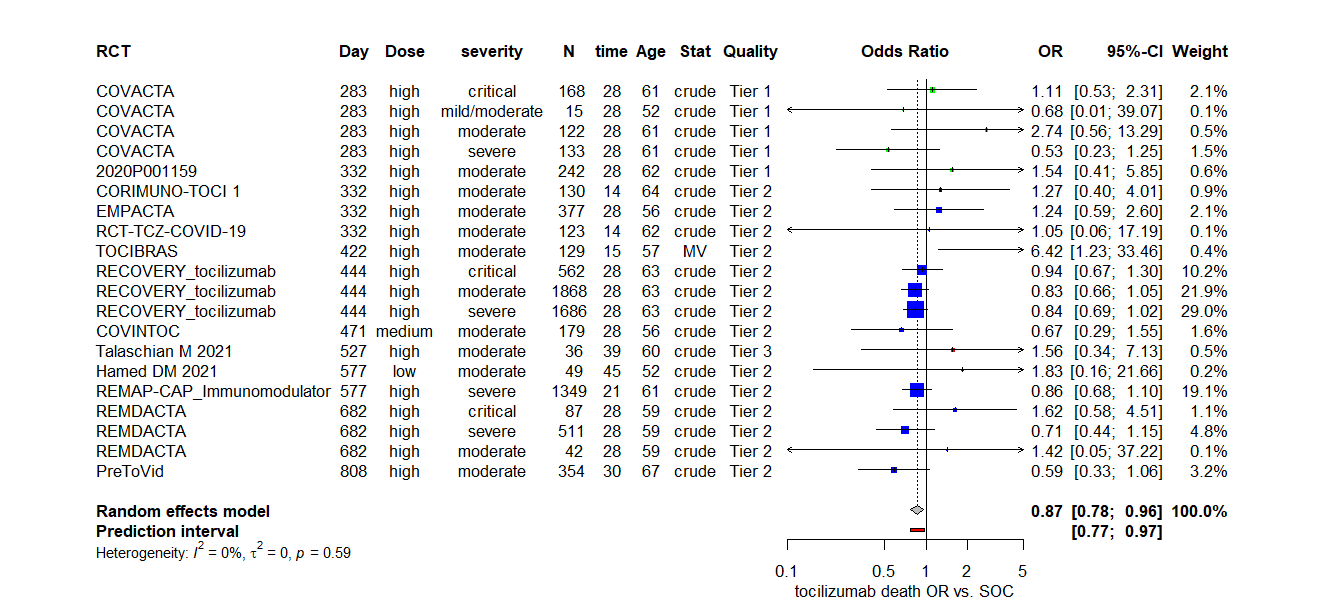


## Supplement E: Statistical Methods

A random effects meta-regression model was used to analyse the odds-ratio for mortality. The model for the log-odds ratio is:

$y_{i}\sim N\left( \theta_{i}, \sigma_{i}^{2} \right), \sigma_{i}^{2} is known$ Equation 1

where $i$ is the index for each study/treatment/severity combination reported across the studies. $y_{i}$ is the log-odds ratio for death, and $\sigma_{i}$ is the standard error of the log-odds ratio. The standard error was adjusted by the approximate variance inflation factor $l/2$ where $l$ is the number of treatment arms in the study including control.

The expected log-odds ratio is:

$\theta_{i}=X_{i}\beta+u_{i}$ Equation 2

$u_{i}\sim N(0,\tau_{k}^{2})$ Equation 3

where $X_{i}$ is a vector of covariates that specifies the treatment/severity combination and the study-level factors outlined in **Supplement C: Study extraction variables**. The $u_{i}$ are study/treatment/severity-level random effects that represent residual heterogeneity with standard deviation $\tau_{k}^{2}$. The base model estimated the variance of the random effects by study/treatment/severity combination. Nested random effects structures for disease severity subgroup within a study or treatment within a disease severity subgroup within study (in case multiple treatments are evaluated) were added if significant. Treatment arms within platform trials and RWS with multiple treatment arms that used a different control cohort were treated as separate studies.

The impact of study level covariates on the variance of the random effects, $\tau_{k}^{2},$ was evaluated. For example, the variance could differ by study type $k$, (i.e. RWS/RCT) and may be smaller for RCTs compared to RWS due to RCTs attempting to reduce variation as a design goal in many cases.

In a likelihood-based inference framework, the parameters to be estimated are the vector fixed effects $\beta$, and the random effects standard deviations $\tau_{1..n}$.

The impact of covariates ($X_{i}$) such as population and trial characteristics on the treatment effect was evaluated. The covariates were first assessed for a global impact on treatment effect. A global effect assumes that all treatments were impacted similarly by the covariate values across all study types. Secondly, a study type by covariate interaction was estimated to determine whether the covariate impacts the treatment effect differently for RWS vs. RCTs. Thirdly, the impact of covariates on the variance of between-study random effects was evaluated. Covariate effects were included in the model if statistically significant. For categorical covariates, a different impact was estimated for each level of the covariate. Covariate levels were combined if they were adjacent and shared a similar impact on treatment effect. For continuous covariates a linear and log-linear relationship between covariate and treatment effect were evaluated. Continuous covariates were also split into 5, 4, and 3 groups dependent on the percentiles of the available data for the covariate and evaluated as a categorical covariate. Each covariate included in the model was assessed again for its significance, after model building was complete.

Study type is the primary covariate of interest and quantifies the difference in estimated treatment effect between RWS and RCTs. The impact of this covariate was evaluated first. Disease severity was evaluated next because it was the patient characteristic most likely to impact treatment effect. For both of these covariates a full treatment by covariate interaction was estimated to determine if certain treatments were impacted differently by study type or disease severity. The other covariates were explored on basis of their perceived relevance, starting with time point of mortality observation, followed by quality characteristics of RWS (confounding tier, immortal tier, worst tier, and combo tier), quality characteristics of RCTs (risk of bias tier, blinding), quality characteristics for both RCT and RWS (study publication status, sample size, study region), timing of study publication, study start, and study midpoint relative to start of pandemic and relative to RECOVERY or SOLIDARITY publication for each treatment, dose, and imbalance in patient characteristics between the treated and control groups (age, sex, disease severity, comorbidities).

Model selection with regards to the impact of covariates was guided by a Log Likelihood ratio test and the confidence interval (CI) of the parameter estimate at an acceptance p-value of 0.05. The difference in -2 times the Log of the Likelihood (-2LL) between a full and reduced model is approximately asymptotically χ2 distributed with degrees of freedom equal to the difference in number of parameters between the two models. The standard error of the parameter estimate for covariate relationships was considered as well. Standard errors and correlation of the parameter estimates were approximated using the asymptotic variance-covariance matrix.

The generalized least squares regression function (gnls) and the nonlinear mixed effect regression function (nlme) provided in R (version 4.0.5 or later) were used to estimate maximum likelihood estimates of the model parameters. The meta (version 5.0-1) and metafor (version 3.0-2) package were used for exploratory analyses.
