# Supplementary material for: Totality of evidence of the effectiveness of repurposed therapies for COVID‐19: Can we use real‐world studies alongside randomized controlled trials?
Source: Clin Transl Sci. 2023 Aug 28;16(10):1842–55. doi: 10.1111/cts.13591 (PMC10582658; doi:10.1111/cts.13591)
Supplement: Supplementary file 2 — Appendix S2 [file CTS-16-1842-s001.docx]

**Supplementary Material**

**Totality of Evidence of the effectiveness of repurposed therapies for COVID-19: Can we use Real-World Studies alongside Randomised Controlled Trials?**

Short Title

Role of RWS and RCTs in Evidence Generation: COVID19 Therapeutics

# Supplements F-G

## Supplement F: Multivariable Mortality Analysis

Table S221: Explanatory factor estimates associated with statistically significant impact on treatment estimates- all others were non-significant

| **Factors^1^ Contributing to the Multivariable Regression Model** | **Point Estimate and [95% CI]** |
| --- | --- |
| **Global Effects** | |
| **RWS vs. RCT** | **0·84 [0·74 to 0·96]** |
| **standard deviation of the RWS between-study variability (**τ) | **0·37 [0·31 to 0·45]** |
|  |  |
| **RWS Effects** | |
| RWS week 1-2 vs. >=week 3 | 0·70 [0·52 to 0·93] |
| RWS confounding Tier 3 vs. Tier 1 and 2 | 1·51 [1·22 to 1·87] |
| RWS sample size < 200 vs. >=200 | 0·81 [0·66 to 0·99] |
| RWS $\Delta$ age (active – control) /year | 1·13 [1·09 to 1·16] |
| RWS $\Delta$ % males (active – control) | 1·03 [1·01 to 1·04] |
| RWS $\Delta$ % critical (active – control) | 1·04 [1·01 to 1·06] |

1) Factors listed have significant effect (P<0.05) in the multivariable model. All estimates of Treatment by disease severity from the model are presented in figure 3.
2) τ is expressed on the log scale. All other estimates are presented as an odds-ratio (OR)
Acronyms: RCT = randomised-controlled trial; RWS = Real-world studies

## Supplement G: Time course of evidence accumulation for individual treatments

Figure S23. Cumulative evidence of azithromycin treatment effect on mortality


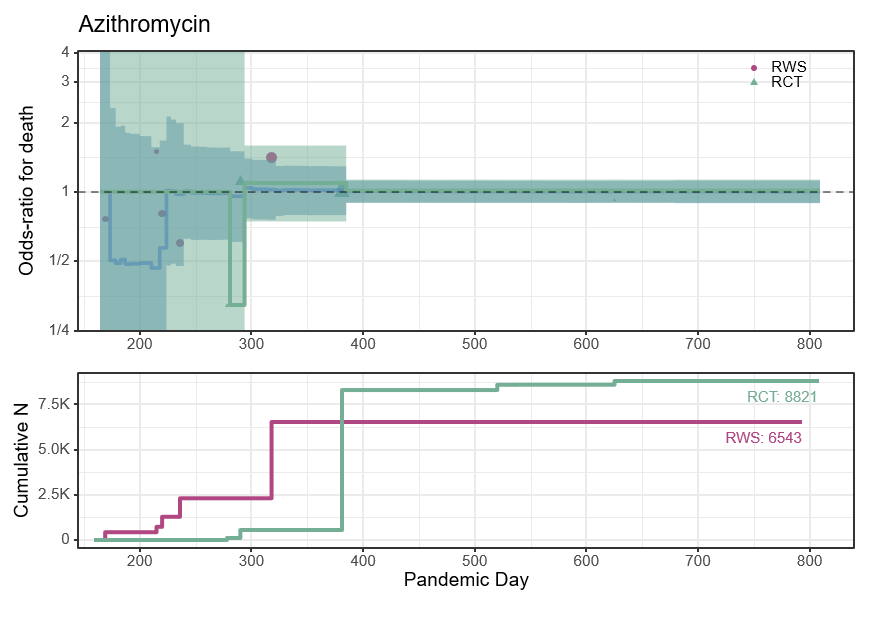


Figure S25. Cumulative evidence of hydroxychloroquine treatment effect on mortality


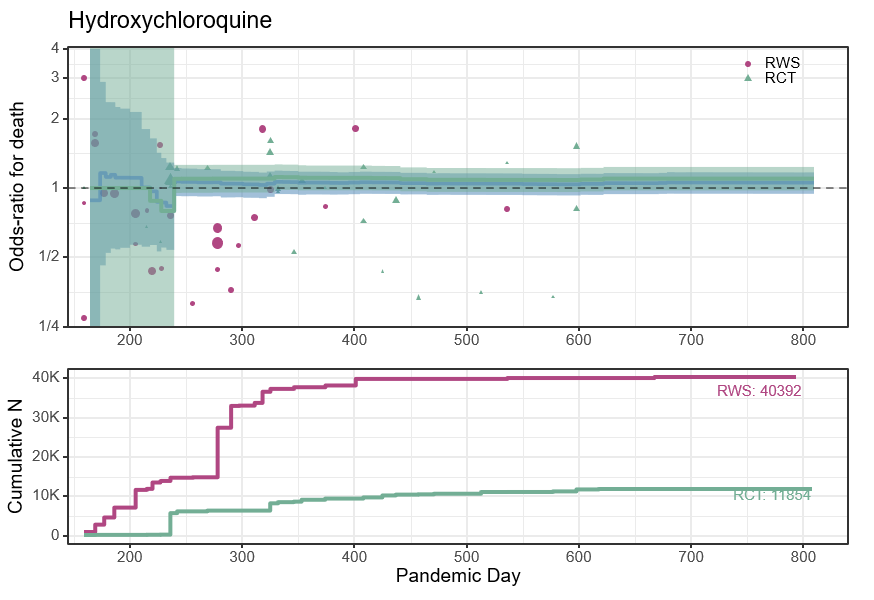


Figure S28. Cumulative evidence of hydroxychloroquine + azithromycin treatment effect on mortality


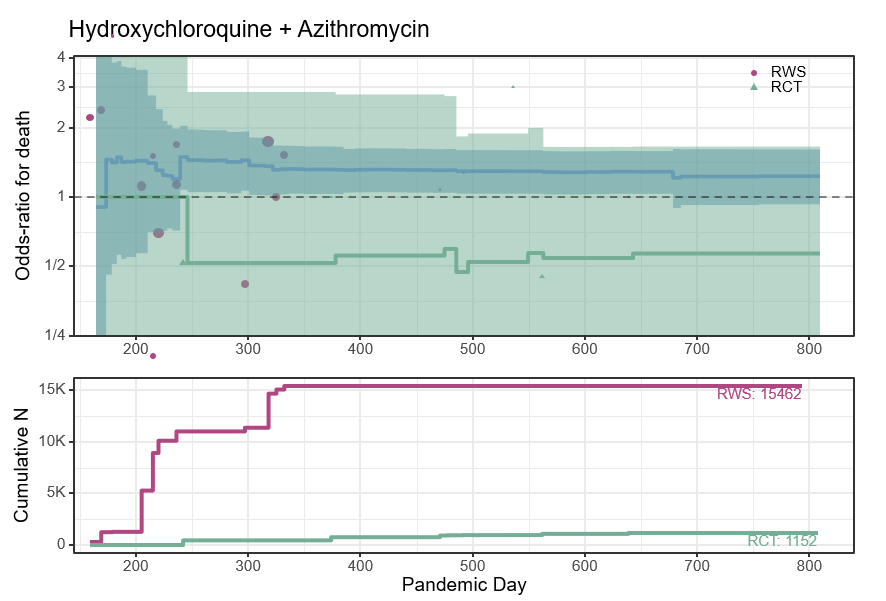


Figure S29. Cumulative evidence of lopinavir/ritonavir treatment effect on mortality


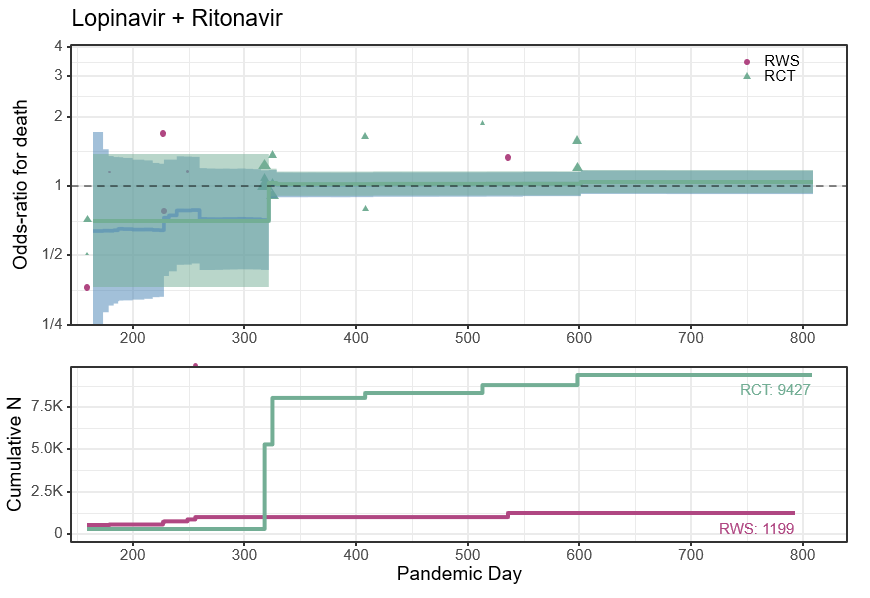


Figure S30. Cumulative evidence of remdesivir treatment effect on mortality for non-severe or critical patients


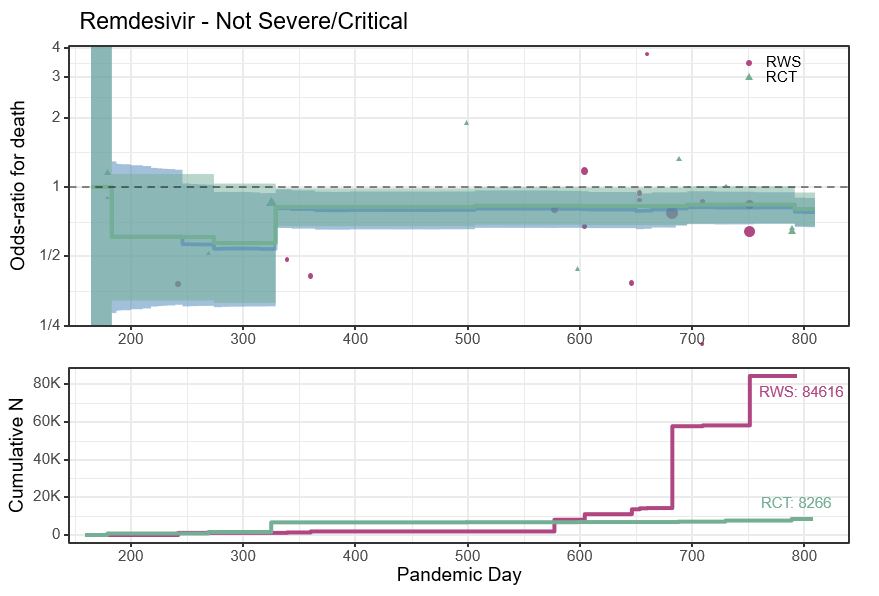


Figure S31. Cumulative evidence of remdesivir treatment effect on mortality for severe or critical patients

*
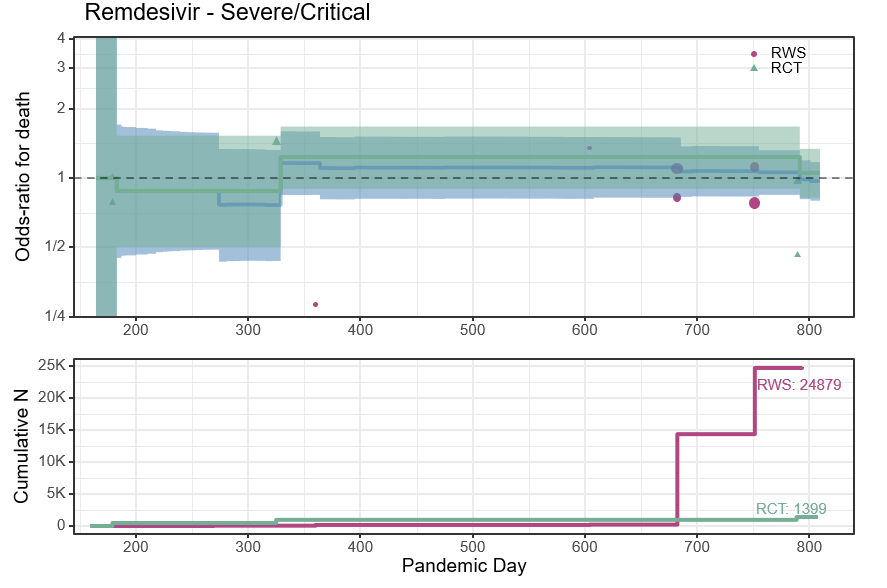
*

Figure S32. Cumulative evidence of glucocorticoid treatment effect on mortality for mild/moderate patients


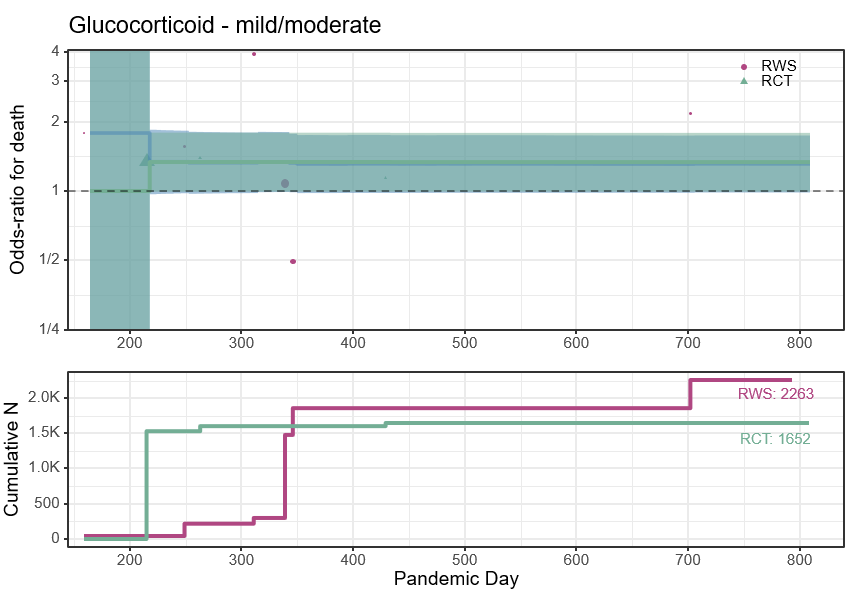


Figure S33. Cumulative evidence of glucocorticoid treatment effect on mortality for moderate patients


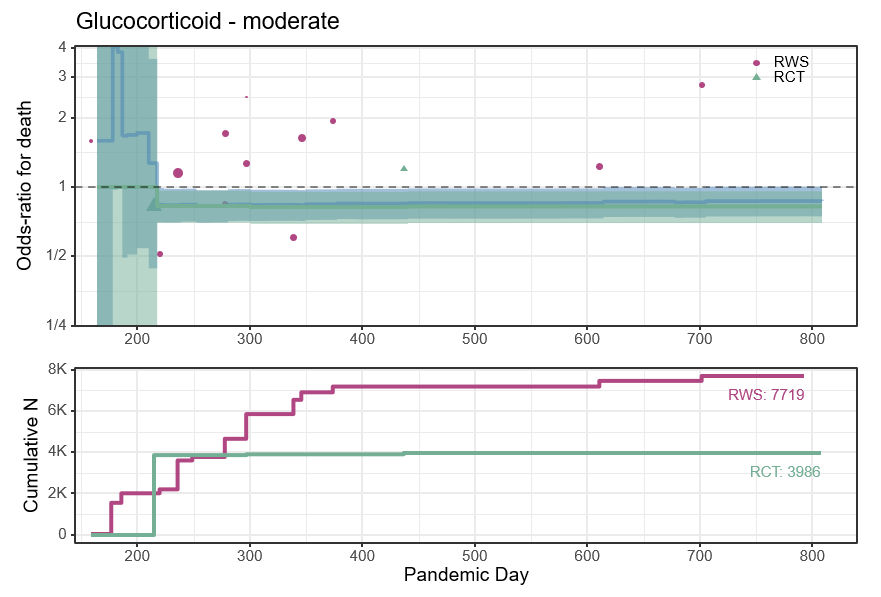


Figure S14. Cumulative evidence of glucocorticoid treatment effect on mortality for severe/critical patients


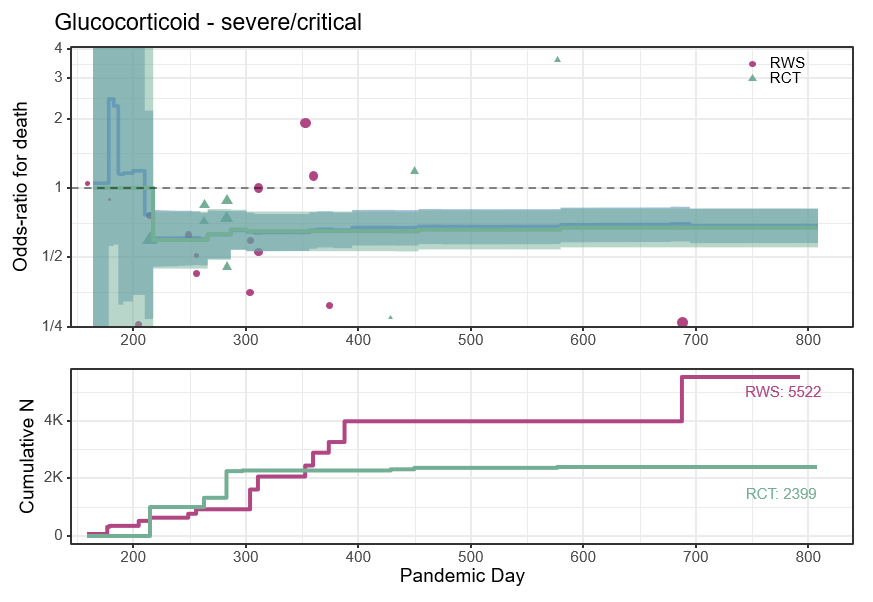


Figure S34. Probability of beneficial treatment effect (green line; odds-ratio<1) and no clinical benefit (red line; odds-ratio >0.9) as a function of day since start of pandemic based on RWS and RCTs


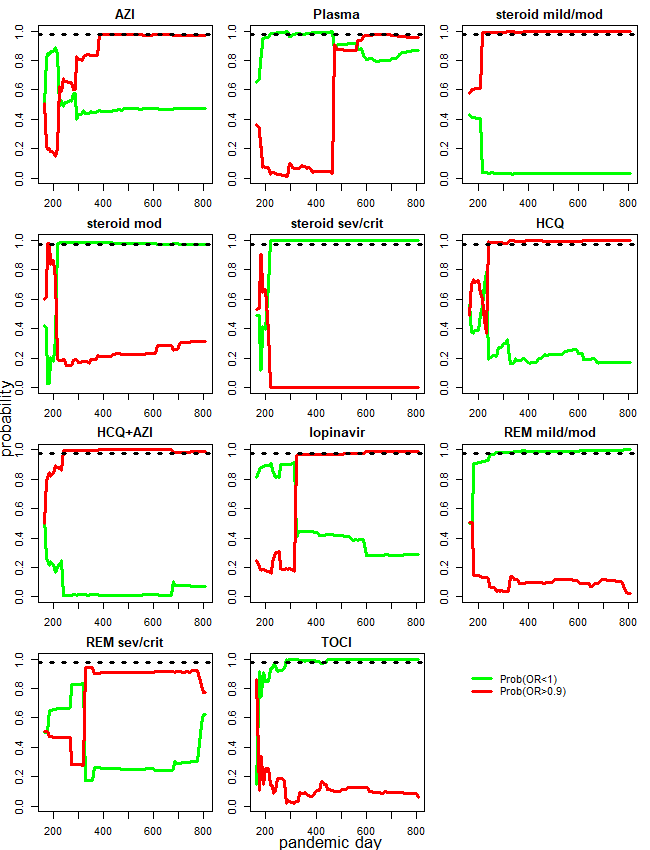

Acronyms: AZI = Azithromycin; HCQ = Hydroxychloroquine; REM = Remdesivir; OR = Odds Ratio

Figure S35. Probability of beneficial treatment effect (green line; odds-ratio<1) and no clinical benefit (red line; odds-ratio >0.9) as a function of day since start of pandemic based on RCTs only


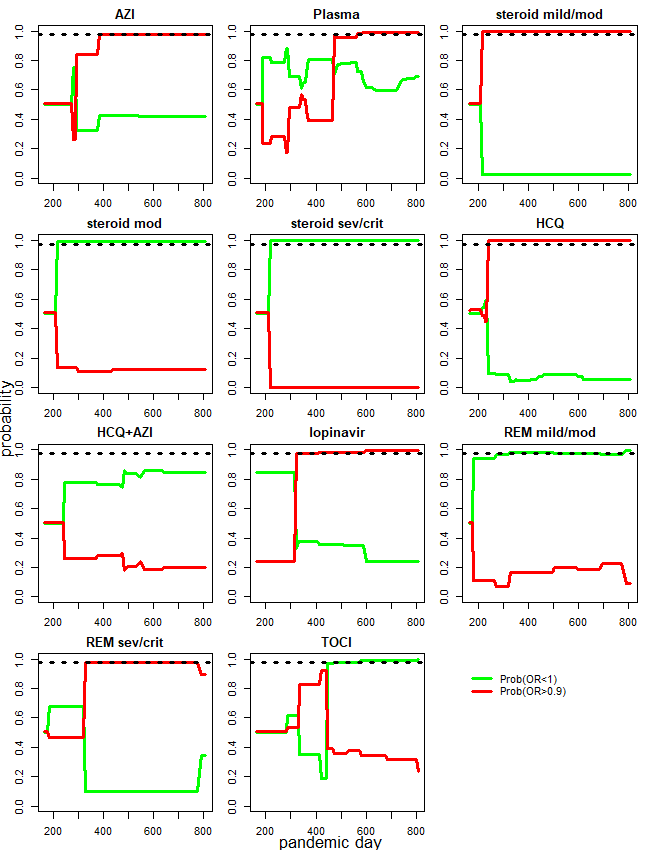


Acronyms: AZI = Azithromycin; HCQ = Hydroxychloroquine; REM = Remdesivir; OR = Odds Ratio
